# Natural anti-cancer products: insights from herbal medicine

**DOI:** 10.1186/s13020-025-01124-y

**Published:** 2025-06-09

**Authors:** Dianxin Cui, Cheng Zhang, Lili Zhang, Jingbin Zheng, Jie Wang, Luying He, Haochun Jin, Qianming Kang, Yang Zhang, Na Li, Zhenlong Sun, Wenying Zheng, Jinchao Wei, Siyuan Zhang, Yibin Feng, Wen Tan, Zhangfeng Zhong

**Affiliations:** 1 Macao Centre for Research and Development in Chinese Medicine, State Key Laboratory of Quality Research in Chinese Medicine, Institute of Chinese Medical Sciences, University of Macau, Avenida da Universidade, Taipa, Macao S.A.R., 999078 China; 2 School of Chinese Medicine, Li Ka Shing Faculty of Medicine, The University of Hong Kong, 6/F, 3 Sassoon Road, Pokfulam, Hong Kong S.A.R., 999077 China; 3 School of Pharmacy, Lanzhou University, Lanzhou, 730000 Gansu China

**Keywords:** Natural product, Herbal medicine, Chinese medicine, Chinese medicinal herb, Cancer

## Abstract

Herbal medicine exhibits a broad spectrum of potent anti-cancer properties, including the enhancement of tumor immune responses, reversal of multidrug resistance, regulation of autophagy and ferroptosis, as well as anti-proliferative, pro-apoptotic, and anti-metastatic effects. This review systematically explores recent advances (primarily documented since 2019) in research on key anti-cancer compounds derived from herbal medicine, such as apigenin, artemisinin, berberine, curcumin, emodin, epigallocatechin gallate (EGCG), ginsenosides, icariin, resveratrol, silibinin, triptolide, and ursolic acid (UA). These studies were sourced from scientific databases, including PubMed, Web of Science, Medline, Scopus, and Clinical Trials. The review focuses on the significant role that these natural products play in modern oncology, exploring their efficacy, mechanisms of action, and the challenges and prospects of integrating them into conventional cancer therapies. Furthermore, it highlights cutting-edge approaches in cancer research, such as the utilization of gut microbiota, omics technologies, synthetic derivatives, and advanced drug delivery systems (DDS). This review underscores the potential of these natural products to advance the development of novel anti-cancer treatments and support contemporary medicine. Additionally, recent multi-omics findings reveal how these compounds reshape transcriptional and metabolic networks, further broadening their therapeutic scope. Many natural products exhibit synergy with first-line chemotherapies or targeted therapies, thereby enhancing treatment efficacy and reducing side effects. Advanced nano-formulations and antibody–drug conjugates have also substantially improved their bioavailability, making them promising candidates for future translational research.

## Introduction

Cancer remains a global public health crisis, with over 20 million new cases and 9.7 million deaths around the world [1, 2]. It is caused by internal factors such as genetics [3–5], endocrine [6], and immunity [7], as well as external factors like chemicals [8], physics [9], unhealthy lifestyles [10], and aging-related factors [11]. Herbal medicine offers a multifaceted strategy for cancer therapeutics, emphasizing both tumor suppression and improving general health conditions [12, 13]. This includes the improvement of immune system function, reduction of side effects associated with conventional cancer treatments such as chemotherapy and radiation, and the holistic management of patient health [14, 15]. For example*, *research showed that the ethanol extract of *Marchantia polymorpha L.* (Common liverwort) suppressed the growth of liver cancer cells by triggering apoptosis via intrinsic and endoplasmic reticulum (ER) stress-associated pathways [16]. Besides, another study published in *Chinese Medicine* explored the effects of Cucurbitacin B on inhibiting transforming growth factor-beta 1 (TGF-*β*1)-induced epithelial-mesenchymal transition (EMT) in non-small cell lung cancer (NSCLC), revealing that Cucurbitacin B modulates ROS production and the phosphoinositide 3-kinase (PI3K)/AKT/ mammalian target of rapamycin (mTOR) signaling pathways [17]. Despite these promising advancements, the precise molecular targets of many phytochemicals in herbal medicine remain poorly defined, representing a significant barrier to their broader acceptance and integration into mainstream cancer treatment protocols.

**Fig. 1:**
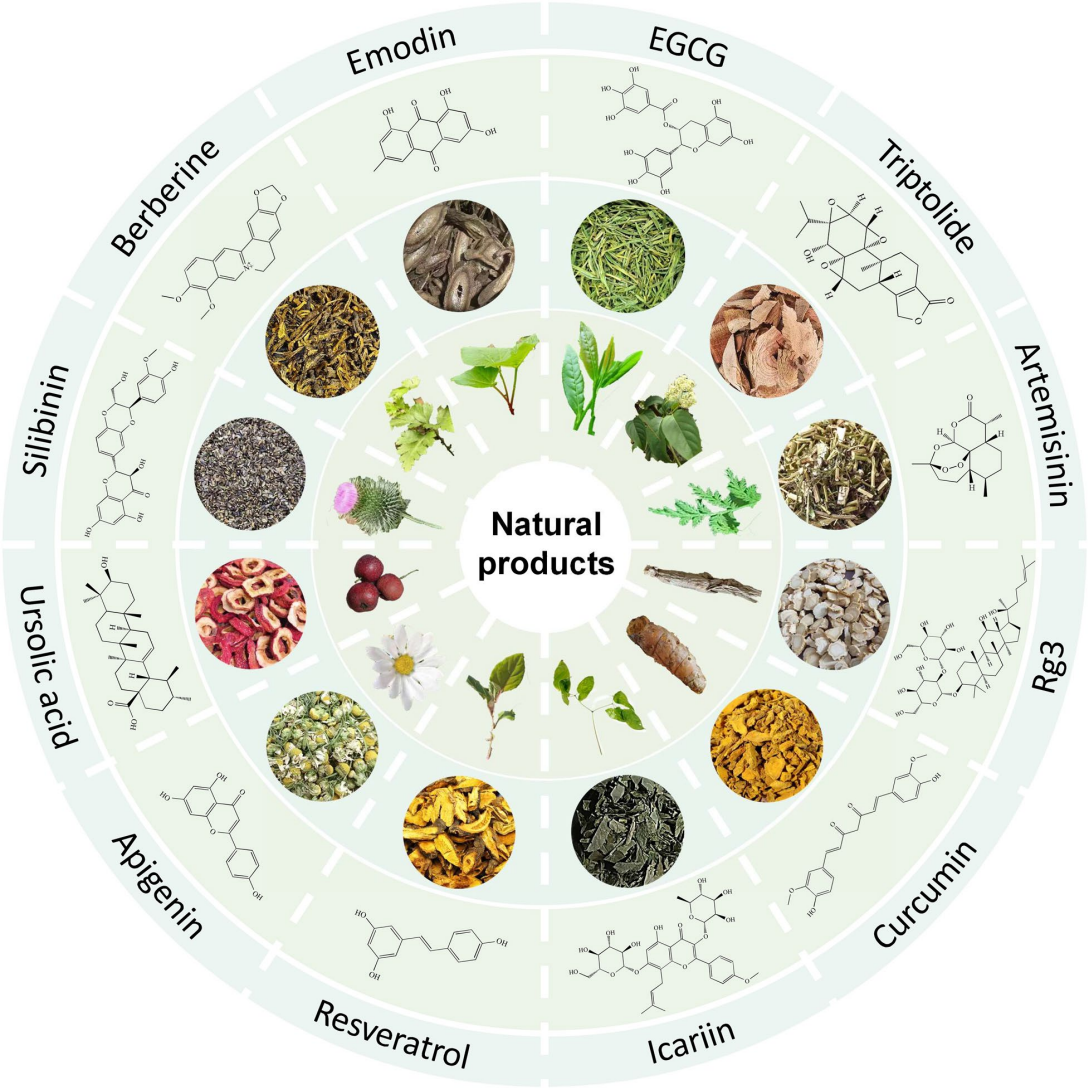
Natural compounds derived from herbal medicine with anti-cancer effects. The natural products are displayed with their chemical structures, herbal slices, and plant sources, including Apigenin, Artemisinin, Berberine, Curcumin, Emodin, Epigallocatechin gallate (EGCG), Ginsenoside Rg3, Icariin, Resveratrol, Silibinin, Triptolide, and Ursolic acid (UA)

**Fig. 2:**
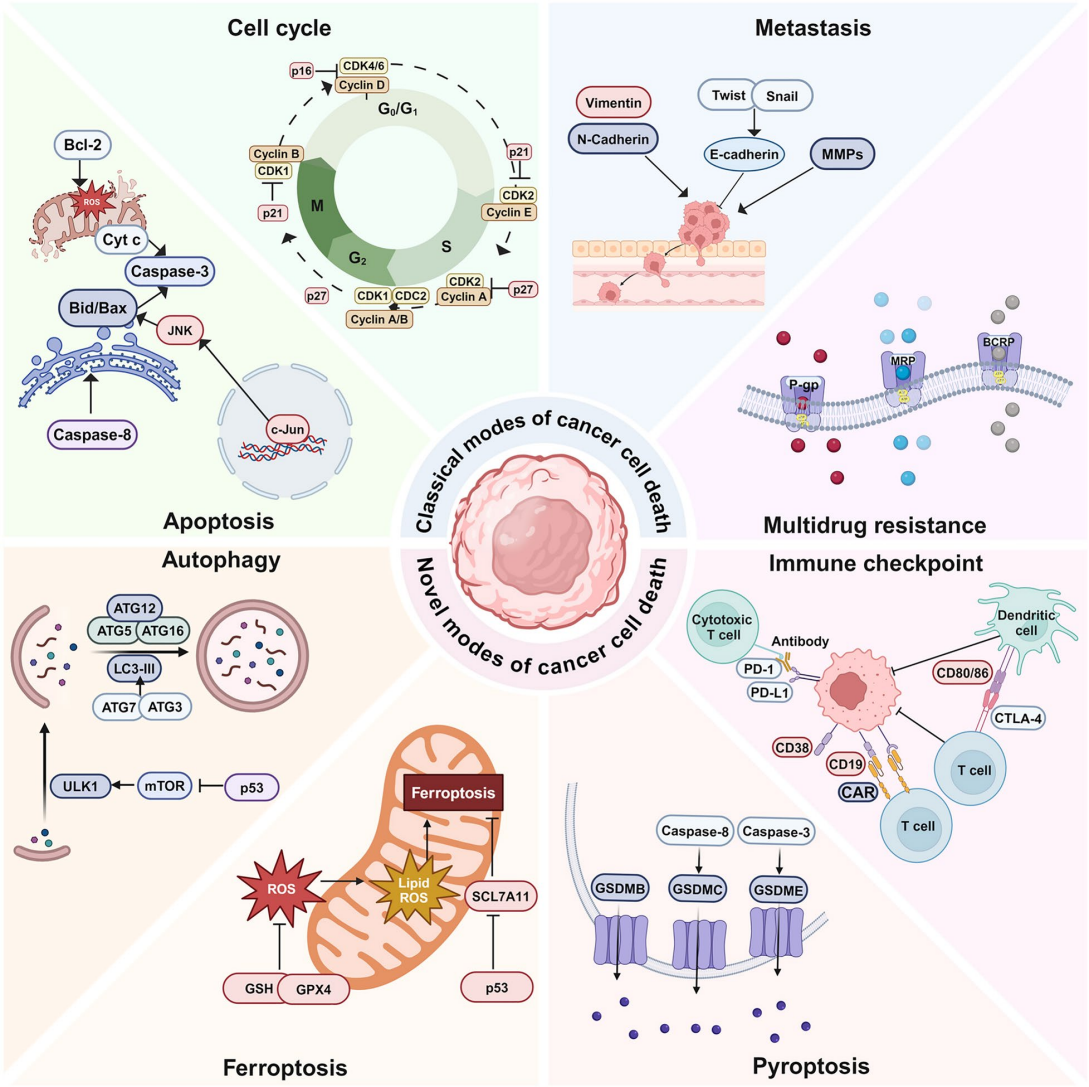
Key mechanisms in cancer biology, emphasizing both conventional and innovative ways of cancer cell death. Classical pathways include apoptosis, cell cycle, metastasis, and multidrug resistance, while novel pathways involve autophagy, ferroptosis, pyroptosis, and immune checkpoints. These processes highlight the adaptability of cancer cells and provide insights into potential therapeutic targets (Created with BioRender, https://BioRender.com/9bkp9cu)

**Fig. 3:**
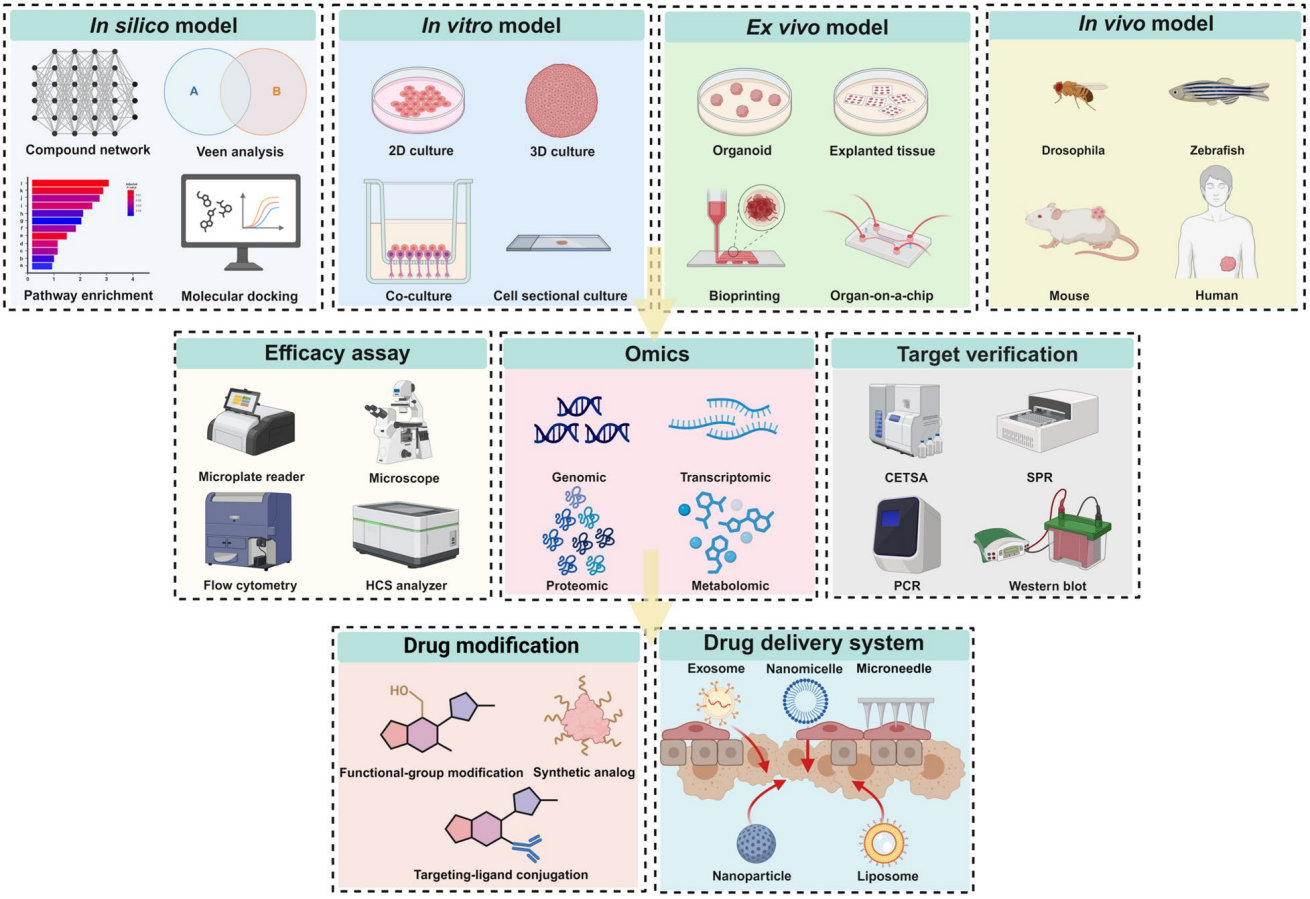
Research and development of natural anti-cancer compounds derived from herbal medicine. This figure comprehensively illustrates drug development models and techniques relevant to natural anti-cancer compounds, spanning from in silico simulations to in vivo experiments. It not only demonstrates the interconnections throughout the drug development process but also incorporates in vitro and ex vivo approaches, efficacy assays (e.g., HCS: High content screening), omics studies, target verification methods (e.g., CETSA: Cellular thermal shift assay, SPR: Surface plasmon resonance), drug modification strategies, and drug delivery systems, providing a holistic view of the entire drug development pipeline for natural anti-cancer compounds (Created with BioRender, https://BioRender.com/2a5479b)

This review provides an overview of the 12 most frequently studied natural anti-cancer products, including terpenoids [Artemisinin, Ginsenosides, Triptolide, and Ursolic acid (UA)], flavonoids (Apigenin, Icariin, and Silibinin), phenols [Curcumin, Epigallocatechin gallate (EGCG), and Resveratrol], alkaloids (Berberine), and anthraquinone (Emodin). Figure 1 illustrates the structure of natural products and their origination from herbal medicine. These compounds have demonstrated promising anti-cancer properties, including tumor immunity enhancement, reversal of multidrug resistance, regulation of autophagy and ferroptosis, and antiproliferative, pro-apoptotic, and anti-metastatic effects, both in vitro and in vivo (Fig. 2). Additionally, this review delineates the commonly employed disease models (in silico, in vitro, ex vivo, and in vivo models), pharmacological research methodologies (efficacy assays, omics technologies, and target validation), and modification strategies (including drug modification and drug delivery systems) utilized in anticancer research involving natural compounds (Fig. 3).

This review explores the intersection of shinning compounds from Chinese medicine and cancer research, focusing on the synthesis of experimental findings and scholarly conclusions mostly drawn from academic publications since 2019. These compounds have been reported in over 100 research publications, with studies conducted in both preclinical and clinical settings. The literature review was performed using several reputable databases, including PubMed, Web of Science, Medline, Scopus, and Clinical Trials. Search terms utilized included “Natural product”, “Herbal medicine”, “Chinese medicine”, “Chinese medicinal herb”, “Cancer”, “Tumor”, and “Neoplasm”. Our study synthesizes recent advances in herbal medicine-derived anti-cancer compounds by critically analyzing their pharmacological mechanisms, therapeutic potential, and integration challenges within oncology, aiming to provide clear insights.

### Apigenin

Apigenin, a flavonoid predominantly derived from *Matricaria chamomilla* (Chamomile), has garnered significant attention in recent years due to its broad spectrum of pharmacological properties such as anti-inflammatory, antioxidative, antidepressant effects, and cardiovascular protection [18–21]. Remarkably, apigenin has demonstrated efficacy against a diverse array of cancer types, including lung, breast, colon, bladder, head and neck, melanoma, prostate, kidney, cervical, thyroid, oral, pancreatic, and leukemia cancers [22–30]. It impedes cancer progression through various mechanisms, including modulation of tumor immunity, reversal of drug resistance, promotion of autophagy and ferroptosis, induction of apoptosis, and inhibition of invasion and metastasis [31–34]. In silico model employs advanced computational techniques, such as molecular docking, dynamics simulation, quantitative structure–activity relationship (QSAR), and quantum calculation, to assess apigenin derivatives as potential inhibitors of HPV-associated cervical cancer and DNA polymerase theta [35]. Apigenin is categorized under the biopharmaceutical classification system (BCS) class II, characterized by low solubility and high permeability [36–38]. Recent advancements in drug delivery, such as the application of the self-microemulsifying drug delivery system (SMEDDS) and microwave solid dispersion techniques, have significantly enhanced the bioavailability of Apigenin [39, 40]. Moreover, there is growing evidence that co-administration of Apigenin with other pharmacological agents may yield synergistic effects, enhancing therapeutic outcomes [41–43]. To serve as a guide for future research and clinical use, the following sections will briefly review the mechanisms, routes, and molecular targets through which Apigenin produces its anti-cancer actions.

### Apigenin improves tumor immunity

Apigenin demonstrates potent immunomodulatory effects across various cancer types through interactions with diverse immune cells, including dendritic cells (DCs), tumor-associated macrophages (TAMs), myeloid-derived suppressor cells (MDSCs), natural killer (NK) cells, and T lymphocytes [44–47]. Some recent studies show that high doses of apigenin can inhibit the proliferation of DCs, and can also reduce the protein expression of programmed death-ligand 1 (PD-L1) in DCs by inhibiting the phosphorylation of STAT1, thus inducing anti-cancer immunity [48, 49]. Apigenin also works by stimulating NK-92 cell proliferation [50]. Apigenin is also shown to reduce M2-like macrophage populations and promote M1 macrophage polarization in murine models, a mechanism potentially mediated by Src homology 2 domain-containing inositol 5-phosphatase 1 (SHIP1) upregulation. Furthermore, evidence indicates that apigenin suppresses C–C motif chemokine ligand 2 (CCL2) secretion, thereby inhibiting MDSCs infiltration into pancreatic tumors. This anti-invasive effect may be attributed to nuclear factor kappa-B (NF-*κ*B) signaling downregulation [45]. Therefore, apigenin exhibits promising potential as an immunotherapeutic agent in cancer treatment through its multi-target modulation of immune components. Key mechanisms include the inhibition of PD-L1 expression and the modulation of NF-*κ*B/SHIP-1 signaling pathway, which collectively enhance the efficacy of cancer immunotherapy (Table 1).

**Table 1:** List of anti-cancer natural compounds from Chinese herbal medicines

Compounds	Origins	Cancer types	In vitro models	In vivo models	Anti-cancer effects	Underlying mechanisms	Dosage	Combinational agents	References
Apigenin	Matricaria chamomilla (Yangganju)	Bladder cancer, breast cancer, cervical cancer, colon cancer, head and neck cancer, kidney cancer, leukemia cancer, lung cancer, melanoma, oral cancer, pancreatic cancer, prostate cancer, thyroid cancer	22Rv1, A2780, A375, A375SM, A549, AGS, BEL-7402, B16-F10, BEL-7402/ADM, BT-549, C33A, CAL-62, CNE2, CD18/HPAF, DU145, H358, H460, HCT 116, HCT-15, HeLa, HEp-2, Hep G2, HL-60, HONE1, HT-29, LOVO, LNCaP, MDA-MB-468, MCF-7, MCF-7/ADR, MDA-MB-231, MDA-MB-453, NCI-H929, NK-92, OVCAR4, PC-3, Panc02, RPMI-8226, SCC-9, SCC-25, SH-SY5Y, SiHa, SNU-638, SK-BR-3, SK-OV-3, SK-OV-3/DDP, SW480, T24, U266, UM-SCC-14C, UM-SCC-22B	A375SM Xenograft mice, A549 tumor-bearing nude mice, B16-F10 Xenograft mice, C33A Xenograft mice, CNE2 Xenograft mice, H358 Xenograft mice, HCT 116 Xenograft mice, HONE1 Xenograft mice, HepG2 Xenograft mice, Panc02 tumor-bearing mouse, PC-3 M Xenograft mice	Block cell cycle; improve tumor immunity; induce apoptosis, autophagy, and ferroptosis; reverse cancer drug resistance	Activate Caspase-9, Caspase-3; down-regulate ABCB1, Bcl-2, GLUT-1, HSP90, leukotriene C4, Mcl-1, MMP-2, MMP-9, NF-*κ*B, NRF2, p-AKT, p62, PD-L1; increase cytochrome C; inhibit AKT, EGFR, ERK1/2, PI3K/AKT, RAS-MAPK pathway, STAT3; Up-regulate LC3-II, miR-215-5p, p53, SHIP-1	10 μM, 20 μM, 30 μM, 50 μM, 70 μM, 0–30 μM, 0–40 μM, 0–50 μM, 0–60 μM, 0–80 μM, 10–80 μM, 0–100 μM, 0–200 μM; 0.8 mg/mouse/day, 3 mg/kg, 25 mg/kg, 30 mg/kg, 50 mg/kg, 150 mg/kg, 200 mg/kg, 300 mg/kg	Cetuximab, Cisplatin, Doxorubicin, Docetaxel, Paclitaxel	[22, 24–30, 45, 48–63, 67–75, 77–83, 1088–1100]
Artemisinin	Artemisia annua L. (Qinghao) or sweet wormwood	acute myeloid leukemia, bladder cancer, breast cancer, cervical cancer, colon cancer, endometrial cancer, esophageal cancer, glioblastoma, glioma, leukemia, liver cancer, myeloma, non-Hodgkin lymphoma, non-small cell lung cancer (NSCLC), ovarian cancer, pancreatic ductal adenocarcinoma, prostate cancer, renal cell carcinoma, salivary gland tumor	16HBE, 4T1, A-253/HTB-41, A2780, A549, A549/TAX, ARP1, AN3CA, B16-F10, BEL-7402, BT-474, BT-549, Caki-2, CT26, NCI-H1975, DLD-1, EJ, EC109, H1299, HCC1569, HCC1954, HCT 116, HCT-15, Hep G2, HepG2.2.15, Hep3B, HL-60, H929, Huh-7, HT-29, IOSE144, Ishikawa, K-562, KG-1, KOPN8, LCC9, Lewis, MGC-803, MHCC-97H, ML-2, MKN-28, MKN-45, MOLM-14, MDA-MB-231, MCF-7, MCF-7/ADR, MV-4–11, NCI-H460, OCI-AML2, OVCAR3, PC-9, PC9-GR1-AZD2, PC9-GR4-AZD1, PC9-OR3, PC9-OR5, Raji, SGC-7901, SNU-182, SNU-449, SK-OV-3, SW480, SW620, T-47D, T24, THP-1, TN22, UMRC-2, U118-MG, U251, U373, U87, U251, U937, YAC-1	4T1 xenograft mice, A2780 xenograft mice, A549 xenografts, B16-F10 xenograft mice, Caov-3-luc xenograft mice, colon cancer patient—derived xenografts, CT26 xenograft mice, DU 145 xenograft mice, HCC-LM3 xenograft mice, H1975 xenograft mice, H460 xenograft mice, Hep G2 xenograft mice, Huh-7 xenograft mice, HL-60 xenografts, Lewis tumor—bearing mice, MCF-7 xenograft mice, MC38 xenograft mice, MDA-MB-231 xenografts, MKN-45 xenografts, PC-9 xenograft mice, PC9-GR4- -AZD1 xenograft mice, PANC-1 xenograft mice, SGC-7901 xenograft mice, SMMC-7721SR xenograft mice, SKOV3 xenografts, T24 xenografts, UMRC-2 xenograft mice	Promote anti-angiogenesis; block cell cycle; enhance immune function; induce apoptosis, autophage, and ferroptosis; inhibit and migration; reverse multidrug resistance	Activate PARP1, Caspase-3; down-regulate Bcl-2, Bcl-xL, c-Myc, E-cadherin, N-cadherin, PD-L1, Snail, Vimentin; increase lipid ROS and malondialdehyde; inhibit 293 T, SU-DHL2, SU-DHL4, SU-DHL6, U2932 activation; inhibit STAT3 pathway; promote mitochondrial LC3B recruitment; reduce AFAP1L2 protein expression; up-regulate Bad, Bak, Bax, Bim, p53	0–5 µM, 0–20 µM, 0–30 µM, 0–40 µM, 10 μM, 25 μM, 30 μM, 50 μM, 0–80 μM, 0–100 μM, 0–120 μM, 0–140 μM, 0–150 μM, 0–160 μM, 0–200 μM, 0–300 μM; 2 mg/kg, 5 mg/kg, 8 mg/kg, 15 mg/kg, 30 mg/kg, 40 mg/kg, 50 mg/kg, 100 mg/kg	5-FU, Anlotinib, Capecitabine, Carboplatin, Cisplatin, Dexamethasone, Metformin, Navitoclax, Osimertinib, Oxaliplatin, Paclitaxel, Resveratrol, Sorafenib, Sulfampyridine, Trastuzumab, Tamoxifen, Venetoclax	[101–105, 115, 118–128, 133, 136, 139–157, 159, 161, 168, 172–176, 179, 180, 186–188, 1101–1103]
Berberine	Coptis chinensis (Huanglian)	Bladder cancer, breast cancer, cervical carcinoma, colon cancer, gastric carcinoma, glioma, leukemia, Liver cancer, lung cancer, melanoma, nasopharyngeal carcinoma, oral squamous cell carcinoma, ovarian cancer, osteosarcoma, renal cell carcinoma	4T1, 798-O, ACHN, A172, A2780, A-498, A549, B-CLL, BEL-7402, BGC-823, BT-549, CNE2, H1299, H1975, H358, H460, Hela, Hep G2, HCC827/AR, HT-29, HCT116, HL-60, Huh7, Jurkat, K-562, Lewis, LN-18, LN-229, MCF-7, MCF-7/DOX, MDA-MB-231, MG-63, MKN-45, OVCAR3, SGC-7901, SK-OV-3, SW480, T24, T98G, Tca8113, U-138, U251, U87, U118-MG, U-2 OS, U-87 MG, 5637	786-O xenografts, A549 xenograft mice, AOM/DSS model, BGC-823 xenograft mice, HepG2 xenografts, HL-60 xenografts, HCC827/AR xenograft mice, Lewis tumor-bearing mice, MCF-7/DOX xenograft mice, MDA-MB-231 xenograft mice, MG-63 xenografts, MKN-45 xenografts, SK-OV-3 xenografts, U-87 MG xenograft mice	Block cell cycle; induce apoptosis and autophagy, suppress epithelial-mesenchymal transition; modulate cancer immunity; suppress drug resistance; suppress proliferation, migration, and invasion; synergistic anti-cancer effects	Activate AMPK, Caspase-3, and Caspase-9; disrupt mitochondrial membrane potential; down-regulate Bcl-2, Bcl-2c, cyclin D1, Mcl-1, mir-21, MRP1, P-glycoprotein (P-gp), PD-L1, ROR1; inhibit HER2/PI3K/AKT pathway, HIF-1*α*, MMP-2 and MMP-9 activity, TGF-*β*1/COL11A1 activity, TGF-*β*1/SMAD2/3 pathway; inactivate AKT/mTOR pathway; promote IGF2BP3 ubiquitination; up-regulate Bad, Bak, Bax, Bim, Cytochrome, p21, p27, ROS	5 μM, 10 μM, 0—30 μM, 0–40 μM, 0–80 μM, 0–108 μM, 0–200 μM, 0–400 μM, 0–500 μM, 47–12,000 μM, 0–50 μg/mL, 0–100 μg/mL, 10 μg/mL, 50 μg/mL, 0–125 μg/mL, 100 μg/mL; 1.5 mg/kg, 5 mg/kg, 10 mg/kg, 20 mg/kg, 25 mg/kg, 0–32 mg/kg	5-FU, Carboplatin, Cisplatin, Curcumin, Adriamycin, Doxorubicin, Osimertinib, Oxaliplatin, Paclitaxel, Sorafenib, Sunitinib, T-DM1	[210, 213–231, 236, 239, 241–243, 251]
Curcumin	Curcuma longa L. (Jianghuang) and Curcumae rhizoma (Ezhu)	Bladder cancer, breast cancer, cervical cancer, colon cancer, glioblastoma, head and neck cancer, kidney cancer, leukemia, liver cancer, lung cancer, melanoma, non-hodgkin lymphoma, ovarian cancer, osteosarcoma, pancreatic cancer, prostate cancer, renal cell carcinoma, thyroid cancer, tongue carcinoma	786-O, ACHN, A172, A2780, A-498-DR, A549, A549/Cisplatin, B16-F10, BT-549, CAL 27, CT26, FTC-133, FTC-238, GL261, H1299, H157, H1650, H22, HCT-8, HCT 116, HSC-3, Huh-7, HL-60, HepG2, HepG2215, IEC-18, K-7, K-562/DOX, KB-V1/DOX, L-540, MC38, MCF-7, MDA-MB-231, MDA-MB-453, MIA PaCa-2, MG-63, MKN-45, NCI-H1975, PA1, PANC-1, PLC, RT4, Saos-2, SCC-15, SiHa, SKBR3, SK-HEP-1, SK-OV-3, SNU1041, SW480, SW620, SGC-7901, T-47D, TCCSUP, U-2 OS, U-87MG	A549 xenografts, AOM/DSS model, 4T1 xenograft mice, ACHN xenograft mice, B16-F10 xenograft mice, Ca Ski xenograft mice, CT26 xenograft mice, H1299 xenograft mice, H157 xenograft mice, H1975 xenograft mice, Hep G2 xenograft models, HL-60 xenografts, Lewis tumor-bearing models, MC38 xenograft mice, MDA-MB-231 xenografts, MG-63 xenografts, MKN-45 xenografts, PBMEC xenograft mice, SK-OV-3 xenografts, U87 orthotopic, 786-O xenografts	Induce cell cycle arrest; inhibit migration, invasion; modulate host immunity; promote autophagy, ferroptosis, apoptosis; regulate gut microbiota; reverse multidrug resistance; synergistic effects	Activate Caspase-3, Caspase-8, Caspase-9, and PARP; decrease LC3-II/LC3-I proportion; down-regulate Bcl-2, Bcl-xL, Bcr-Abl, BCRP, CDK2, cyclin A2, cyclin E1, DNMT1, EGFR, FTH1, GPX4, MRP, MMP-9, NCOA4, PD-L1, P-gp, p21, SLC7A11, TWIST1, VEGF; regulate ATG2B, ATG5, Bad, Bax, Beclin-1, LC3B, miR-196b, p53, p62, PTEN, ROS, TCF21	5 μM, 10 μM, 0–40 μM, 0–50 μM, 0–80 μM, 0–100 μM, 10–15 μM, 0–9.6 μg/ml, 0–10 μg/ml; 1.2 mg/kg, 2 mg/kg, 5 mg/kg, 40 mg/kg, 50 mg/kg, 100 mg/kg, 120 mg/kg, 240 mg/kg, 1 g/kg	5–Fluorouracil, Atezolizumab, Carboplatin, Cetuximab, Cisplatin, Celecoxib, Doxorubicin, Erlotinib, Folinic Acid, Gefitinib, Lenvatinib, Oxaliplatin, Paclitaxel, Pembrolizumab, Rapamycin, Sulphasalazine	[283–288, 297–301, 303, 304, 307–311, 314–319, 323–325, 328, 329, 332–335, 337–340, 347–362, 370–386, 395, 399, 1104–1111]
Emodin	Rheum officinale Baill. (Dahuang); Polygonum cuspidatum (Hu Zhang)	Breast cancer, cervical cancer, colon cancer, endometrial cancer, gastric cancer, glioma, kidney cancer, leukemia, liver cancer, lung cancer, melanoma, nasopharyngeal cancer, neuroblastoma, ovarian cancer, pancreatic cancer, prostate cancer, skin cancer, thyroid cancer	786-O, 4T1, ACHN, A2780, A375, A549, A549/DDP, AU565, B16-F10, BT-20, BGC823, BxPC-3, Caco-2, CCD-19Lu, DU 145, HeLa, H1299, H1650, H1975, H460, HCT 116, HCT-15, HT-29, Huh-7, Hep G2, IHH4, K-562, K-562/ADM, KLE, MDA-MB-231, MDA-MB-453, MGC803, MIA-PaCa-2, MUG-Mel2, NCI-H460, NCI-H520, OVCAR-3, PA1, PC-3, PC-9, PANC-1, RKO, SMMC-7721, SCC-25, SK-MEL-28, SK-OV-3, SW480, SW480/5-FU, SW620, TPC-1, U251, U87MG	4T1 xenograft mice, A549 xenograft mice, HCT 116 xenograft mice, HT-29 xenograft mice, HepG2 xenograft mice, KLE xenograft mice, LLC xenograft mice, MCF-7 xenograft chick embryos, MDA-MB-231 xenograft mice, NA xenograft mice, PANC-1 xenograft mice, PC-3 xenograft mice, SK-OV-3 xenograft mice, SW480/5-Fu xenograft mice, SW620 xenograft mice, SMMC-7721 xenograft mice, TPC-1 xenograft mice, U251 xenograft mice	Combine with other medications; inhibit EMT; induce necroptosis, autophagy, ferroptosis, uncoupling, cell cycle arrest; inhibit angiogenesis, migration, invasion; modulate tumor immunity	Activate AMPK, AhR-CYP1A1 pathway, Caspase-3, Caspase-7, PGAM; down-regulate Bcl-2, Bcr-Abl, c-Myc, Fasn, Gpat1, GLUT1, GPX4, GST, MRP, miR-371a-5p, P-gp, p53, Scd1, STAT3, STAT5, xCT; inactivate NF-*κ*B, VEGFR2-AKT-ERK1/2 pathway, Wnt/*β*-catenin; inhibit PTHLH expression, SIK3 activity, SP1; reduce ATP, mitochondrial membrane potential; regulate Bax, E2F1, HSP90AA1, MLKL, MMP-2, MMP-9, PTEN, RIP1, RIP3, ROS, SerRS, TNF-*α*	5 μM, 20 μM, 40 μM, 0–15 μM, 0–40 μM, 0–50 μM, 0–75 μM, 0–80 μM, 0–100 μM, 0–120 μM, 0–240 μM, 0–250 μM, 0–300 μM, 2 μM-200 μM, 8 μg/ml, 15 μg/mL, 16 μg/ml, 0–40 μg/ml; 10 mg/kg, 20 mg/kg, 25 mg/kg, 40 mg/kg, 50 mg/kg, 80 mg/kg, 100 mg/kg, 400 mg/kg	3'‑Azido‑3'‑Deoxythymidine (AZT), Adriamycin, BIBR1532, Berberine, Carfilzomib, Celecoxib, Cisplatin, Cyclophosphamide, Daunorubicin, Doxorubicin, 5- Fluorouracil, Gemcitabine, Imatinib, Paclitaxel, Resveratrol, Temozolomide, Tetrahydropalmatin, Thymoquinone, Vinblastine, Vitamin	[440, 451–459, 462–476, 479–486, 488–494, 497, 498, 500, 502, 504–507, 510, 513, 516, 1112–1117]
Epigallocatechin gallate (EGCG)	Camellia sinensis (green tea)	Anaplastic thyroid carcinoma, bladder cancer, breast cancer, cervical cancer, colon cancer, endometrial cancer, esophageal cancer, gastric cancer, glioblastoma, head and neck cancer, leukemia, liver cancer, lung cancer, melanoma, oral squamous cell carcinoma, ovarian cancer, pancreatic cancer, prostate cancer, renal cell carcinoma, rhabdomyosarcoma, skin cancers	8505C, A375, A2780, A549, AGS, BGC-823, BxPC-3, CAL27, Caki-1, CNE-2, CRL-1739, DU 145, DLD-1, GL261, H1975, H22, H358, H460/PT, HCT 116, HCT-15, HeLa, Hep G2, HL-60, HL-60/MX2, HSC-3, Huh-7, H1299, HT-29, KB-A1, K-562, KYSE-150, LCC6/MDR, MCF-7, MCF 10A, MDA-MB-231, MIA-PaCa-2, OVCAR3, PANC-1, PMECs, PC-3, PC-9, RH30, SGC-7901, SiHa, SK-OV-3, SKBR3, SW480, TSGH 9201, TU212, T47-D, TE-1, U87	A549 xenograft mice, BEL-7404/DOX xenograft mice, B16-F10 xenograft mice, CNE1 xenograft mice, CT26 xenograft mice, CAL27 xenograft mice, DEN intraperitoneally injected rats, HCT 116 xenograft mice, HT-29 xenograft mice, Hep G2 xenograft mice, HGC-27/R xenograft mice, HSC-3 xenograft mice, Huh-7 xenograft mice, LCC6/MDR xenograft mice, MKN-45/R xenograft mice, TU212 xenograft mice	Modulate tumor immunity; modify mRNA and miRNA expression; reduce cell proliferation, induce cell cycle arrest, promote apoptosis, inhibit migration and invasion, block angiogenesis; reverse tumor drug resistance; synergistic effects	Activate Caspase-3; down-regulate Bcl-2, Bcl-xL, CCNB, CCNB2, CCND, CDC25A, P-gp; suppress AMPK, PI3K/AKT/mTOR, STAT3/CXCL8 pathways; up-regulate Bax, Cxcl3, Col8a1, Cytochrome c, Met, MMP12, Oasl2, PTEN, SERPINB2	0–1000 nM, 0–40 μM, 0–80 μM, 0–100 μM, 100–600 μM, 0–400 μM, 0–50 μg/ml, 40–150 μg/ml, 25–200 μg/ml, 0–150 μg/ml, 50 μg/mL; 30 μg/kg, 10 mg/kg, 25 mg/kg, 30 mg/kg, 40 mg/kg, 50 mg/kg, 75 mg/kg, 80 mg/kg, 100 mg/kg, 125 mg/kg, 200 mg/kg	5-Fluorouracil, Celecoxib, Cilantro, Cisplatin, Daunorubici, Doxorubicin, Gefitinib, Oxaliplatin, Paclitaxel, Resveratrol, Sorafenib, Vitamin D	[519, 527, 528, 532–543, 546–554, 558–560, 563–572, 578, 579, 581, 586, 592–594, 598, 599, 1118–1125]
Ginsenosides(Rg3, Rh2, Compound K)	Panax ginseng (Renshen)	Rg3: Breast cancer, cervical cancer, colon cancer, gastric cancer, gliomas, lung cancer, melanoma, osteosarcoma, pancreatic cancer Rh2: Breast cancer, cervical cancer, colon cancer, esophageal cancer, liver cancer, melanoma, oral cancer, osteosarcoma, pancreatic cancer, prostate cancer Compound K: Esophageal cancer, liver cancer, lung cancer, prostate cancer	Rg3: A375.S2, A549, AGSR-CDPP, B16, BEL-7404, HCC827, HeLa, Hep G2, L3.6pl, LoVo, MG-63, MDA-MB-231, MCF-7/TamR, NCI-H1299, PC-3, PC-9, SMMC-7721, SPC-A-1, SW620, T-47D/TamR, U-2 OS Rh2: 4T1, A375, B16-F10, Ca9-22, C-33A, DLD-1, ECA109, G-361, HEK 293 T, HeLa, HCT 116, HCT-15, MC38, MDA-MB-231, PANC-1, Panc02, TE-13, U-2 OS, YD10B Compound K: 293 T, A549, BEL-7404, DU 145, ECA109, HeLa, Hep G2, Huh-7, LNCaP, Lewis, PC-3, SK-HEP-1, SMMC-7721	Rg3: B16-F10 xenograft mice, Orthotopic C6 glioma-bearing mice, subcutaneous MCF-7/T tumor mice, SW620 xenograft mice Rh2: 4T1 tumor-bearing mice, A375 xenograft mice, MC38 tumor-bearing mice Compound K: DEN-induced rats, Lewis tumor-bearing mice	Improve tumor immunity; induce cell cycle arrest, apoptosis; inhibit migration and invasion; regulate autophagy, ferroptosis; regulate gut microbiota; reverse drug resistance	Rg3: Activate Apaf1/Caspase-9/Caspase-3 pathway; down-regulate Bcl-2, MMP-2, MMP-7, MMP-9, PTX3; inhibit EMT; inactivate IL-6/STAT3/p-STAT3 pathway; promote p53 demethylation, p21 and p16 histone acetylation; up-regulate Bax, CASC2, PTEN Rh2: down-regulate AKT/mTOR, Bcl-2, P-gp Compound K: Activate Caspase-3, Caspase-9; activate VEGF-A/PI3k/AKT pathway	Rg3: 0–75 μM, 0–100 μM, 0–150 μM, 130 μM, 0–1 mM, 0.02–20 μg/mL, 0.08–20 μg/mL, 0.16–40 μg/mL, 1–15 μg/mL, 12.5 μg/mL, 25 μg/mL, 0–100 μg/mL, 0–200 μg/mL; 0.3 mg/kg, 1 mg/kg, 3 mg/kg, 20 mg/kg, 40 mg/kg, 200 mg/kg Rh2: 0–10 μM, 0–60 μM, 0–10 μg/mL, 30 mg/kg, 60 mg/kg Compound K: 0–10 μM, 0–60 μM, 0–80 μM, 0–150 μM, 0–30 μg/mL; 2.5 mg/kg, 5 mg/kg, 40 mg/kg	Rg3: 5-Fluorouracil, Anti-PD-L1 antibodies, Cisplatin, Curcumin, Doxorubicin, Gefitinib, Gemcitabine, Icotinib, Osimertinib, Oxaliplatin, Paclitaxel, Sorafenib, Tamoxifen Rh2: Cisplatin, Gemcitabine	[621–625, 629, 634, 635, 639, 641, 643, 645–657, 659, 662–670, 674–678, 1126]
Icariin/ Icaritin	Epimedium brevicornum (Yinyanghuo)	Icariin: Breast cancer, cervical cancer, colon cancer, gallbladder cancer, lung adenocarcinoma, lung cancer, liver cancer, ovarian cancer, oral squamous cell carcinoma, osteosarcoma, pancreatic cancer, prostate cancer Icaritin: Breast cancer, cervical cancer, colon cancer, endometrial carcinoma, esophageal cancer, laryngeal cancer, liver cancer, lung cancer, lymphoma, multiple myeloma, nasopharyngeal carcinoma	Icariin: 4T1, A549, A2780, CAL27, DU 145, GBC-SD, H1975, HeLa, HCT 116, Hs 578 T, HepG2, LNCap, MCF-7/ADR, MCF-7/TAM, MDA-MB-231, MDA-MB-468, MG-63, NOZ, Panc02, PC-3, PC-9, RM-1, SK-OV-3, SKVCR, SiHa, SMMC-7721, SCC-9, U-2 OS Icaritin: 4T1, A2780, A549, B16-F10, CAL 27, CNE-2, ECA109, H1975, HNE1, HONE1, HeLa, Hep G2, Hep G2/ADR, Hepa1-6, Huh-7, HT-29, HCT 116, H460, HO8910, Ishikawa, Lewis, MCF-7, MDA-MB-231, MDA-MB-468, MG-63/DOX, NK-92 MI, OPM-2, PC-3, PLC/PRF/5, RL95-2, RPMI 8226, SCC-9, SH-SY5Y (TDP-43-transfected), SK-Hep1, SNT-8, SNK-10, SNT-10, SMMC-7721, SW480, SUNE-1, U266	Icariin: 4T1 tumor-bearing mice, A549 xenograft mice, CAL27 xenograft mice, GBC-SD xenograft mice, H1975 xenograft mice, HeLa xenograft mice, MCF-7 xenograft mice, MDA-MB-231 xenograft mice, MG-63 xenograft mice, Panc02 tumor-bearing mice, P815 tumor-bearing mice, PC-3 bone metastasis model, RM1-Luc bone metastasis mice, SK-OV-3 xenograft mice, U14 xenograft mice Icaritin: 4T1 xenograft mice, 4NQO induced OSCC mice, A549 xenograft mice, B16-F10 xenograft mice, CNE-2 xenograft mice, Hepa1-6 orthotopic tumor mice and subcutaneous tumor mice, Hep G2 xenograft mice, HCT 116 xenograft mice, Ishikawa xenograft mice, MC38 tumor—bearing mice, NK-92 MI xenograft mice, RPMI 8226 xenograft mice, U266 xenograft mice	Improve tumor immunity; induce cell cycle arrest, apoptosis; inhibit migration and invasion; regulate autophagy; regulate gut microbiota; reverse tumor drug resistance; synergistic effects	Icariin: Activate Caspase-3, Caspase-8, Caspase-9, DR4, DR5, PARP, TNFR-1; down-regulate cyclin D1, P-gp, TGF-*β*1, Vimentin; release Cytochrome C; up-regulate E-cadherin Icaritin: Activate Caspase-8, DR4, DR5, PI3K/AKT pathway, TNFR-1; degrade HSP60, MNF1, MNF2, TiM23, TOM20; down-regulate MDR1, MRP1, PD-L1, P-gp, Vimentin; release Cytochrome C; up-regulate CD8, CXCL9, CXCL10, E-cadherin, FYN, IFN-γ, p21	Icariin: 10 μM, 20 μM, 40 μM, 0–40 μM, 0–50 μM, 0–80 μM, 0–100 μM, 20 μg/ml, 0–30 μg/ml, 0–80 μg/mL, 0–160 μg/mL; 10 mg/kg, 20 mg/kg, 40 mg/kg, 80 mg/kg, 100 mg/kg, 120 mg/kg Icaritin: 0–16 μM, 0–20 μM, 0–32 μM, 0–50 μM, 10–50 μM, 0–75 μM, 0–100 μM; 0.2 mg/kg, 3 mg/kg, 6 mg/kg, 10 mg/kg, 20 mg/kg, 30 mg/kg, 60 mg/kg, 70 mg/kg, 120 mg/kg	Icariin: Adriamycin, Cisplatin, Doxorubicin, Gemcitabine, P815AB Peptide, Tamoxifen Icaritin: Adriamycin and Doxorubicin, Anti-PD-1 antibodies, Cisplatin, Doxorubicin	[698, 699, 702, 704, 706–714, 716–718, 720–749, 753, 773, 775, 1127]
Resveratrol	More than 70 plant species, such as grapes, blueberries, raspberries	Bladder cancer, breast cancer, cervical carcinoma, colon cancer, head and neck cancer, lymphoma, luminal squamous cell carcinoma, multiple myeloma, melanocytoma, lung cancer, oral squamous cell carcinoma, ovarian carcinoma, pancreatic cancer, thyroid cancer, renal cell carcinoma, uterine leiomyoma	786-O, A2780, A549, B16, BCPAP, BxPC-3, Ca Ski, Caco-2, CAL-27, ELT3, ES-2, FaDu, HCT 116, HeLa, HT-29, KTC-1, MCF 10A, MCF-7, MCF-7/ADR, MDA-MB-231, MIA PaCa-2, NK-92, PANC-1, PE/CA-PJ49, RPMI 8226, SW1990, SCC-9, SCC-25, SiHa, SK-BR-3, SK-OV-3, SW480, T24-GCB, TPC-1, TUBO, TOV-112D, TOV-21G, U266	BxPC-3 subcutaneous tumor mice, ELT-3-LUC xenograft mice, HeLa xenograft mice, KTC-1 xenograft mice, LLC tumor-bearing mice, SGC7901 subcutaneous tumor mice, TPC-1 xenograft mice	Enhance tumor immunity; induce apoptosis; induce cell cycle arrest, inhibit proliferation, metastasis; regulate autophagy, ferroptosis; reverse tumor drug resistance	Activate Caspase-3, Caspase-8, Beclin-1 and LC3; down-regulate Bcl-2, fibronectin, GPX4, N-calmodulin, SLC7A11, vimentin; enhances p53 activity; Increase AMPK phosphorylation; Induce oxidative DNA damage; inhibit EMT; up-regulate Bax, CHOP, E-calmodulin, p15	5 μM, 15 μM, 50 μM, 75 μM, 0–80 μM, 0–100 μM, 150 μM, 0–150 μM, 0–200 μM, 0–60 μg/mL, 0–90 μg/mL, 0–100 μg/mL; 10 mg/kg, 25 mg/kg, 30 mg/kg, 50 mg/kg, 125 mg/kg	5-Fluorouracil, Cisplatin, Cetuximab, Curcumin, Doxorubicin, Oxaliplatin, Paclitaxel, Rapamycin	[787, 795, 800, 801, 813, 827, 828, 831–833, 838, 846–858, 860–863, 1128–1135]
Silibinin	Silybum marianum L. Gaertn. (Shuifeiji)	Breast cancer, cholangiocarcinoma, colon cancer, epidermal cancer, glioma, lung cancer, liver cancer, melanoma, nasopharyngeal carcinoma, oral squamous cancer, ovarian clear-cell carcinoma, pancreatic cancer, prostate cancer, renal cell carcinoma	786-O, 4T1, A2780, A431, A549, ACHN, B16-F10, BT-549, BxPC-3, Caki-1, Ca9-22, CaCo-2, C4-2, C666-1, CCLP-1, CNE-2, CT26, CAL-27, DU 145, ES-2, HT-29, H1299, H2228, H292, H3122, H460, HAC-2, HuCCT-1, HCT 116, HepG2, Huh-7, LoVo, LNCaP, MDA-MB-231, MDA-MB-468, MCF-7, MIA PaCa-2, NCI-H1975, NCI-H460, OVISE, PANC-1, PC-3, PC-9, RBE, RMG-1, SUNE-1, SCC-9, SCC-25, SW480, SW620, SMMC-7721, T-47D, TPC-1, TFK-1, TOV-21G, U87MG, U251, YD10B	4T1 tumor-bearing mice, A2780/DDP xenograft mice, A549 tumor-bearing mice, B16-F10 tumor-bearing mice, Ca9-22 xenograft mice, C6 xenograft mice, DEN/AAF induced rat, 786-O xenograft mice, HT-29 xenograft mice, HCT116 xenograft mice, MDA-MB-231 xenograft mice, MCF-7 xenograft mice, HepG2 xenograft mice, LA795 xenograft mice, PC-3 xenograft mice	anti-angiogenesis; epigenetic modifications; improve tumor immunity; induce apoptosis, cell cycle arrest, reduce proliferation and migration; regulate autophagy, ferroptosis; reverse tumor drug resistance	Activate Caspase-3, Caspase-9, JNK/c-Jun, and JNK/SAPK pathways; decrease phosphorylated mTOR; down-regulate ACSL4, Bcl-2, Er*α* pathway, miR-17–92 cluster, Ob-R, OPA1, PD-L1, RAC1, Wnt/*β*-catenin; increase eNOS phosphorylation; inhibit STAT3 activity; release Cytochrome c; suppress HIF-1*α*; up-regulate ATP content, Bax, Bcl2L11, CRT, DRP1, GSTP1, HMGB1, HSP70, MFN1, miR-19b, NQO1, NRF2, p21, p53, PTEN, PGC1*α*, TFAM	50 μM, 100 μM, 0–80 μM, 0–100 μM, 0–120 μM, 0–140 μM, 0–200 μM, 0–250 μM, 50–400 μM, 0–270 μM, 0–300 μM, 0–400 μM, 0–700 μM, 0–1000 μM, 10 μg/mL, 0.0014–70 μg/mL, 0–250 μg/mL; 5 mg/kg, 10 mg/kg, 50 mg/kg, 100 mg/kg, 150 mg/kg, 200 mg/kg	Brigatinib, Cisplatin, Curcumin, Dacarbazine, Epigallocatechin-3-Gallate, Etoposide, IPI-549, Lorlatinib, Metformin, Methotrexate, Neratinib, Nintedanib, Paclitaxel	[878, 882, 884, 889, 890, 895, 897, 898, 900, 901, 905–911, 915, 918, 919, 922–927, 929, 931–938, 947, 950, 954, 1136]
Triptolide	Tripterygium wilfordii (Leigongteng)	Acute Myeloid Leukemia, bladder cancer, breast cancer, colon cancer, erythroleukemia, esophageal squamous cell cancer, gastric cancer, glioblastoma, glioma, leukemia, laryngocarcinoma, liver cancer, lung cancer, melanoma, multiple myeloma, nasopharyngeal carcinoma, oral squamous cell carcinoma, ovarian cancer, pancreatic cancer, pituitary adenoma, prostate cancer, renal cell carcinoma	5637, A172, A375, A549, AGS, AtT-20, BT-549, B16-F10, CT26, CNE-2, CAL-27, DU145, HL-60, H1299, H1975, HEp-2, HT-29, HCT 116, K-562, KYSE-150, KYSE-180, LN-18, LN-229, MDA-MB-231, MKN-45, MIA PaCa-2, NPC-TW 039, NPC-TW 076, PANC-1, PC-3, PC-9, RPMI 8266, SK-OV-3, SK-OV-3/DDP, SW480, SCC-9, SGC-7901, SUNE-1, TE-1, T98G, TtT/GF, U251, U251-MG, U87, U-87 MG	3LL xenograft mice, 4T1 xenograft mice, A549 and A549/PR xenograft mice, A549/siE-cad xenograft mice, A549/TaxR xenograft mice, AGS and MKN45 tumor—bearing mice, AtT-20 xenograft mice, B16-F10 tumor—bearing mice, CT26 tumor-bearing mice, DMH/DSS induced mice, H1299 and UM-SCC6 xenograft mice, H1975 xenograft mice, KYSE-150 xenograft mice, Lewis lung carcinoma-grafted mouse model, MDA-MB-231 tumor—bearing mice, oral cancer PDTX models, SK-OV-3/DDP xenograft mice, TtT/GF xenograft mice, TS603 xenograft mice, U87 xenograft mice, U251 tumor-bearing mice	Promote anti-angiogenesis, epigenetic modifications, improve tumor immunity; induce apoptosis, cell cycle arrest, reduce proliferation and migration, regulate autophagy, ferroptosis, reverse tumor drug resistance	Block NF-*κ*B activation; decrease STAT3 phosphorylation; down-regulate ADAM12, Bcl-2, CD126, CD47, CDK4, CDK6, c-Myc, cyclin D1, CXCL12, Foxp3, IFN-γ, IL-10, Mcl-1, MMP-2, MMP-9, P-gp, PD-L1, Sorcin, VEGF; enhance p53 stability; inhibit AKT/mTOR pathway, HNF1A/SHH, IL-6R-JAK/STAT, Keap1/NRF2; up-regulate Bax, E-cadherin, p21	0–50 nM, 0–60 nM, 0–100 nM, 100 nM, 0–200 nM, 0–300 nM, 0–500 nM, 0–600 nM, 0–1000 nM, 0–32 ng/ml, 0–400 ng/mL; 0.1 mg/kg, 0.1 mg/kg, 0.125 mg/kg, 0.15 mg/kg, 0.2 mg/kg, 0.25 mg/kg, 0.25 mg/kg, 0.3 mg/kg, 0.4 mg/kg, 0.5 mg/kg, 0.8 mg/kg, 1 mg/kg, 50 mg/kg	10-Hydroxycamptothecin, ABT-199, Aspirin, Cetuximab, Cisplatin, Doxorubicin, Erlotinib, Gefitinib, Icotinib, Minnelide, Paclitaxel	[962, 971–994, 1001–1014, 1020, 1021, 1137–1141]
Ursolic acid (UA)	Crataegus pinnatifida (Shanzha)	Adult T-cell leukemia, breast cancer, colon cancer, esophageal squamous cell carcinoma, liver cancer, lung cancer, melanoma, ovarian cancer, osteosarcoma, papillary thyroid carcinoma, prostate cancer, stomach cancer	143B, A375, A2780, A549, AGS, BT-549, BCPAP, H1299, H460, HCT 116, HCT-8/5-FU, Hep G2, Huh-7, HOS, KYSE-150, MT-4, MCF-7, MCF-7/ADR, MDA-MB-231, MDA-MB-231/DOX, MG-63, PC-3, PC-9, SMMC-7721, SK-OV-3, SW480, SW620, TE-1, TE-8, TE-12, TPC-1, U-2 OS	143B xenograft mice, 231/PTX xenograft mice, 4T1 xenograft mice, A549 xenograft mice, Alb/JCPyV T antigen mice, B16-F10 xenograft mice, HCT 116 xenograft mice, HepG2 xenograft mice, KYSE-150 xenograft mice, LA795 xenograft mice, MCF-7/ADR xenograft mice, MDA-MB-231 xenograft, MC-38 xenograft mice, patient-derived tumor, zebrafish	Improve tumor immunity; induce cell cycle arrest, apoptosis; inhibit proliferation, migration; regulate autophagy, ferroptosis; reverse tumor drug resistance	Activate Caspase-3, Hippo pathway, ROS, SP1/Caveolin-1; Down-regulate Bcl-2, CDK2, CDK4, cyclin D, cyclin E, CXCL12, FN1, ING5; inhibit AKT/mTOR pathway, EGFR/JAK2/STAT3, mTOR phosphorylation, NRF2, STAT5 phosphorylation; up-regulate Bax, p21, p53	2 μM, 10 μM, 16 μM, 20 μM, 0–6 μM, 0–16 μM, 0–20 μM, 0–35 μM, 0–40 μM, 0–50 μM, 0–100 μM, 100–1000 μM, 0–40 μg/mL, 2.5–250 μg/ml; 10 mg/kg, 20 mg/kg, 25 mg/kg, 45.7 mg/kg, 50 mg/kg, 100 mg/kg, 200 mg/kg	Adriamycin, Cisplatin, Doxorubicin, Olanolic acid, Paclitaxel, Sorafenib	[1043, 1044, 1046–1052, 1056–1058, 1062–1065, 1067–1072, 1086, 1142–1147]

### Apigenin reverses cancer drug resistance

Apigenin has demonstrated significant potential in overcoming drug resistance across multiple cancer types through diverse molecular mechanisms. In nasopharyngeal carcinoma (HONE1 and CNE2) cells, apigenin inhibits drug resistance by suppressing epidermal growth factor receptor (EGFR) signaling [51]. In head and neck squamous cell carcinoma (HNSCC), apigenin reverses resistance to cetuximab by inhibiting extracellular signal-regulated kinase 1/2 (ERK1/2) and blocking RAS-Mitogen-activated protein kinase (MAPK) pathway [52]. Apigenin’s role in modulating drug resistance is further supported by its interaction with key pathways such as GLUT-1 and PI3K/AKT. Overexpression of GLUT-1, a marker for hypoxia, and the hyperphosphorylation of AKT are critical contributors to cisplatin resistance in laryngeal cancer cells, including HEp-2 cells. Apigenin is hypothesized to enhance cisplatin sensitivity in these cells by down-regulating GLUT-1 protein expression and AKT phosphorylation [53–55]. In ovarian cancer (SK-OV-3 and SK-OV-3/DDP) cells, apigenin reduces myeloid cell leukemia-1 (Mcl-1) mRNA and protein levels, a member of the anti-apoptotic Bcl-2 family, thereby reversing cisplatin resistance [32, 56, 57]. In doxorubicin-resistant liver cancer (BEL-7402/ADM) cells, apigenin restores drug sensitivity by reducing nuclear factor erythroid 2-related factor 2 (NRF2) expression at both RNA and protein levels through PI3K/AKT pathway inhibition, thereby increasing intracellular doxorubicin [58, 59]. In doxorubicin-resistant human uterine sarcoma (MES-SA/Dx5), apigenin reduces intracellular glutathione (GSH) levels by inhibiting ATP-binding cassette subfamily B member 1 (ABCB1) [60], while in prostate cancer, it reverses doxorubicin resistance by down-regulating ABCB1 protein expression [61]. Additionally, apigenin re-sensitizes Adriamycin-resistant breast cancer (MCF-7/ADR) cells by inhibiting the signal transducer and activator of transcription 3 (STAT3) pathway [62]. And in hypoxic tumor models, apigenin overcomes paclitaxel resistance by suppressing AKT and heat shock protein 90 (HSP90) signaling, which hinders the production of hypoxia-inducible factor-1*α* (HIF-1*α*) [63]. These findings highlight apigenin’s multifaceted role in overcoming drug resistance through interactions with drug efflux transporters and key signaling pathways.

### Apigenin suppresses cancer growth by regulating the processes of autophagy and ferroptosis

Autophagy is a cellular process that facilitates the transport of cytoplasmic macromolecules and organelles to lysosomes, where they undergo degradation and subsequent recycling [64, 65]. It has been demonstrated that the autophagy process is closely linked to a large number of proteins expressed by autophagy-related genes (ATGs). At the same time, the process of autophagy is also subject to regulation by the mTOR. For instance, the activation of its complex, mammalian target of rapamycin complex 1 (mTORC1), can impede the autophagic process [66]. Apigenin exhibits inhibitory effects on mTOR and p70S6K, leading to increased LC3-II conversion and GFP-LC3 puncta formation, hallmarks of autophage induction. Additionally, apigenin suppresses the mTOR/PI3K/AKT signaling pathway, thereby promoting autophagy in erythroid subtype leukemia (TF-1), liver cancer (Hep G2), and colon cancer (HT-29) cells [67–70]. Under hypoxic conditions, apigenin treatment in gastric cancer (AGS and SNU-638) cells enhances phosphorylation of ATG5, LC3-II, AMP-activated protein kinase (AMPK), and unc-51 like autophagy activating kinase 1 (ULK1), while simultaneously down-regulating p62, collectively driving autophagy-mediated cell death [71]. The process of ferroptosis is characterized by iron-dependent lipid reactive oxygen species (ROS) accumulation [72], represents another mechanism by which apigenin exerts its anti-cancer effects. Apigenin induces ferroptosis in multiple myeloma (NCI-H929) cells [73]. It mitigates myeloperoxidase (MPO)-mediated oxidative stress and inhibits ferroptotic cell death in neurons, while also enhancing glutathione peroxidase 4 (GPX4) activity [74, 75]. Thus, apigenin suppresses cancer growth through its regulation of autophagy and ferroptosis. By inhibiting the mTOR signaling pathway, apigenin promotes autophagy-mediated cell death, thereby limiting cancer cell survival under metabolic or oxidative stress. Additionally, its ability to induce ferroptosis in cancer cells further amplifies its therapeutic potential as an anti-cancer agent. These findings underscore apigenin’s multifaceted role in targeting distinct cellular processes, positioning it as a promising candidate for cancer therapy.

### Apigenin suppresses cancer progression via classical modalities

Apigenin has been found to influence multiple phases of the cell cycle in a variety of cancers, including skin, liver, breast, and colon cancers, primarily by causing G0/G1 phase arrest, blocking the transition from G0/G1 to S phase, and disrupting progression through the G2/M phase, thereby inhibiting cell proliferation [76]. Specifically, in colon cancer (HCT 116), apigenin induces G0/G1 phase arrest cells by up-regulating *miR-215-5p*, which modulates the expression of E2F transcription factors, key regulators of cell cycle progression [77]. Similarly, in prostate cancer (22Rv1 and PC-3) cells, apigenin treatment is shown to induce cell cycle arrest at the G0/G1 phase in a dose-dependent manner [78]. In breast cancer (MDA-MB-468) cells, apigenin partially extends the duration of the S phase [79]. Apigenin also modulates apoptosis in cancer cells through multiple pro-apoptotic pathways, including the mitochondrial pathway, Caspase activation, and oxidative stress induction. In cervical cancer (HeLa) cells, apigenin induce apoptosis via two distinct pathways: one involves down-regulating Bcl-2 protein expression, while the other activates p53 to varying degrees, leading to the induction of p21, fatty acid synthase (Fas) expression and ultimately apoptosis [80]. In lung cancer (A549) cells, apigenin increases cytochrome C levels, which in turn inhibits mitochondrial function, thereby leading to Caspase-9 and Caspase-3 activation and subsequent apoptosis [81]. Moreover, apigenin also inhibits tumor by modulating EMT and related protein expression. In human liver cancer (BEL-7402) cells, apigenin decreases Snail1 and NF-*κ*B protein levels while influencing cancer cell EMT [82]. In human melanoma (A375) cells, apigenin down-regulates STAT3 target genes matrix metalloproteinase 2 (*MMP-2*) and *MMP-9*, thereby suppressing tumor growth and invasion [83]. Collectivelly, apigenin suppresses cancer progression through a multifaceted mechanisms that includes cell cycle regulation, apoptosis induction, and metastasis inhibition. Apigenin arrests cancer cells in the G2/M phase or the G0/G1 phase, thereby halting their proliferation. It also modulates critical proteins like Bcl-2 and p53 to induce apoptosis, promoting cell death in various cancer types. Additionally, apigenin inhibits metastasis by down-regulating proteins involved in EMT and tumor cell invasion, such as Snail1, MMP-2, and MMP-9.

### Investigating apigenin anti-cancer effects through drug delivery system

Despite its promising anti-cancer effects, apigenin’s clinical application is limited by its poor water solubility. To address this challenge, advanced DDS have been developed to enhance its bioavailability, stability, and therapeutic efficacy. Emulsions, such as W/O/W formulations (water-in-oil-in-water), has been shown to significantly improve the oral bioavailability of apigenin [84, 85]. Moreover, the development of liposomal delivery systems presents a promising approach for the efficient administration of apigenin. The cytotoxicity of apigenin nanoliposomes in colon cancer (HCT-15 and HT-29) cells is stronger than that of free apigenin [86]. Apigenin and tocopherol derivative-containing D-alpha-tocopheryl polyethylene glycol 1000 succinate (TPGS) liposomes shows inhibitory effects in A549 tumor-bearing nude mice [87]. This combination leverages the unique properties of TPGS, which not only enhances the solubility and stability of apigenin but also prolongs its circulation half-life, thereby improving its bioavailability and therapeutic efficacy. The apigenin-loaded lipid nanocapsules (AP-LNC) are prepared using a phase transformation method, demonstrating a significant enhancement in the inhibition rate of Hep G2 and MCF-7 cells [88]. Poly(lactide-co-glycolide) (PLGA) nanoparticles (NPs) have been extensively utilized as polymeric carriers for DDS due to their biocompatibility, biodegradability, and ability to enhance drug solubility and stability. In melanoma (A375) cells, PLGA NPs prepared via the solvent replacement method exhibit a concentration-dependent inhibitory effect on tumor growth [89]. Similarly, PLGA NPs prepared through nanoprecipitation have been shown to effectively block p53 production in ovarian cancer (OVCAR4) cells, thereby exerting a more potent anti-tumor effect compared to free apigenin [90]. Additionally, multi-emulsion solvent evaporation technique has been use to create nanoparticles that effectively maintain hepatocyte structure and restore normal cellular function in Hep G2 cells [91].

In summary, apigenin exerts multi-target anti-cancer effects by modulating immune components, reversing multidrug resistance, regulating autophagy and ferroptosis, and inducing cell cycle arrest and apoptosis. Its poor solubility, however, necessitates the development of advanced drug delivery systems, such as liposomes and nanoparticles, which enhance its therapeutic potential across various cancer types. These systems improve solubility, control release, and target specific cancer types more effectively, positioning apigenin as a promising candidate for cancer therapy.

### Artemisinin

Artemisinin is a sesquiterpene lactone compound derived from *Artemisia annua* L., a traditional Chinese medicinal herb (Qinghao in Chinese) [92, 93]. Genetic analysis has demonstrated that amorpha-4,11-diene synthase (ADS), encoded by the ADS gene, functions as the first committed enzyme and plays a critical role in the artemisinin biosynthesis pathway in Artemisiae annuae [94]. Artemisinin has been demonstrated to possess multiple pharmacological activities, such as antimalarial, antiviral, antibacterial, anti-inflammatory, and anti-cancer properties [95–98]. To assess its safety profile, a machine learning-based toxicity prediction platform was developed, integrating in in vitro cardiotoxicity screening using cardiomyocyte cell lines and in vivo validation in zebrafish models. This approach accurately predicts the cardiotoxicity and other adverse effects of artemisinin derivatives, highlighting its utility in preclinical drug safety evaluation in cancer therapy [99, 100]. Moreover, efficacy study has demonstrated that artemisinin suppresses the proliferation and growth of diverse cancers, such as cervical, lung, prostate, bladder, ovarian, and breast cancers [101–105]. Its anti-cancer mechanisms are multifactorial, involving immunomodulation, induction of autophagy and ferroptosis, and suppression of tumor metastasis and angiogenesis [106, 107]. Furthermore, artemisinin synergizes with conventional chemotherapeutic agents, selectively targeting cancer cells while enhancing treatment efficacy [108–111]. Clinically, the therapeutic potential of artemisinin derivatives is under investigation in several trials: artesunate in the treatment of cervical intraepithelial neoplasia (NCT04098744), metastatic breast cancer (NCT00764036), and stage II/III colon cancer (NCT02633098). The following section reviews the pharmacological effects, intricate mechanisms, and specific biological targets of artemisinin across cancer type.

### Artemisinin regulates tumor immunity

Artemisinin modulates the immune response in liver, breast, colon, and leukemia cancers through enhancing the activity of NK and T cell function and decreasing regulatory T (Tregs) and MDSCs [112–114]. It attenuates lymphoma (K-562, Raji and YAC-1) cells by enhancing NK cell activity with low adverse effects [113]. Artemisinin suppresses tumor growth by enhancing the immunosuppression of Tregs and MDSCs, effectively reversing the immunosuppressive tumor microenvironment, inducing robust immunogenic cell death, and boosting anti-cancer immunity in 4T1 cells in vitro and in vivo [115]. Therefore, artemisinin holds significant promise as an immune-modulating agent in cancer therapy. It enhances the function of NK and T cells while simultaneously reducing immunosuppressive cells, such as Tregs and MDSCs. These dual effects reverse the immunosuppressive tumor microenvironment, thereby facilitating enhanced immune surveillance and a more robust antitumor immune response. Additionally, artemisinin induces immunogenic cell death, which not only directly eliminates cancer cells but also stimulates the body's inherent anti-cancer immune response, further amplifying its therapeutic effects.

### Artemisinin reverses multidrug resistance

Research suggests that artemisinin and its derivatives can modulate the activity of P-glycoprotein (P-gp), plasmodium falciparum multidrug resistance protein 1 (PfMDR1), and breast cancer resistance protein (BCRP) in breast, liver, colon and leukemia cancers [116, 117]. In the context of liver cancer, artemisinin has been demonstrated to reduce the protein expression of actin filament associated protein 1-like 2 (AFAP1L2) and reverses sorafenib resistance in specific liver cancer cell lines (Hep G2, Hep3B, MHCC-97 H, and Huh-7) [118]. Furthermore, artemisinin has been shown impair lysosomal function in lung cancer (A549/TAX) and breast cancer (MCF-7/ADR) cells, while subsequently improves the sensitivity of cancer cells to paclitaxel [119]. In addition, artemisinin, through mechanisms such as increasing drug loading, enabling rapid reactive release, and optimizing drug delivery sequence, can efficiently and precisely address the multidrug resistance in breast cancer (MCF-7 and LCC9) cells [117, 120]. Moreover, dihydroartemisinin has been found to overcome dexamethasone and oxaliplatin resistance in multiple myeloma (ARP1 and H929) and colon cancer (HCT 116 and SW620) cells by enhancing the sensitivity of these cells to dexamethasone and oxaliplatin [121, 122]. Furthermore, dihydroartemisinin damages heme metabolism in osimertinib-resistant EGFR mutant NSCLC (PC9-OR3, PC9-OR5, PC9-GR1-AZD2, and PC9-GR4-AZD1) cells, thereby inhibits cell proliferation with relatively low toxicity [123]. Thus, artemisinin and its derivatives exhibit a broad spectrum of activities against multidrug resistance in cancer treatment. They operate through multiple mechanisms, including modulating drug efflux proteins, reversing specific drug resistances, impairing lysosomal functions, optimizing drug delivery, and enhancing the sensitivity of cancer cells to chemotherapy agents. These multifaceted mechanisms position artemisinin as a promising candidate for incorporation into combination therapy regimens designed to overcome drug resistance in diverse cancer types.

### Artemisinin suppresses cancer growth by regulating the processes of autophagy and ferroptosis

Artemisinin and its derivatives exert its anti-cancer effects through the regulation of autophagy and ferroptosis via multiple distinct pathways. These pathways include mitochondrial autophagy, iron homeostasis regulation, and lipid peroxidation induction and ROS production. Such pathways have demonstrated to possess significant anti-tumor effects across a diverse range of cancer types, including liver, bladder, breast, endometrial, myeloma, and acute myeloid leukemia cancers [124]. Artemisinin regulates autophagy markers (LC3B and p62) to stimulate autophagy [125]. Artesunate induces ATG5 associated autophagy in bladder cancer (T24 and EJ) and endometrial cancer (Ishikawa and AN3 CA) cells [103, 126]. Moreover, artesunate regulates autophagy and ferroptosis by inhibiting STAT3 activation in non‑Hodgkin lymphoma (U2932, SU-DHL4, and Jurkat) cells [127]. Dihydroartemisinin has been found to inhibit cell proliferation in liver cancer (HepG2.2.15) cells in a dose- and time-dependent manner through the induction of autophagy [128]. Additionally, artemisinin plays a role in regulating the cancer progression, particularly in liver cancer, by inducing ferroptosis [129, 130]. Artesunate suppresses myeloma (MM1S) cell progression through inducing ferroptosis [131]. In lymphoma (U2932 and SU-DHL-4) cells, artesunate promotes ferroptosis by impairing the STAT3 pathway [132]. In primary liver cancer, dihydroartemisinin increases the level of lipid ROS and malondialdehyde, while simultaneously decreasing the protein expression of GPX4. These alterations trigger ferroptosis in primary liver cancer (Hep G2 and Hep3B) cells [133, 134]. Dihydroartemisinin also regulates iron metabolism to induce ferritin autophagy and subsequent ferroptosis in acute myeloid leukemia (MOLM-14 and OCI/AML-2) cells [135]. Furthermore, dihydroartemisinin has been demonstrated to increase the unstable iron pool, promote ROS accumulation, induce ferroptosis, and inhibit the proliferation of leukemia (HL-60, KG-1, and THP-1) cells [136]. Novel 1,3, 4-oxadiazol-artemisinin hybrids has been found to induce ferroptosis in breast cancer (MCF-7) cells in a dose-dependent manner [137]. Briefly, artemisinin and its derivatives exert anti-tumor effects on a variety of cancer cells through multiple mechanisms. These mechanisms involve reducing protein expression, promoting mitochondrial autophagy, inducing apoptosis, enhancing cytotoxicity, activating specific pathways, regulating iron metabolism and ROS production, and ultimately inducing autophagy, apoptosis, and ferroptosis. Moreover, these effects can lead to an improvement in the sensitivity of cancer cells to chemotherapy drugs.

### Artemisinin and its derivatives suppress cancer progression via classical modalities

Artemisinin and its derivatives have been demonstrated to exert inhibitory effects on cancer progression through multiple well-established mechanisms. For instance, artesunate inhibits colon cancer cell proliferation by inducing cell cycle arrest at G0/G1 phase and subsequent cell senescence [138]. Artemisinin down-regulates the mRNA levels of proliferation-related genes (*c-Myc*, *N-cadherin*, *Vimentin*, *Snail*, *E-cadherin*) in renal cancer (UMRC-2 and Caki-2) cells to attenuate cancer progression [139]. Dihydroartemisinin inhibits the growth and invasion of sgastric cancer (SGC-7901, MKN-28 and MGC-803) cells through the modulation of key signaling pathways such as the Cyclin D1-CDK4-Rb and STAT1/KDR/MMP-9 pathways [140–142]. Moreover, artemisinin modulates the expression of apoptotic proteins in a dose-dependent manner in salivary gland cancer (A-253/HTB-41) cells. Specifically, it increases the pro-apoptotic protein (Bax, Bim, Bad and Bak) expression, while decreasing anti-apoptotic protein (Bcl-2 and Bcl-xL) expression, and activates Poly (ADP-ribose) polymerase 1 (PARP1) and Caspase-3, ultimately triggering cell death [143]. Dihydroartemisinin induces apoptosis of breast cancer (4T1) cells by enhancing ROS release, increasing p53 protein expression and activating Caspase-3 [144]. Artesunate significantly inhibits the growth of lung cancer (A549) cells by interfering with cell cycle progression, reducing the protein expression of Bcl-2 and MMP-9, and increasing the protein expression of Bax [145, 146]. In ovarian cancer (IOSE144, A2780, OVCAR3, and SK-OV-3) cells, dihydroartemisinin downregulates Bcl-2 and upregulates Bax, which inhibits cancer cell proliferation and leads to cell cycle arrest at the S phase [104, 147, 148]. To sum up, artemisinin and its derivatives inhibit the proliferation, migration, and invasion of various cancer cells through multiple distinct mechanisms. They also exhibit diverse effects such as anti-inflammatory activity, enhanced cell cycle arrest, and induction of apoptosis in specific cancer types. Furthermore, they possess broad anti-tumor potential, as evidenced by their ability to inhibit angiogenesis in relevant animal models.

### Artemisinin and its derivatives combined with other medications for cancer treatment

The combination of artemisinin or its derivatives with different chemotherapeutic agents (e.g., Oxaliplatin, carboplatin) and other natural compounds (e.g., Resveratrol) has been demonstrated to yield synergistic anti-tumor effects. For example, when artemisinin is combined with oxaliplatin, the anti-tumor effect of oxaliplatin on esophageal cancer (EC109) cells is enhanced [149]. Artesunate and carboplatin, when used in combination, promote cell cycle arrest and induce the expression of cell cycle and apoptosis-related proteins, thereby exerting synergistic anti-tumor effects on NSCLC (A549) cells [150]. When artemisinin and resveratrol are encapsulated into nano-sized liposomes, HT-29 cell growth is inhibited in a time- and dose-dependent manner [151]. Additionally, combination studies have revealed that trastuzumab inhibits breast cancer (HCC1569, HCC1954, and BT-474) cell proliferation more effectively when combined with dihydroartemisinin compared to single drug treatment [152]. Dihydroartemisinin plays a significant anti-tumor role in multiple cancers and has been shown to enhance the anti-tumor efficacy of cisplatin in NSCLC (A549) cells [153]. Dihydroartemisinin and capecitabine have a synergistic effect on human colon cancer (HCT 116, DLD-1, SW620, and HCT-15) cells and mitigate the side effects induced by capecitabine [154]. Dihydroartemisinin has been demonstrated to suppress the proliferation, migratory abilities, and invasive capacity of various breast cancer (MCF-7, MDA-MB-231, and BT-549) cells [155]. The combination of dihydroartemisinin and resveratrol synergistically inhibits the migration of liver cancer (Hep G2) and breast cancer (MDA-MB-231) cells [156]. The 1:1 combination of sulfampyridine and dihydroartemisinin exhibits high efficacy against glioma (TN22) cells [157]. Artemisinin, in conjunction with sorafenib and venettok, has been shown to display a collaborative inhibitory action on the proliferation and trigger cell death in diverse acute leukemia cell lines under laboratory conditions [158]. The combination of dihydroartemisinin and cisplatin synergistically inhibits the proliferation of pancreatic ductal adenocarcinoma (PANC-1 and SW1990) cells and induces ferroptosis by modulating iron metabolism [159]. Additionally, when dihydroartemisinin and sorafenib are combined, they can inhibit Hep G2 cell proliferation and induce ferroptosis. This combination generates high levels of ROS, promoting cancer cell apoptosis and demonstrating synergistic anti-cancer effects in hepatoma (Hep G2) cells [160, 161]. The combination of artesunate and sorafenib shows synergistic antitumor activity in hepatocellular cancer (Hep G2) cells [162]. Overall, the combination of artemisinin with different chemotherapy agents and other natural compounds demonstrates significant anti-tumor effects, including enhancing the anti-proliferation, pro-apoptotic, and cell cycle regulation effects of oxaliplatin, metformin, and carboplatin on various cancer cell lines. Such combination treatments hold great potential in both clinical and experimental settings, as they enhance anti-tumor activity and exhibit synergistic effects.

### Investigating artemisinin anti-cancer effects through drug delivery system

To enhance the bioavailability and enable more specific targeting of artemisinin for tumors treatment, researchers have been actively exploring diverse drug delivery systems. These include nanoparticles, liposomes, polymer micelles, dendrimers, hydrogels. Such system can improve the efficacy of artemisinin and present a promising strategy in the treatment of lung, colon, and breast cancers [163–165]. For instance, when artemisinin is combined with metformin within co-carrier nanoparticles, it exhibits superior anti-proliferative effects on lung cancer (A549) and breast cancer (T-47D) cells compared to its free form [163, 166]. Additionally, artemisinin loaded into manganese oxide within a co-delivery system has been shown to possess the ability to penetrate deeply into solid tumors, enabling precise diagnostic and targeting capabilities, especially for breast cancer (T-47D) cells [167]. The oral administration system using heme lipid artemisinin nanoparticles demonstrates remarkable stability with the gastrointestinal tract and during blood circulation. However, these nanoparticles can dissociate in the tumor microenvironment, which significantly augments their accumulation within tumors. In mouse models, this property has led to the complete suppression of colon cancer (MC38) cells within 30 days [168]. Copper artemisinin nanoparticles can achieve cascade amplification of ROS production and have a high tumor inhibition rate in breast cancer (4T1) xenograft model [169]. The use of tween 80 micelles for artemisinin-loaded micelles effectively prevents premature drug release during circulation, and this system has shown a significant tumor inhibitory effect, with inhibition rates reaching up to 85% [170]. Biodegradable dihydroartemisinin nanoparticles, employing a MPEG-PCL micelle carrier, ensure a sustained release of the drug in aqueous solutions. These nanoparticles have shown to be more effective and less toxic in vivo compared to free dihydroartemisinin [171]. To sum up, the development of novel drug delivery systems has significantly enhanced the bioavailability, stability, and targeting efficiency of artemisinin, thereby improving its potential as an anti-cancer agent. Systems such as nanoparticles, liposomes, and micelles play crucial roles in ensuring that artemisinin is delivered effectively to tumors, which in turn enhances its therapeutic effects while minimizing potential side effects. Approaches like co-carrier nanoparticles, manganese oxide-based co-delivery, copper-based ROS amplification, and biodegradable nanoparticles have all yielded promising results in preclinical models of lung, colon, and breast cancers. These advancements suggest that artemisinin, when delivered through optimized drug delivery systems, holds great promise as a more effective treatment for a variety of cancers.

### Artemisinin derivatives inhibit cancer progression

Artemisinin derivatives have been shown to exert inhibitory effects on cancer progression through multiple mechanisms. Dihydroartemisinin has been demonstrated to modulate cytotoxic T lymphocyte (CTL) function by influencing Treg cell inhibition, thereby enhancing anti-cancer immunity in mice and inhibiting the growth of melanoma (B16-F10) cells [172, 173]. Artesunate effectively reduces the protein expression of PD-L1 in NSCLC (NCI-H1975, NCI-H460, A549, and PC-9) cells [174]. Dihydroartemisinin augments the responsiveness of colon cancer (HCT 116 and RKO) cells to oxaliplatin [175]. When artesunate and chloroquine are combined, they can regulate macrophages, inhibit the proliferation of colon cancer (HCT 116 and CT26) cells, and reverse the immunosuppressive tumor microenvironment [176]. The concurrent delivery of dihydroartemisinin and pyrophosphorus-iron induces increased production of ROS, thereby triggering ferroptosis. This process not only leads to cancer cell death but also enhances tumor immunogenicity, rendering previously non-immunogenic colon tumors susceptible to anti-PD-L1 checkpoint blockade immunotherapy [177]. Dihydroartemisinin promotes anti-programmed death-1 (PD-1) action in liver cancer (HepG2.2.15) cells [178]. The combination of oxaliplatin and dihydroartemisinin has a strong synergistic effect on ROS generation. The combination of ROS generation and immunostimulant using nanomedical drugs works synergistically with checkpoint blocking immunotherapy to treat colon cancer (CT26 and MC38) in mice [179]. In glioblastoma multiforme, the combination of artesunate with lower doses of metformin demonstrates antitumor effects by activating the AMPK/mTOR pathway and inducing autophagy in human glioblastoma (U251 and U118-MG) cells [180]. Artemisinin derivatives with piperazine and fluorophore suppresses the cell cycle at the G0/G1 phase, increases Bax expression, decreases Bcl-2 protein expression, induces concentration-dependent apoptosis, and inhibits cell migration via E-cadherin and N-cadherin pathways in colon cancer (HCT 116) cells [181]. Artemisinin and its derivatives inhibit the migration and invasion of cancer cells, and have anti-tumor and anti-angiogenic effects on tumor xenografts in nude mice [182–185]. Dihydroartemisinin influences colitis-associated colon cancer (CACC) at multiple stages. Artesunate and sorafenib synergically induce lipid peroxidation and ferroptosis in liver cancer (Hep G2, SNU-182 and SNU-449) cells [186]. Dihydroartemisinin also causes lipid peroxidation and ROS generation, which leads to ferroptosis in glioma (U251 and U373) cells [187]. Dihydroartemisinin@MIL-101 nanoreactor produces ROS and consumes GSH, resulting in the accumulation of lipid peroxide and induction of ferroptosis in lung cancer (Lewis) cells [188]. Overall, artemisinin derivatives, including dihydroartemisinin and artesunate, exhibit a wide range of anti-cancer effects by modulating immune responses, enhancing chemotherapy efficacy, inducing ferroptosis, and inhibiting cancer cell proliferation, migration, and invasion across various cancer types.

To sum up, artemisinin and its derivatives exemplify the potential of a single phytochemical to modulate immunity, overcome drug resistance, and induce cell death pathways such as ferroptosis and autophagy. Continued investigations into synergistic regimens, targeted drug delivery, and clinical safety profiles are of utmost importance for fully realizing artemisinin’s therapeutic potential in cancer treatments.

### Berberine

Berberine is an isoquinoline alkaloid, mainly extracted from *Coptis chinensis* or Coptidis Rhizoma (Huanglian in Chinese). Berberine has been long-term used for its anti-inflammatory, antioxidant, anti-diabetic, cardioprotective, hepatoprotective, and neuroprotective effects [189, 190]. Regarded as a promising anti-tumor candidate, berberine has diverse pharmacological properties [191–193]. Berberine modulates cancer immunity, reduces drug resistance, promotes apoptosis and autophagy, inhibits cell cycle progression, and curtails invasion and metastasis in a range of cancers such as lung, breast, colon, liver, gastric, prostate, ovarian, and leukemia [194, 195]. In silico studies shows that berberine interacts with key oncogenic proteins such as p53, EGFR, AKT, p38, and ERK1/2, while in vitro experiments validated its ability to decrease the phosphorylation of these proteins, highlighting its potential as an effective treatment for oral squamous cell carcinoma and breast cancer [196, 197]. Ex vivo studies demonstrate that berberine and its eight analogs inhibit cancer cell viability in a canine mammary carcinoma cell line and transgenic zebrafish models through apoptosis induction, while zebrafish embryo toxicity experiments are conducted to assess the in vivo safety of berberine [198, 199]. In clinical studies, berberine has been tested in various cancers, including lung cancer (NCT03486496), colon cancer (NCT03281096), and gastric cancer (NCT03609892). However, the clinical application of berberine is restricted because of its poor water solubility and low bioavailability. To avoid these disadvantages, advanced delivery systems such as nanoparticles and liposomes are developed to improve the solubility and stability of berberine [200–202]. This section is dedicated to discussing the current investigations into the anti-tumor efficacy of berberine, both as a standalone and as part of combination therapies.

### Berberine improves tumor immunity

Berberine has indicated significant immunomodulatory actions in regulating tumor microenvironment by modulating various immune cells, inflammatory mediators, and immune checkpoint pathways [203]. For breast cancer, by modifying the inflammatory cytokine network and immune cell infiltration, berberine has been shown to inhibit tumor growth and metastasis in hypoxic environments [204, 205]. Berberine enhances the activation of NK cells, DCs, and CTLs, while reducing the populations of MDSCs and TAMs [206, 207]. Moreover, berberine suppresses the growth of liver cancer by reducing the number of MDSCs in tumor tissues and inhibiting angiogenesis [208]. Additionally, berberine protects liver cancer progression by modulating intrahepatic T cell heterogeneity, potentially improving the efficacy of cancer immunotherapy [194, 209]. Berberine has been shown to reprogram TAMs from M2 to M1 phenotype, thereby enhancing the anti-tumor immune response [210]. Regarding inflammatory mediators, berberine significantly reduces the levels of inflammatory cytokines, including tumor necrosis factor-alpha (TNF-*α*), interleukin-6 (IL-6), and IL-1*β*, and downregulates their protein expression [210]. In a breast cancer model, berberine inhibits tumor growth under hypoxic conditions by modulating the inflammatory cytokine network and immune cell infiltration [210, 211]. Berberine exerts antitumor effects by inhibiting PD-L1 activity, improving T-cell-mediated immunity. It is achieved by suppressing the deubiquitinating of CSN5 and leads to PD-L1 ubiquitination and degradation, reactivating cytotoxic T-cells and reducing immunosuppressive cells in the tumor microenvironment [210]. Therefore, berberine shows great promise as a multi-target agent capable of modulating the tumor microenvironment to promote anti-tumor immunity. Its ability to enhance immune cell function, reduce immunosuppressive cells, reprogram TAMs, decrease inflammatory mediators, and inhibit immune checkpoints, positions it as a valuable compound for cancer therapy.

### Berberine reverses tumor drug resistance

Berberine has been reported to reverse drug resistance in various cancer types by targeting multiple drug resistance mechanisms. One of the key mechanisms through which berberine combats multidrug resistance is its ability to inhibit drug efflux pumps, particularly in regulating the ABC transporter family, including P-gp and Multidrug resistance protein 1 (MRP1). Berberine has been shown to downregulate the expression of these transporters, thereby increasing the intracellular retention and potency of drugs like doxorubicin. Experimental evidence indicates that berberine, combined with doxorubicin, significantly reduces the efflux of chemotherapeutics, and enhances their cytotoxic effects in breast cancer [212, 213]. Additionally, berberine can reverse drug resistance by activating AMPK, which is involved in cellular energy homeostasis. It is a potential therapeutic target in drug-resistant cancers. Berberine can cause a change in the metabolism of cancer cells by activating AMPK. It can reverse the Warburg effect. This metabolic reprogramming disrupts the energy supply of drug-resistant cancer cells. It leads to increased sensitivity to chemotherapy. Moreover, the activation AMPK can downregulate HIF-1*α*, which is a hypoxia-inducible factor that promotes P-gp expression, and further enhances chemosensitivity. Studies also demonstrated that berberine sensitizes drug-resistant breast cancer cells to doxorubicin by the modulation of the AMPK-HIF-1*α*-P-gp pathway [214]. Berberine can also target mitochondria, which are critical organelles in energy production and apoptosis regulation. Due to its delocalized positive charge, berberine can disrupt mitochondrial membrane potential and upregulate the content of ROS. These mitochondrial perturbations can stimulate intrinsic apoptosis pathways. It can effectively result in cell death in drug-resistant cancer cells. Furthermore, mitochondrial targeting by berberine is increased if combined with nanomedicine platforms, such as GSH-responsive paclitaxel-berberine conjugates, which can achieve simultaneous drug release and targeted delivery. This approach has shown superior efficacy in reversing resistance in lung and breast cancer models [215]. For the synergistic effects with chemotherapeutics, berberine can increase the efficacy of conventional chemotherapeutic agents. It has been indicated across various cancer types, including breast, liver, lung, and colon cancers. Its synergistic effects are attributed to multiple mechanisms, including enhanced drug uptake, efflux transporters inhibition, and cancer cell metabolism disruption. For instance, berberine can improve doxorubicin uptake in tumor tissues while simultaneously down-regulate the protein expression of P-gp and MRP1 in breast cancer. This dual action not only improves chemotherapeutic efficacy but also reduces the likelihood of multidrug resistance development [212]. Berberine can reverse the multidrug resistance of breast cancer via the suppression of the efflux function of ABC transporters. In vivo experiments have shown that berberine can treat Adriamycin-resistant breast cancer patient as well [216]. Therefore, berberine shows promise as a treatment for tumors that are resistant to various drugs. Upon targeting efflux transporters, reprogramming cancer metabolism, inducing mitochondrial apoptosis, and synergizing with chemotherapeutic agents, berberine exhibits the multi-faceted nature of drug resistance. The integration of innovative delivery systems further augments its potential. It paves the way for its translation from bench to bedside. Continued research and clinical trials are recommended to fully identify the therapeutic potential of berberine.

### Berberine suppresses cancer growth by regulating the processes of autophagy and ferroptosis

Berberine could apoptosis and autophagy in cancer cells through various mechanisms. Berberine can also act as an autophagy regulator to regulate physiological and pathological conditions and reduce drug resistance in cancer treatment [217]. Regarding apoptosis, berberine primarily upregulates pro-apoptotic proteins while down-regulating anti-apoptotic proteins. In liver cancer cells, berberine induces apoptosis by activating Caspase-3 and Caspase-9 through the *miR-221*/ SRY-related HMG-box 11 (SOX11) axis [218]. Similarly, in renal cell carcinoma, berberine treatment increases ROS levels and apoptosis rates, accompanied by the protein upregulation of Bax, Bad, Bak, Cytochrome, the downregulation of Bcl-2c and activating Caspase-3, and Caspase-9 [219]. Berberine can significantly suppress the proliferation of human glioma (U-87 MG) cells and induce the apoptosis of U-87 MG and LN-229 cells by down-regulating the protein expression of Bcl-2, up-regulating that of Bax and activating Caspase-3 [220]. In a study on the anti-cancer effect of berberine on Peripheral blood mononuclear cells (PBMCs) from patients with chronic lymphocytic leukemia, berberine is found to promote apoptosis by reducing Bcl-2, ROR1, and *mir-21* gene levels [221]. Berberine suppresses EMT and promotes apoptosis of colonic epithelial cells induced by tumor-associated fibroblasts by regulating TGF-*β* signaling [222]. Self-assembled mitochondria-targeted nano drugs (HA/PEG/BD NDs) from 9-O-octadecyl-substituted berberine derivatives exhibit significant mitochondrial targeting of tumor cells and remarkable anti-tumor efficacy in A549 genetically engineered tumor models. HA/PEG/BD NDs induce apoptosis through dissipation of the mitochondrial membrane potential, release of cytochrome C, and increase in Caspase-9/3 activity, activation of pro-apoptotic Bax, inhibition of anti-apoptotic Bcl-2 and up-regulation of ROS levels to induce apoptosis [223]. Based on these reports, the anti-cancer properties of berberine can be regarded by stimulating both endogenous and exogenous apoptosis via increasing the protein expression of pro-apoptotic molecules Bax, Bad, Bak, and activating Caspase-3, Caspase-8 and Caspase-9. Berberine also induces autophagy in cancer cells, although the effects appear to be context-dependent and involve multiple signaling pathways. In melanoma cells, berberine induces autophagic cell death by inactivating the AKT/mTOR signaling pathway [224]. Similarly, in acute lymphoblastic leukemia cells, berberine induces autophagy and cell death by inhibiting the AKT/mTORC1 signaling pathway [225]. In gastric cancer cells, berberine induces autophagy inhibition through the regulation of MAPK/mTOR/p70S6K and AKT pathways, leading to the suppression of cancer cell growth both in vitro and in vivo [226]. Berberine has been reported to act as a naturally occurring MET inhibitor that synergizes with ositinib to overcome acquired resistance to ositinib caused by MET amplification [227]. Berberine synergistically and selectively reduced the survival of several MET-amplified Ositinib-resistant EGFR-mutant NSCLC cells when used in combination with Ositinib. It may enhance the induction of apoptosis through Bim elevation and Mcl-1 reduction [228]. Therefore, berberine's broad spectrum of action against cancer cells through the regulation of autophagy and ferroptosis highlights its potential as a powerful anticancer agent. Its ability to target multiple cellular mechanisms responsible for cancer progression and survival offers a promising avenue for developing more effective cancer treatments.

### Berberine suppress cancer progression via classical modalities

Berberine can block the progression of the cell cycle at the G0/G1 phase, preventing the transition to the S phase, thereby inhibiting cancer cell proliferation. Additionally, it can interfere with the S phase and G2/M phase, disrupting DNA synthesis and mitosis. In breast cancer cells, berberine and its derivatives arrested the G2/M phase by inhibiting HIF-1*α* activity and regulating its downstream targets through non-coding RNAs [205, 229]. Moreover, berberine induced S phase arrest in breast cancer (MDA-MB-231) cells. It enhances their chemosensitivity to conventional therapies [230, 231]. The molecular mechanisms underlying berberine-induced cell cycle arrest include the increase of cyclin-dependent kinase (CDKs) inhibitors, such as p21 and p27, and the decrease of Cyclin D1 [215]. Interestingly, berberine has been found to promote the ubiquitination of insulin-like growth factor 2 mRNA-binding protein 3 (IGF2BP3) through the E3 ubiquitin ligase TRIM21. It leads to S phase arrest in colon cancer cells [232–234]. Berberine can arrest the cell cycle of lymphocytic Jurkat cells at G0/G1 phase, suggesting that it may play a positive role in the treatment of leukemia [235]. Studies have shown that for Tca8113 and MCF-7, the maximum arrest occurred after 12 h of berberine treatment, and for Hela, CNE2 and HT-29, the maximum arrest was observed at 24, 36 and 24 h, respectively. The results showed that Berberine could induce different cancer cells to arrest at G2/M phase [236]. Mitochondria-targeted nanomedicines self-assembled from GSH-responsive paclitaxel-SS-berberine conjugates can upregulate ROS levels in cancer cells, arrest cells in the G2/M phase, induce cancer cell apoptosis, and inhibit tumor growth [237]. Berberine could suppress bladder cancer (BIU-87 and T24) cells in a dose- and time-dependent manner. It can promote cell cycle arrest at G0/G1 site, and induce cell apoptosis in a dose-dependent manner [231].

Recent studies have revealed that berberine can regulate cell cycle depending on the cancer type. In bladder cancer (T24 and 5637) cells, berberine inhibited cell proliferation and cell cycle progression by down-regulating the HER2/PI3K/AKT signaling pathway [238]. Berberine inhibits cyclin activity by regulating PI3K/AKT, MAPK, and Wnt signaling pathways at the colon prelesion stage, thereby slowing down the cell cycle progression of polyp or adenoma cells [239]. In addition, berberine affected the expression of several proteins important in tumor cell cycle progression, These proteins include Cyclin-D1, Cyclin-B1, Cdc25, CDK1, Epidermal Growth Factor (EGF), Raf/MEK/ERK, and Platelet-derived growth factor (PDGF), activated enhancer-binding protein-1 (AP-1), AP-2, and CD147 [240]. Thus, berberine could arrest the cell cycle at different stages, thereby inhibiting the growth and proliferation of cancer cells, and is a promising potential anti-cancer drug.

Berberine has demonstrated significant anti-invasive and anti-metastatic effects in various cancer types by modulating multiple signaling pathways. In glioma cells, berberine suppresses cell proliferation, migration, and invasion by inhibiting the TGF-*β*1/SMAD2/3 signaling pathway [220]. Similarly, in osteosarcoma (MG-63 and U-2 OS) cells, berberine inhibits tumor cell migration and invasion by regulating the MMP/NM-23 and MAPK/ c-Jun N-terminal kinase (JNK) signaling pathways [241]. Advanced drug delivery systems have further enhanced the anti-metastatic potential of berberine. In a breast cancer xenograft model (MDA-MB-231), mitochondria-targeted nanomedicines co-assembled from berberine and doxorubicin effectively repairs mitochondrial protein defects (Mfn1/Drp1) and inhibits the activity of MMP-2 and MMP-9, resulting in significant suppression of tumor cell proliferation and lung metastasis [242]. Moreover, berberine can inhibit glioma cell migration and invasion by suppressing the TGF-*β*1/COL11A1 activity and regulating the actin cytoskeleton arrangement [243]. The anti-metastatic effects of berberine are mediated through the regulation of multiple signaling cascades, including MAPK, PI3K-AKT, and NF-*κ*B pathways [205, 220, 229, 231, 238, 241, 244, 245]. Therefore, berberine demonstrates remarkable anti-invasive and anti-metastatic properties by modulating multiple signaling pathways. It can effectively inhibit tumor cell migration and invasion by targeting MMPs, regulating cytoskeletal dynamics, and repairing mitochondrial protein defects. Furthermore, advanced delivery systems, such as mitochondria-targeted nanomedicines, further improve their therapeutic potential for combating cancer progression.

### Synergistic effects of berberine with other drugs

Recent studies have highlighted the potential of combining berberine with conventional cancer therapies to enhance treatment efficacy and reduce side effects [246]. Berberine-loaded nanoparticles, such as chitosan/pectin nanoparticles, have shown enhanced cytotoxicity against gastric cancer cells by modulating epigenetic factors (*miR-185-5p* and *KLF7*) [247]. Berberine, combined with cisplatin, has a synergistic anti-cancer effect on the MG-63 osteosarcoma cell line. It can significantly promote osteosarcoma cell death by the inhibition of the protein expression of MMP-2, Bcl-2, Cyclin D1, and CDK4, and up-regulating Bax expression [248]. Berberine is a regulator of the HMGB1- Toll-like receptor 4 (TLR4) axis and can inhibit breast cancer metastasis aggravated by chemotherapy. Doxorubicin and berberine can be assembled into nanomedicines without the help of additional carriers. This nanomedicine not only effectively inhibits tumor growth with fewer side effects, but also significantly inhibits lung metastasis by blocking the HMGB1-TLR4 axis [249]. Janus nanocarrier for doxorubicin and berberine (HA-MSN@DB) can be delivered to liver cancer cells via CD44 receptor-mediated targeting effect. Berberine significantly reduces adriamycin-triggered repopulation of liver cancer cells in vitro and in vivo by inhibiting the Caspase-3-iPLA2-COX-2 pathway. Thus HA-MSN@DB co-delivery attenuates chemotherapy-exacerbated liver cancer recurrence [250]. Moreover, berberine has also been reported to improve the sensitivity of cancer cells to radiotherapy. In ovarian cancer (SK-OV-3) cells, berberine significantly increases the radiosensitizing effect, potentially improving the outcomes of radiotherapy [251]. Therefore, berberine demonstrates significant potential as an adjunct to conventional cancer therapies, including enhancing treatment efficacy, overcoming drug resistance, and reducing side effects. Berberine has been identified as a flexible and attractive candidate for enhancing the results of cancer treatment across a range of cancer types due to its capacity to work in concert with chemotherapeutics, targeted treatments, and radiation therapy, when combined with sophisticated delivery systems.

### Application of multi-omics techniques to investigate the anti-cancer properties of berberine

The combination of omics technologies, such as transcriptomics, proteomics, metabolomics, and multi-omics methods, could greatly improve the understanding of multifaceted activity of berberine [252]. It may aid in the development of berberine-based treatments for various disorders.

For transcriptomics, high-throughput RNA sequencing is useful for determining the effect of berberine on gene expression patterns. Berberine has been demonstrated to change the expression of critical regulatory genes in the liver. It can result in hypolipidemic consequences. This modulation includes the activation of genes related to lipid catabolism and the downregulation of lipid synthesis-associated genes. It could lead to a lower risk of metabolic diseases. Furthermore, transcriptome investigations have demonstrated that berberine modulates the expression of genes associated with inflammation and glucose homeostasis. It highlights its therapeutic potential in metabolic illnesses [253]. For the proteomics, mass spectrometry-based proteomics has been critical in the determination of the protein targets and signaling cascades impacted by berberine treatment. Berberine can regulate the expression of proteins involved in mitochondrial activity and oxidative stress in colon cancer cells. Berberine has been shown to increase mitochondrial respiration and energy generation while decreasing oxidative stress and apoptosis. These proteome alterations aid in promoting cell death and inhibiting cancer cell proliferation. It may highlight berberine's potential as an anti-cancer medication [254]. For metabolomics, metabolomic profiling has shed information on the metabolic changes caused by Berberine treatment. Significant variations in bile acid composition have been detected. It suggests that berberine plays a role in modulating gut flora and metabolic health. Berberine affects the intestinal environment via changing bile acid synthesis and composition, promoting the growth of beneficial bacteria communities. This alteration of the gut microbiota is related with improved metabolic parameters, such as increased insulin sensitivity and lower inflammation, highlighting the relevance of the gut-liver axis in the therapeutic effects of berberine [255]. For the lipidomic, lipidomic investigations have shown that berberine changes lipid profiles. It contributes to the therapeutic benefits in metabolic diseases. Berberine affects cellular membrane composition and function by regulating the amounts of various lipid species such as cholesterol esters, phospholipids, and sphingolipids. These alterations can have an impact on signaling pathways related to lipid metabolism, inflammation, and insulin sensitivity, potentially improving disorders including hyperlipidemia and nonalcoholic fatty liver disease. Furthermore, lipidomic analysis has discovered lipid biomarkers that can be regarded as indications of berberine efficacy. It can help customize personalized treatment regimens [256]. For epigenomics, according to research, berberine effects epigenetic alterations such as DNA methylation and histone acetylation. It can influence gene expression patterns linked to cancer progression. Berberine can regulate the shape of chromatin and the accessibility of transcriptional apparatus to certain genes by regulating the activation of enzymes involved in various epigenetic pathways. It can inhibit cancer cell proliferation and spread. These findings indicate that berberine may function as an epigenetic modulator with potential uses in cancer therapy. According to research, berberine can affect epigenetic alterations such as DNA methylation and histone acetylation. It can influence gene expression patterns for cancer progression. Berberine can change chromatin structure via altering the activity of enzymes in these epigenetic processes [257]. For pharmacogenomics, scientists identified genetic variants that can regulate berberine metabolism, transport, and target interactions. This information allows for adjusting berberine dose regimens and the selection of appropriate patient demographics. It may improve therapeutic outcomes while reducing the likelihood of treatment failure [258]. For glycomics, scientists have provided insights into the role of berberine in modulating immune responses and inflammation. By altering the glycosylation patterns of immune signaling-related proteins, berberine can influence the recognition and response of immune cells to pathogens and damaged tissues. This modulation of glycan structures could affect processes such as cell adhesion, migration, and cytokine production. It can contribute to the anti-inflammatory and immunomodulatory effects of berberine [259]. For the toxicogenomic, assessments of gene expression alterations associated with toxicity could contribute to a better knowledge of the safety of berberine. Toxicogenomic studies can predict probable side effects and determine safety dose ranges by assessing the expression of genes involved in detoxification, oxidative stress response, and apoptosis. For chemogenomic techniques, which combine chemical and genomic data, can predict interactions between berberine and other proteins. It can explain its mode of action. For example, research has found that berberine interacts with enzymes involved in lipid metabolism and inflammatory processes. It explains the molecular foundation for its therapeutic benefits. Such information is crucial for the rational creation of berberine compounds with increased efficacy and fewer adverse effects [260]. Therefore, these integrative Omics-related approaches have not only advanced our understanding of berberine's therapeutic benefits, but also paved the path for personalized medicine tactics and the creation of innovative therapeutic agents.

Overall, berberine exemplifies how a single phytochemical can orchestrate complex anti-cancer effects, extending from reprogramming the tumor microenvironment and reversing multidrug resistance to reshaping metabolic and epigenetic landscapes. Future directions lie in harnessing multi-omics insights and advanced delivery platforms to optimize berberine’s translational potential, aiming to integrate it effectively into personalized cancer therapies. However, it is easy to produce false positive data due to the overlap between the emission wavelengths of berberine and those of most experimental fluorescent probes, so this requires special attention from researchers during future studies on berberine.

### Curcumin

Curcumin, a phenolic compound extracted from Curcumae longae rhizoma (Jianghuang in Chinese) and Curcumae rhizoma (Ezhu in Chinese) [261, 262], demonstrates a broad array of pharmacological activities. These include anti-inflammatory, antioxidant, antibacterial, antiviral, and antidepressant qualities, as well as cardioprotective, hepatoprotective, and neuroprotective benefits [263, 264]. Additionally, curcumin exhibits a wide range of anti-tumor properties, proving effective against various types of cancer, such as lung, breast, colon, bladder, head and neck, prostate, kidney, cervical, thyroid, pancreatic cancer cells, and even melanoma, non-Hodgkin lymphoma, and leukemia [265, 266]. In silico studies show that curcumin, when combined with chemotherapy drugs, exhibit strong interactions with target proteins, with curcumin achieving the highest interaction scores with marker proteins, and curcumin derivatives fitting well in the active site of target proteins, suggesting their potential as effective anticancer agents for breast cancer [267, 268]. It restrains cancer progression by modulating the host immunity, promoting autophagy and ferroptosis, eliciting apoptosis, and inhibiting invasion and metastasis. Curcumin affects cancer cells by modulating several immune mediators, including cytokines, ROS, and cyclooxygenase-2 (COX-2). It also contributes to diminishing the activity of oncogenic molecules, protein kinases, growth factors, and critical signaling pathways, including NF-*κ*B, JNK, and STAT3 [269]. Although curcumin holds promise as an anti-cancer agent, its practical application is hindered by its limited water solubility and stability in vitro environments as well as its low bioavailability in vivo studies [270–272]. To face these challenges, the use of nanocarriers to deliver curcumin to the targeted tissue is suggested as a promising solution in vivo experiment [273–276]. In clinical practices, combining conventional anti-tumor medications with adjuvant treatments has shown complementary and supplementary role that enhanced the safety and effectiveness while minimizing adverse effects of the applied therapeutic strategies, as evidenced by numerous clinical trials [277–279], namely on breast cancer (NCT03980509), prostate cancer (NCT03769766), cervical cancer (NCT04294836) and advanced pancreatic cancer (NCT00094445). The following section provides a concise summary of the intricate mechanisms and specific targets through which curcumin acts against various types of cancer.

### Curcumin improves immunity

Curcumin exhibits promising immunomodulatory effects by interacting with a range of immune cell types (e.g., macrophages, DCs, MDSCs, NK cells, and T cells) in lung, breast, colon, liver, head and neck, ovarian, and tongue carcinoma [280, 281]. Curcumin enhances the activities of NK cells, DC cells, and B cells, and increases the quantity of cytotoxic T cells to eliminate cancer cells [282]. It also stimulates the proliferation of T cells, restores CD8^+^ T cells function, and revitalizes the defective T cells to kill cancer cells in colon carcinoma (HCT 116 and SW620) and head and neck cancer cells [283, 284]. Curcumin suppresses the development of liver cancer and lung cancer by decreasing MDSCs quantity in Hep G2 and Lewis lung cancer xenograft models, thereby reducing angiogenesis process [285, 286]. Furthermore, curcumin inhibits colon cancer (HCT 116 and SW620) and oral squamous cell carcinoma (CAL 27) cells by shifting TAMs from a M2 phenotype to M1 phenotype [287, 288]. In conclusion, curcumin improves immunological activity in cancer treatment or increases the number of immune cells, which strengthens innate immunity.

Cytokines play a complex role in tumor microenvironment, working as mediators of cancer progression and therapeutic agents [289, 290]. Curcumin inhibits tumor growth by reducing the levels of pro-inflammatory cytokines (e.g., TNF-*α*, IL-8, IL-6, and IL-1) [291–293], also down-regulating the protein expressions of these cytokines [294]. Additionally, curcumin suppresses the secretion of pro-cancer cytokines including stromal cell-derived factor 1 (SDF-1) and TGF-*β*1, which inhibits tongue fibroblasts from developing into squamous cell carcinoma [295].

Curcumin exerts immunomodulatory effects by modulating various immune checkpoints (PD-1, CTLA-4, and CD47, and PD-L2) facilitating the recognition and elimination of cancer cells by immune cells [283]. Curcumin enhances T lymphocyte activity to inhibit colon cancer (HCT 116 and SW620) and head and neck carcinomas (SNU1041 and SCC-15) cell growth by down-regulating PD-L1 protein expression [296, 297]. It also lowers CTLA-4 protein expression in CD4^+^CD25^+^ Tregs, thereby enhancing the effectiveness of cancer immunotherapy [298].

Curcumin's impact on immunological responses is commonly evaluated using in vitro and in vivo assays in the above cases. Nevertheless, enhancing the bioavailability and drug delivery of curcumin continues to be a crucial matter that needs to be resolved prior to its practical implementation.

### Curcumin reverses multidrug resistance

Curcumin could reverse multidrug resistance in cancer cells by inhibiting the function or expression of proteins such as P-gp, BCRP, multidrug resistance-related protein (MRP), and EGFR. This action helps counter resistance to first-line molecular drugs (Doxorubicin, Oxaliplatin, Paclitaxel, 5-Fluorouracil, and Cisplatin), or monoclonal antibodies (Cetuximab, Gefitinib, and Erlotinib), and immunotherapy drugs (Pembrolizumab and Atezolizumab) in various cancers including lung, colon, liver, breast, ovarian, myeloid leukemia, and osteosarcoma [299–302].

Specifically, curcumin sensitizes resistant cells of the lung (A549/Cisplatin), colon (SW620/DOX) and myeloid leukemia (K-562/DOX) to Doxorubicin and Cisplatin through inhibiting the transport function and protein expression of P-gp [303–305]. By preventing the accumulation of anti-cancer drugs within the cell, P-gp promotes resistance by preventing their cytotoxic or apoptotic effects. Its capacity to promote ATP-dependent drug translocation across the plasma membrane against significant concentration gradients allows it to accomplish this. Thus, P-gp inhibition would promote the influx of anti-cancer drugs inside the cell and result in cell cytotoxicity. Alternatively, the addition of a P-gp substrate in conjunction with the anti-cancer drug may have a comparable impact by competing for the transport pathway [306]. Furthermore, curcumin augments the effectiveness of Doxorubicin and Paclitaxel in overcoming MDR in lung cancer (A549), ovarian cancer (SK-OV-3), and colon cancer (HCT-8) cells by inhibiting both the protein function and expression of P-gp [307, 308]. Besides, curcumin conjugated with anti-P-gp antibody significantly improve the capability of targeting P-gp protein, thereby overcoming MDR in cervical cancer (KB-V1/DOX) cells [309].

In addition, curcumin also suppresses MRP in Oxaliplatin (L-OHP) resistance pancreatic cancer (PANC-1 and MIA PaCa-2) cells through its impacts on *miR-409-3p*, its curcumin-related mode of action increases L-OHP sensitivity and regulates the expression of ERCC1. This approach increases the drug's sensitivity by decreasing the colon cancer cells' release of L-OHP and colon cancer (HCT 116) cells and inhibits BCRP in mice model. BCRP is an ATP-binding cassette (ABC) transporter that actively extrudes medication into the intestinal lumen, hence decreasing the drug's oral bioavailability. Curcumin boosted Sulphasalazine's systemic exposure in a dose-dependent way [310–312].

Growth of human malignancies is commonly connected with abnormal cellular communication by inappropriate activation of receptor tyrosine kinases (RTK) following mutation, overexpression or ectopic ligand synthesis. RTK, including human epidermal growth factor receptor 2 (HER2), vascular endothelial growth factor receptor (VEGFR), and EGFR, can be overexpressed or upregulated in cancer cells. This can lead to various effects, including altered cell cycle regulation, increased cell proliferation, blocked apoptosis, increased motility and invasive capacity, metastasis-associated growth, and angiogenesis promotion [313].

Gefitinib and Erlotinib are oral tyrosine kinase inhibitors (TKIs) that are particularly effective in patients with specific mutations in the *EGFR* gene. Curcumin significantly augments the suppressive efficacy of gefitinib on H157, H1299, IEC-18, and NCI-H1975 cancer cells by facilitating the degradation of EGFR receptor [314, 315]. Curcumin can be used as an EGFR-TKI sensitizer to treat NSCLC with wild-type EGFR and a Kirsten rat sarcoma viral oncogene homolog (KRAS) mutation.

Furthermore, curcumin reduces EGFR protein expression in Erlotinib-resistant NSCLC (H1650) cells, hence enhancing the efficiency of Erlotinib, the recommended drug to combat lung cancer [299, 316]. The low response rate of lenvatinib, which is mostly caused by resistance development, limits its practical utility in treating patients with advanced liver cancer, even though it is the recommended initial treatment for this condition. Significant EGFR activation was seen in lenvatinib-resistant cell lines, and a genome-wide transcriptome profiling investigation linked lenvatinib resistance to the PI3K-AKT pathway. The EGFR-PI3K-AKT pathway's gene and protein expression were significantly inhibited by the combination therapy of curcumin and Lenvatinib. So, curcumin is shown to overcome Lenvatinib resistance and strengthens the anti-liver cancer efficacy in Huh-7 and PLC cells [317].

Besides, curcumin can enhance the therapeutic effect of pembrolizumab and atezolizumab by reversing the drug resistance [318]. Combining curcumin with an anti-PD-1 monoclonal antibody enhances the therapy efficacy in melanoma (B16-F10) and colon cancer (MC38) xenograft models [300, 319]. The findings indicate that curcumin has the capacity to regulate the function and expression of these transportation proteins, hence playing a key role in counteracting MDR of cancer cells.

### Curcumin suppresses cancer growth by regulating the processes of autophagy and ferroptosis

Autophagy and ferroptosis are two different forms of cell death that have attracted considerable attention in the realm of cancer treatment. Growing evidence shows that curcumin induces autophagy and ferroptosis in lung, liver, colon, breast, cervical, and renal cell carcinoma, which lead to cell death and tumor growth inhibition [320, 321].

Autophagy, a key form of cancer cell death, involves the breakdown and recycling of the cell's internal components. Multiple studies have shown that curcumin either induces or inhibits autophagy in a variety of in vitro or in vivo settings. Autophagy is frequently triggered by curcumin and its derivatives; However, this depends on the cellular environment and the stimulus. Furthermore, certain research has connected curcumin's potential to its iron-chelating properties. Curcumin has also been demonstrated to protect renal function by reducing ROS, reduce cancer by inhibiting rather than activating autophagy (an effect that may be mediated by the stimulation of *miR-143*), and lessen hypoxia/reoxygenation injury by controlling the activity of certain proteins [322].

Curcumin suppresses renal cell carcinoma (ACHN) and cervical cancer (SiHa) cells through the up-regulation of autophagy-related proteins, including p62, LC3B and Beclin-1 [323, 324]. Moreover, curcumin diminishes cancer stemness in triple negative breast cancer (MCF-7 and MDA-MB-231) cells by up-regulating gene and protein expression of ATG5 and ATG2B [325]. Additionally, curcumin causes cytotoxicity in thyroid and ovarian cancers [326, 327]. Furthermore, curcumin suppresses autophagy flux in breast cancer (MCF-7) and glioblastoma (A172 and U-87 MG) cells by decreasing the proportion of LC3-II/LC3-I and up-regulating p62 protein expression [328, 329].

Ferroptosis, a form of programmed cell death characterized by iron-dependent lipid peroxidation. Curcumin can regulate the ferroptosis-related gene (e.g., *MYC*, *IL-1β*, *EZH2*, *SLCA5*, and *CAV1*) expression to inhibit colon cancer (SW480) cell progression [330, 331]. Moreover, curcumin down-regulates the protein expression of GPX4 and Solute carrier family 7 member 11 (SLC7A11) to induce ferroptosis in colon cancer (HCT-8) and follicular thyroid cancer (FTC-133 and FTC-238) cells [332, 333]. Additionally, curcumin suppresses tumor growth in osteosarcoma (Saos-2 and U-2 OS) and breast cancer (MCF-7 and MDA-MB-453) cells by producing malondialdehyde (MDA), elevating ROS levels, and enhancing Fe^2+^ accumulation within cells [334]. Furthermore, curcumin decreases the expression of the *NCOA4, FTH1*, and *p53* genes, which causes ferroptosis in clear cell renal cell carcinoma (A-498-DR and 786-O-DR) cells [335]. Through the promotion of lipid peroxidation, disruption of iron homeostasis, and inhibition of antioxidant defenses, curcumin pushes cancer cells towards ferroptosis. The impact of curcumin on ferroptosis and autophagy offers substantial potential for improving cancer therapy approaches.

### Curcumin suppresses cancer progression via classical modalities

In the field of cancer research, the conventional exploration of anti-cancer strategies has primarily centered around studying cellular processes involved in cell cycle, apoptosis, and metastasis. In this regard, curcumin has been found to induce cell cycle arrest in G0/G1, S, and G2/M phases in various cancer types [336]. For example, curcumin treatment induces cell cycle arrest at the G0/G1 phase in liver cancer (SK-HEP-1 and Hep G2) and breast cancer (MCF-7) cells [337, 338]. In addition, curcumin treatment leads to cell cycle arrest at the S or G2/M phase in colon cancer (MC38 and HCT 116) cells, accompanied by a reduction in the protein expression of cell cycle-related markers (e.g., cyclin A2, cyclin E1, CDK2, and p21) [339, 340].

Curcumin reduces Cyclin D1 protein expression, inhibiting cell cycle, particularly at the G0/G1 phase, in colon, breast, and prostate cancers. Additionally, curcumin enhances p27 and p21 protein expression, thereby obstructing cell cycle advancement at the G0/G1 and S phases. Notably, curcumin elicits cell cycle arrest in several solid malignancies, including lung, colon, and neuroblastoma, predominantly affecting the G2/M phase [341–343]. Conversely, hematological cancers, including lymphoma and leukemia, often demonstrate cell cycle arrest at the G0/G1 phase [344–346]. Curcumin causes cell cycle arrest at the G2/M phase in both estrogen receptor (ER)-negative breast cancer (MDA-MB-231 and SKBR3) and (ER)-positive breast cancer (T-47D and MCF-7) cells [347–350]. Furthermore, HER-2 positive breast cancer (SKBR3 and 435eB) undergoes cell cycle arrest in the G0/G1 phase [351], while HER-2 negative breast cancer (MCF-7) cells mainly undergo cell cycle arrest at the S phase [352]. In addition, curcumin triggers cell cycle arrest at G0/G1 and S phases in triple negative breast cancer (BT-549 and MDA-MB-231) cells [353–355].

Curcumin triggers cell apoptosis in lung, colon, liver, cervical, and pancreatic cancers by up-regulating pro-apoptotic protein expression (Bax and Bad) or by inhibiting anti-apoptotic proteins (Bcl-xL and Bcl-2), as well as activating Caspase pathway [340, 356–359]. Specifically, it induces apoptosis in colon cancer (HCT 116) and lung cancer (A549) cells by activating apoptotic proteins, notably PARP, Caspase-3, and Caspase-8 [357, 360]. Furthermore, it reduces the protein expression of Bcl-2 and increases that of Bax, triggering apoptosis in cervical cancer (Ca Ski and Hela) and pancreatic cancer (BxPC-3) cells [356, 361].

The main Caspases implicated in cancer cell death through apoptosis include Caspase-8, Caspase-9, and the executioner Caspases, specifically Caspase-3 and Caspase-7. Curcumin activates Caspase-3 in several cancer types, resulting in the breakdown of cellular substrates and the successful initiation of programmed cell death (apoptosis). Caspase-8 is triggered by death receptors in the extrinsic pathway, while Caspase-9 is activated as a result of mitochondrial damage in the intrinsic pathway. Hence, curcumin suppresses cancer progression by inducing cell cycle arrest at various phases and promoting apoptosis through the modulation of key regulatory proteins, such as cyclins, CDKs, Bax, and Caspases. As for lung, liver, colon, breast, ovary, and nasopharyngeal tumors, curcumin inhibits migration and invasion [362–368]. It decreases the metastasis-related proteins (Vimentin, MMP-9, and E-cadherin) in breast cancer (MDA-MB-231) and lung cancer (A549) cells [362, 363]. These results suggest that curcumin demonstrates anti-tumor properties through the regulation of the cell cycle and the modulation of proteins associated with apoptosis and metastasis, hence effectively impeding cancer progression.

### Curcumin combined with other medications for cancer treatment

Curcumin has shown great potential for synergistic effects, reversing drug resistance, and enhancing sensitivity when combined with chemotherapeutic agents. In recent years, there has been an increasing interest in exploring this promising immunotherapy approach [369]. Studies have demonstrated that curcumin enhances the activity of PD-1/PD-L1 antibodies in murine colon cancer (MC38 and CT26) and liver cancer (Hep3B) cells by effectively preventing immune evasion [370, 371]. Growing evidence supports the potential effectiveness of curcumin combined with various chemotherapeutic agents, including Lenvatinib, Doxorubicin, Rapamycin, Paclitaxel, and Cisplatin, in treating diverse cancers such as breast, liver, colon, oral, and osteosarcoma [317, 372–377]. Besides, a synergistic approach utilizing curcumin and doxorubicin demonstrates enhanced anti-tumor efficacy against colon cancer (CT26), breast cancer (MCF-7), osteosarcoma (K-7), and head and neck cancer (HSC-3) cells [378–381]*.* A phase II clinical trial (NCT01490996) has shown that daily Folinic acid/5-fluorouracil/Oxaliplatin chemotherapy, when used alongside oral curcumin treatment, is safe and tolerable in patients with metastatic colon cancer [382]. Additionally, coadministration of supplemental curcumin with other first-line chemotherapy drugs (Avastin/FOLFIRI/Paclitaxel/Bioperine) demonstrates efficacy in treatment of breast cancer (NCT00852332), cervical cancer (NCT05947513), and myeloma (NCT00113841). The combination of Doxorubicin (0.4 mg/mL) and curcumin (5 µM) shows a higher additive impact by lowering the growth of Hodgkin lymphoma (L-540) cells by 79%. According to the pharmacokinetic analysis, curcumin (5 mg/kg) may also improve Doxorubicin (5 mg/kg) absorption and reduce drug efflux in vivo [383, 384]. For metastatic and advanced bladder cancer, Cisplatin-based combination therapy has become the accepted standard of care. In comparison to curcumin or Cisplatin alone, co-treatment with curcumin (10 µM) and cisplatin (10 µM) has demonstrated a strong synergistic effect by up-regulating ERK1/2 phosphorylation and activating Caspase-3 in bladder cancer cell lines [385]. It has been demonstrated that Celecoxib (selective COX-2 inhibitor) with curcumin together inhibits cancer cell progression in vitro more than Celecoxib solely. Curcumin (10–15 µmol/L) andCelecoxib (5 µmol/L) at physiological dosages showed a synergistic inhibitory effect against human colon cancer (HT-29) cells [386]. In both pre-clinical and clinical trials, these findings suggest that curcumin has substantial synergistic effects in inhibiting multiple types of cancer.

### Curcumin exerts its anti-cancer effects via regulating gut microbiota

The gut microbiota is crucial in the development and progression of various cancers including colon, liver, cervical cancers, and myeloid leukemia [387, 388]. Curcumin increases the abundance of beneficial bacteria like *Bifidobacteria* and *Lactobacillus*. Moreover, curcumin strengthens the gut barrier function, preventing the leakage of harmful substances that can induce systemic inflammation, thereafter, causing apoptosis in colon cancer (HT-29 and HCT 116) cells [389, 390]. In addition, curcumin sensitizes liver cancer (H22) cells to 5-fluorouracil cytotoxic effects by reducing the abundance of harmful bacteria (*Helicobacter* and *Campylobacteria)* [274]. Clinical research has been undertaken to investigate the physiological effects of curcumin on the microbiota of children with colon cancer and acute lymphoblastic leukemia (NCT05472753 and NCT05045443). Overall, the way curcumin interacts with the gut microbiota offers a potentially effective way to treat cancer. By modulating microbial composition, enhancing beneficial metabolites, and improving immune function, curcumin contributes to cancer prevention and treatment.

### Application of multi-omics techniques to investigate the anti-cancer properties of curcumin

Omics technologies encompass a broad range of fields including genomics, proteomics, metabolomics, and transcriptomics, each offering insights into the molecular and cellular processes of organisms. In cancer research, these technologies are invaluable because they provide comprehensive data about the genetic mutations, protein dysregulations, and metabolic changes associated with cancer [391–394]. Curcumin inhibits tumor growth in 4T1 xenograft model by regulating the breast cancer metabolism of amino sugars and nucleotide sugars [395]. Moreover, curcumin exerts its anti-lung cancer actions on A549 cells by regulating 25 differential metabolites that influence the fatty acid metabolism, sphingolipid metabolism, and glycerophospholipid catabolism in cancer cells [396]. Furthermore, curcumin selectively alters miRNA and mRNA expression, inducing apoptosis in ovarian cancer (A2780 and PA1) and breast cancer (MCF-7 and MDA-MB-231) cells, as demonstrated through mRNA and miRNA library sequencing [397, 398]. Furthermore, curcumin triggers cell death in pancreatic (BxPC-3) and breast cancer (MDA-MB-231) cells, disrupting 227 and 453 proteins, respectively, as identified through proteomics [356, 399]. Overall, omics technologies provide a holistic view of the cellular changes induced by curcumin, highlighting its potential as a versatile and effective anti-cancer agent.

### Curcumin derivatives inhibit cancer progression

Derivatives such as demethoxycurcumin (DMC), bisdemethoxycurcumin (BDMC), tetrahydrocurcumin (THC), and curcumin difluorinated (CDF) are fascinating advancements that expand curcumin's potential in fighting lung, colon, ovarian, cervical, and bladder cancers [400–402]. DMC induces apoptosis in lung cancer (A549) and bladder cancer (RT4 and TCCSUP) cells by activating Caspase-3 and Caspase-9 activity, and inhibiting the expression of anti-apoptotic proteins (Bcl-2 and Bcl-xL) [402, 403]. In addition, BDMC suppresses breast cancer (BT-549, MDA-MB-231, and MCF-7) progression through down-regulating cancer metastasis-related proteins (MMP-9 and TWIST1) and inducing pro-apoptotic proteins (p53) expression [404, 405]. Besides, a clinical trial (NCT06063486) has been performed to assess the efficacy of DMC and BDMC in reducing tumor markers and improving patient survival rates following treatment therapy. In summary, curcumin derivatives have the potential to both improve curcumin's overall efficacy and rectify its disadvantages.

### Investigating curcumin anti-cancer effects through drug delivery system

The therapeutic efficacy of curcumin, however, is restricted by its poor solubility, low absorption from the gut, rapid metabolism, and systemic elimination. To overcome these challenges, researchers have been exploring various drug delivery systems such as nanoparticle encapsulation, liposomes, polymeric micelles, dendrimers, hydrogels that can enhance curcumin's bioavailability and therapeutic efficacy [406–413]. Nanoparticle-based curcumin drug delivery systems (DDS) enhance apoptosis and inhibit cell migration in breast cancer (MDA-MB-231) and lung cancer (A549) cells [413, 414]. Moreover, curcumin-encapsulated micelles induce cancer cell apoptosis, initiate a halt in the cell cycle, and enhance cellular uptake of curcumin in gastric cancer (BGC-823 and SGC-7901), colon cancer (HT-29 and HCT 116), breast cancer (MCF-7), and glioblastoma multiforme (GL261) cells [407, 412, 415, 416]. In addition, nanogel loaded with curcumin such as AuNP@Ng/Cur, hyaluronic acid-grafted poly(N-isopropylacrylamide), and PVAS@PEG nanogels shows better targeting and low toxicity in breast cancer (MDA-MB-231) and melanoma (B16-F10) cells [417–419]. Besides, when compared to free curcumin, curcumin-loaded liposomes exhibit enhanced cytotoxicity to cancer cells in liver cancer (H22 and Hep G2) and breast cancer (SKBR3 and MCF-7) cells [420, 421]. Taking together, these innovative carrier materials not only address the limitations associated with curcumin's natural properties but also enhance its targeting and intake into cancer cells.

Exosomes released by numerous types of cells and contain a vast spectrum of bioactive components including proteins, lipids, and nucleic acids. Exosomes can deliver their payload through direct cell-to-cell contact or without it. Exosomes have garnered significant interest for medication delivery in clinical settings due to their ability to operate as intracellular communication vehicles, transferring content to targeted destination [422]. Since most exosomes are hydrophobic and lipid-enriched, they can be used to deliver therapeutic substances like curcumin. Curcumin's plasma concentration and bioavailability were both enhanced by the in vivo injection of curcumin via exosomes. When 100 mg/kg body weight curcumin is administered orally or intraperitoneally, a plasma concentration of 1,250 ng/ml is reached after 30 min (5–10 times greater than when curcumin is administered alone), and this concentration lasts for 12 h [423]. The solubility, stability, and bioavailability of curcumin are all increased when it is incorporated into exosomes [424]. Curcumin loaded exosomes (Exo-CUR) was administered orally to nude mice containing the cervical Ca Ski-tumor xenograft to assess its in vivo anti-cancer efficacy. Curcumin diet, exosomes by themselves, and PBS were used as controls. Exosomes demonstrated a slight (25–30%) reduction of tumor growth, whereas dietary curcumin had no impact. On the other hand, the cervical tumor xenograft growth was significantly inhibited by Exo-CUR (61%; *p* < 0.01). Rats given Exosomes or Exo-CUR did not exhibit any signs of gross or systemic toxicity [424]. Exo-CUR extracted from lung cancer (H1299) cells treated with curcumin (10 μM) produced a slowdown in proliferation, colony formation, migration, and invasion. By down-regulating DNMT1, curcumin via exosomes is able to upregulate TCF21 expression and function as a tumor suppressant [425]. Curcumin treatment of chronic myelogenous leukaemia (CML) cells resulted in a dose-dependent rise in phosphatase and tensin homolog (PTEN), the *miR-2*1 target. Moreover, curcumin therapy reduces VEGF expression and releases AKT phosphorylation. Assays for colony formation show that curcumin had an impact on CML cells' ability to survive. Certain observations imply that exosomes may be involved in the cellular disposal of miRNAs. To determine whether curcumin led to a reduction in *miR-21* in CML cells and its exosome packaging, authors examined the content of *miR-21* in K-562 and LAMA-84 cells as well as exosomes following curcumin treatment. Additionally, authors demonstrated that adding curcumin to CML cells resulted in an increase in *miR-196b* cellular expression and a downregulation of Bcr-Abl expression [426]. Moreover, curcumin-encapsulated grapefruit exosomes can alleviate colitis and boost mouse survival. When curcumin-encapsulated grapefruit exosomes are administered to DSS-induced colitis-affected mice, colon tissue extracts showed lower levels of TNF-*α*, IL6, and IL-1*β* in comparison to other groups, according to ELISA analysis [427].

In sum, curcumin can engage multiple anti-tumor pathways, spanning immunity, autophagy, ferroptosis, and classical cell cycle arrest. It underscores the broad therapeutic scope, yet its real-world impact depends on overcoming poor pharmacokinetics through innovative delivery strategies (nanoparticles and exosomes) and synergistic combinations. Emerging multi-omics insights reveal how curcumin fine-tunes cancer cell metabolism, immune checkpoints, and even microbial ecosystems, positioning it as a uniquely adaptable agent in the quest for personalized oncology solutions.

### Emodin

Emodin is a naturally occurring anthraquinone found in the roots and barks of various plants, which is an active constituent in medicinal herbs, such as Rhei radix et rhizoma (Dahuang in Chinese) and Polygoni cuspidati rhizoma et radix (Huzhang in Chinese) [428–430]. Additionally, emodin is synthesized as a secondary metabolite by molds and lichens [431, 432]. Emodin possesses a myriad of pharmacological effects including anti-oxidant, anti-inflammatory, anti-tumor, anti-ulcer, hepatoprotective, renal-protective, neuroprotective, antidiabetic, antibacterial, antiviral, muscle relaxant, immunosuppressive and antifibrotic activities, and therapeutic effects on hypertrophic scar [433–435]. Emodin also demonstrates a broad spectrum of anti-cancer characteristics, showing promise against a number of cancers, including melanoma, pancreatic, thyroid, nasopharyngeal, cervical, endometrial, gastric, prostate, lung, breast, colon, kidney, liver, ovarian, and leukemia [436]. The related molecular mechanisms corresponding to the anti-cancer activities of emodin are involved in the modulation of tumor immunity, induction of apoptosis, inhibition of cell proliferation, and induction of autophagy, necroptosis, ferroptosis and uncoupling, mitigation of drug resistance [437]. In addition, in silico methods are employed to predict the pharmacological properties of emodin based on its interaction with carbonic anhydrase IX, and these predictions are subsequently validated through in vitro experiments on the HCT 116 and HeLa cell lines [438, 439]. In addition, zebrafish are utilized to assess the antiangiogenic effects of emodin, which effectively inhibited vascular development and tumor angiogenesis in vivo, highlighting its potential for cancer treatment [440]. To address the challenges of poor intestinal absorption, rapid elimination, and low bioavailability of emodin, and to enhance its cellular uptake and anti-cancer efficacy, various drug delivery systems have been employed to encapsulate emodin, resulting in improved antitumor efficacy against multiple cancer types [441–444]. The following section provides a concise summary of the intricate mechanisms and specific targets through which emodin acts against various types of cancer.

### Emodin regulates tumor immunity

Emodin emerges as a promising anti-cancer agent through its multifaceted mechanisms that target neutrophils, macrophages [445]. Neutrophil extracellular traps (NETs) have emerged as key effectors in neutrophil responses to diverse stimuli, which play a pivotal role in cancer progression and associated thrombosis. This underscores the intricate interplay between neutrophils, hypercoagulation, and carcinogenesis [446]. Emodin exhibits a novel anti-cancer mechanism by suppressing N2 neutrophils, thereby mitigating hypercoagulation and preventing lung carcinogenesis. In the urethane-induced lung carcinogenic model, emodin reduces hypercoagulation and tumor lesions, accompanied by a decrease in N2 neutrophils and modulated cytokine profiles (IFN-*γ*, IL-12 up; IL-6, TNF-*α*, TGF-*β*1 down). Immunohistochemical and network pharmacology data further supported multi-target effects of emodin on N2 neutrophils, inhibiting tumor growth and promoting lung health [445]. TAMs, an essential subset of immune cells infiltrated in the tumor microenvironment, is found affected EMT and cancer stem cell formation [447]. Furthermore, it inhibits EMT and cancer stem cell formation in breast cancer cells to halt metastatic recurrence by disrupting TGF-*β*1-mediated interactions between TAMs and breast cancer cells [448]. Emodin exerts anti-tumor effects against liver cancer by modulating M2 macrophage polarization towards M1 phenotype via the *miR-26a*/TGF-*β*1/AKT axis, thereby inhibiting cancer cell proliferation and invasion [449]. Furthermore, emodin reduces the population of M2-like protumorigenic macrophages within the tumor microenvironment. Moreover, emodin may act by antagonizing the P2X7 receptor, thereby decreasing the activation of M1 macrophages and potentially inhibiting their recruitment to the tumor microenvironment [450]. Emodin, targeting neutrophils and macrophages, exhibits anti-cancer potential by mitigating hypercoagulation and modulating cytokine profiles in lung cancer. It also disrupts the tumor-promoting loop between tumor cells and TAMs, inhibiting tumor growth, EMT, cancer stem cell formation, and metastatic recurrence in breast cancer. Emodin also has anticancer effects by lowering M2-like macrophage numbers and altering macrophage polarization.

### Emodin suppresses cancer growth by regulating the processes of necroptosis, autophagy, ferroptosis and uncoupling

Emodin demonstrates anti-cancer activity against prostate, renal, glioma, liver, colon cancers, and melanoma by inducing necroptosis, autophagy, and ferroptosis through various signaling pathways [451, 452]. Emodin significantly reduces viability in prostate cancer (PC-3 and DU 145) cells. Emodin activates the necroptosis pathway, enhancing MLKL and HSP90AA1 expression and activating the PGAM pathway, leading to mitochondrial fission and ROS generation [453]. Emodin can effectively kill renal cancer cells. It induces necroptosis via a ROS-mediated activation of the JNK signaling pathway and also inhibits glycolysis by down-regulating glucose transporter 1 (GLUT1) through ROS-mediated inactivation of the PI3K/AKT signaling pathway [451]. Emodin effectively inhibits glioma (U251) cell proliferation, inducing apoptosis and necroptosis via the TNF-*α*/RIP1/RIP3 signaling axis. Its ability to upregulate TNF-*α*, RIP1, RIP3, and MLKL both in vitro and in vivo suggests emodin as a potential anti-cancer agent targeting glioma through necroptosis [454]. Emodin inhibits liver cancer progression by modulating autophagy through the *miR-371a-5p*/PTEN axis, down-regulating *miR-371a-5p* and up-regulating PTEN, thereby enhancing autophagy markers LC3-II and reducing p62 [455]. Emodin-induced autophagy promoted Snail and *β*-catenin protein degradation, inhibiting EMT partly through the PI3K/AKT/mTOR and Wnt/*β*-catenin pathways. Therefore, emodin plays a significant role in inhibiting liver cancer (HepG2) cell metastasis via the interplay between autophagy and EMT [456]. Emodin inhibits proliferation and induces ferroptosis in colon cancer (HT-29, RKO, HCT-15 and SW620) cells by inactivating the NF-*κ*B pathway and nuclear receptor coactivator 4 (NCOA4)-mediated ferritinophagy. Emodin decreases GSH content, xCT and GPX4 expression, increases ROS generation, MDA, and lipid peroxidation, effects reversed by ferroptosis inhibitors and autophagy inhibitors, iron chelator DFO, ferostatin-1, and NCOA4 silencing. Emodin is expected to be a promising candidate for anti-cancer therapy [452]. Emodin exerts antiproliferative effects on melanoma (B16-F10) cells by reducing ATP levels and functioning as a mitochondrial uncoupler [457]. Emodin stands out as a versatile and promising candidate for anticancer therapy due to its capacity to engage multiple cell death pathways and interfere with cancer cell metabolism and survival. The depth of its impact on cellular mechanisms such as necroptosis, autophagy, ferroptosis, and mitochondrial function underscores its potential in the clinical setting.

### Emodin inhibits tumor recurrence, and cancer-related cachexia

Emodin halts nasopharyngeal carcinoma recurrence, and alleviates lung cancer-related cachexia symptoms, demonstrating potential as a chemopreventive and therapeutic agent in nasopharyngeal carcinoma and lung cancer [458, 459]. Emodin inhibits SP1 protein expression, suggesting its role in suppressing Epstein-Barr virus (EBV) reactivation. Furthermore, emodin inhibits the tumorigenic properties induced by repeated EBV reactivation. Emodin administration also restrains tumor growth in mice induced by EBV activation, collectively supporting its potential as a chemopreventive agent against EBV reactivation and nasopharyngeal carcinoma recurrence [459]. Emodin inhibits transcription Factor 4 (TCF4)- Twist family bHLH transcription factor 1 (TWIST1) complex formation, thereby suppressing parathyroid hormone-like hormone (PTHLH) protein expression, which are implicated in lung cancer cachexia. In lung cancer (A549) tumor-bearing mice, emodin-containing extract alleviates cachexia symptoms by down-regulating fat browning-related genes and skeletal muscle atrophy, highlighting its potential as a therapeutic agent for lung cancer-related cachexia [458]. Emodin shows promise as a chemopreventive and therapeutic agent against cancer-related complications such as tumor recurrence and cachexia. Its ability to inhibit EBV reactivation and tumor growth in nasopharyngeal carcinoma, as well as alleviating cachexia symptoms in lung cancer, underscores its multifaceted therapeutic potential.

### Emodin suppresses cancer progression via classical modalities

Emodin, a versatile natural compound, exerts its antitumor effects through multiple mechanisms, including cell cycle arrest, apoptosis induction, angiogenesis inhibition, migration, and invasion suppression and tumor metastasis inhibition in NSCLC, breast, ovarian, endometrial, liver, cervical, colon, endometrial cancers, as well as melanoma, and papillary thyroid carcinoma [448, 460]. Emodin can suppress cell growth through inducing cell cycle arrest at G0/G1, S and G2/M stages, such as G0/G1 arrest in NSCLC cells via hyaluronan synthase 2 (HAS2)-mediated signaling [461], and S and G2/M phases arrest in liver cancer (Hep G2) cells [456]. This cell cycle inhibition is further supported by its ability to induce mitotic catastrophe and G2/M phase arrest in cervical cancer (HeLa) cells. It highlights its efficacy in disrupting cell division [462]. Furthermore, emodin promotes apoptosis through multiple pathways. It increases ROS levels, leading to DNA damage and apoptosis in NSCLC (NCI-H520, NCI-H460, and A549) cells [463]. In endometrial cancer (KLE) cells, emodin regulates MAPK and PI3K/AKT signaling to enhance ROS production and apoptosis [464]. Similarly, in liver cancer, emodin upregulates oxidative stress defense and tissue homeostasis [465]. Emodin exhibits pro-apoptotic effects in melanoma (B16-F10 and A375) cells by increasing the Bax/Bcl-2 ratio, and suppressing the Wnt/*β*-catenin pathway [466]. Emodin effectively suppresses the viability and migration of B16-F10 cells and may induce apoptosis via the mitochondrial pathway or the death receptor-mediated pathway [467]. Moreover, emodin exhibits anti-cancer activity in breast, colon, and liver cancers by targeting various pathways and suppressing VEGF-related angiogenesis and tumorigenesis. In triple-negative breast cancer (MDA-MB-231 and 4T1) cells, emodin targets nuclear receptor corepressor 2 (NCOR2) to upregulate seryl-tRNA synthetase (SerRS), suppressing VEGFA transcription and angiogenesis [440]. In colon cancer (HCT 116) cells, emodin reduces VEGF secretion and VEGFR1/2 protein expression by down-regulating Acyl-CoA synthetase long chain family member 4 (ACSL4) [468], while in liver cancer, emodin inhibits tumorigenesis through concurrently suppressing the VEGFR2-AKT-ERK1/2 signaling pathway, and inhibiting the protein expression of SMAD2/4 by enhancing *miR-34a* level in Hep G2 cells [469].

In addition, emodin demonstrates anti-migratory, anti-invasive and anti-metastasis properties. In ovarian cancer (SK-OV-3, A2780 and PA1) cells, it hinders migration and invasion by inhibiting EMT and altering cadherin protein expression [470]. In melanoma (B16-F10 and A375) cells, emodin decreases MMP-2 and MMP-9 protein expression, suppressing migration and invasion [466]. Emodin downregulates proliferation, invasion markers in liver cancer in vivo [465]. Emodin inhibits tumor metastasis in breast cancer by disrupting TGF-*β*1-mediated TAM-cancer cell interactions to halt EMT and stem cell formation [448], and modulating AKT/ERK signaling to attenuate metastatic potential in obesity/hyperlipidemia [471]. Furthermore, emodin modulates signaling pathways critical for tumor growth and progression. Emodin inhibits breast cancer (MCF-7) cell proliferation in a time- and dose-dependent manner. Elevated AhR and CYP1A1 protein expression post emodin treatment suggests the antitumor activity of emodin via AhR-CYP1A1 pathway activation [472]. In NSCLC (A549, H1650, H460, H1975, PC-9, H1299, and CCD-19Lu) cells, it inhibits KRAS mutant cell lines by reducing secretory phospholipase A2-IIa (sPLA2-IIa) and NF-*κ*B pathway activity, while inhibiting mTOR and AKT while activating AMPK [473]. Emodin inhibits endometrial cancer (KLE) cell proliferation by regulating MAPK and PI3K/AKT signaling pathways [464]. Emodin demonstrates efficacy in suppressing liver cancer (Hep G2 and Huh7) cell growth, metastasis, and CD44^+^ hepatoma cell proliferation [474]. In papillary thyroid carcinoma (TPC-1 and IHH4) cells, emodin downregulates NF-*κ*B and TLR4 signaling, inhibiting cell proliferation, invasion, and migration [475].

Emodin has the potential to be used as a photosensitizer in photodynamic therapy of skin cancer. Emodin mediates photodynamic therapy effectively and distinctly on skin cancer cells (SCC-25, MUG-Mel2) and normal keratinocyte (HaCaT) cells [476]. In melanoma (B16-F10 and A375) cells, its ability to decrease MMP-2 and MMP-9 protein expression and suppress the Wnt/*β*-catenin pathway suggesting a therapeutic strategy for highly metastatic tumors [466].

Emodin inhibits breast cancer metastasis by reducing lipid synthesis through the downregulation of triglyceride synthesis-related genes, including *Fas*, glycerol-3-phosphate acyltransferase 1 (*Gpat1*), and stearoyl-CoA desaturase 1 (*Scd1*). Additionally, emodin attenuates the high metastatic potential to the liver in the context of obesity and hyperlipidemia through modulation of the AKT and ERK signaling pathways, involving interaction with CSNK2A1 in breast cancer [471].

Therefore, emodin demonstrates antitumor effects through mechanisms such as cell cycle arrest, apoptosis induction, and angiogenesis and metastasis inhibition. In a variety of cancer types, it alters the expression of biomarkers and targets different signaling pathways.

### Emodin combined with other medications for cancer treatment

Emodin, when combined with various medications, demonstrates synergistic anti-cancer effects by enhancing apoptosis, inhibiting proliferation, migration, and invasion, and reversing drug resistance, and mitigating chemotherapy-induced testicular toxicity in breast, lung, colon, cervical, liver, pancreatic cancers, as well as chronic myeloid leukemia and multiple myeloma [477, 478]. Emodin in combination with berberine significantly inhibits salt-inducible kinase 3 (SIK3) activity, reducing cell growth and inducing cell cycle arrest and apoptosis in breast cancer cells. Mechanistically, the combination treatment attenuates SIK3-potentiated mTOR-mediated aerobic glycolysis and suppresses AKT signaling, leading to G0/G1 phase cell cycle arrest and apoptosis in a SIK3-dependent manner. These findings suggest that the emodin-berberine combination may serve as a novel SIK3 inhibitor for the prevention and treatment of breast cancer (MDA-MB-231, BT-20, and AU565) cells [479]. Thymoquinone and emodin synergistically inhibit breast cancer (MCF-7) cell proliferation, enhancing cytotoxicity, and inducing apoptosis while curtailing migration and stemness [480]. Emodin synergistically enhanced the antiproliferative effects of paclitaxel in NSCLC (A549) cells, augmenting apoptosis through up-regulating the protein expression of Bax, Bcl-2, as well as activating the phosphorylation of AKT, and ERK, as well as activating Caspase-3. In vivo, emodin potentiated the antitumor effects of paclitaxel in A549 xenografts without significant side effects, suggesting a promising combinational strategy for NSCLC treatment [481]. Emodin inhibits proliferation and drug efflux, enhances Cisplatin (DDP)-induced apoptosis and DNA damage, and decreases P-gp protein expression in a dose-dependent manner in NSCLC (A549 and H460) cells. These findings suggest emodin as a potential adjuvant to enhance DDP sensitivity in NSCLC [482]. Meanwhile, emodin synergizes with DDP to enhance lung cancer A549/DDP cell apoptosis and impede migration/invasion, likely via inhibiting NF-*κ*B signaling and down-regulating drug resistance proteins (P-gp, MRP, and GST), thereby reversing DDP resistance and augmenting intracellular drug accumulation [483]. Emodin enhances anti-proliferation of 5-fluorouracil and inhibits invasion and migration of 5-fluorouracil-resistant colon cancer (SW480) cells. The combination induces apoptosis by regulating the protein expression of Bcl-2 and Bax, activating Caspase-3, and reversing 5-fluorouracil resistance through downregulation of the PI3K/AKT pathway. In vivo, emodin reverses 5-fluorouracil resistance in colon cancer xenografts, indicating its potential as a molecular therapy for 5-fluorouracil-resistant colon cancer (SW480/5-FU) cells [484]. In vivo, emodin and tetrahydropalmatine synergistically reduces colon cancer (HT-29) tumor size, increasing F4/80 expression, decreasing IL-6 and TGF-*β* levels, promoting tumor oxidation, and down-regulating p53 and STAT3 protein expression. These findings suggest a synergistic mechanism involving cytokine regulation and p53 pathway inhibition for the treatment of colon cancer [485]. The combination of emodin and vinblastine has been demonstrated to enhance the sensitivity of cervical cancer (HeLa) cells to the latter. This combination has been shown to potentiate the cytotoxic effect of vinblastine, disrupt mitosis and block cells in the G2/M phase. Furthermore, it has been shown to raise ROS production and oxidative stress, which causes edema and mitochondrial damage. Additionally, the combination of emodin and vincristine enhances cell apoptosis by activating Caspase-3 and Caspase-7, and decreasing Bcl-2 protein expression [486]. Meanwhile, emodin enhances the sensitivity of liver cancer (Hep G2) cells to DDP by inhibiting EMT [487]. Emodin inhibits cell activity across cell lines, enhancing apoptosis synergistically with gemcitabine in pancreatic cancer. Emodin potentiates the anti-cancer effects of EGFR inhibitor on pancreatic cancer (PANC-1 and BxPC-3) cells by inhibiting STAT3 signaling, promoting apoptosis. The combination of emodin and EGFR inhibitor synergistically inhibits cancer cell proliferation in vitro and in vivo, modulating STAT3 protein expression [488]. Emodin and berberine combination exhibits synergistic anti-tumor effects, inhibiting cell proliferation, clonogenicity, migration, and invasion in pancreatic ductal adenocarcinoma (BxPC-3 and MIA PaCa-2) cells. This synergism has significant anti-cancer effects on pancreatic ductal adenocarcinoma via targeting EGFR-signaling through Laminin subunit beta 3 (LAMB3) regulation [489].

Emodin and 3'‑azido‑3'‑deoxythymidine (AZT) exerts the synergistic antitumor effects on human chronic myeloid leukemia (K-562) cells. Emodin-AZT combination reduces toxicity, improving efficacy, and modulating growth, apoptosis, and Wnt/*β*-catenin signaling via EGR1 [490]. Emodin suppresses imatinib resistance in chronic myeloid leukemia (K-562/ADM) cells via Bcr-Abl and STAT5 downregulation, alongside allosteric inhibition mechanisms [491]. Emodin significantly mitigates cyclophosphamide-induced testicular toxicity in rats by improving sperm function and reproductive hormones levels, reversing oxidative stress and inflammation, and increasing testicular antioxidant enzymes, demonstrating its potential to counteract chemotherapy-related testicular damage [492]. Emodin synergized with carfilzomib to reduce proliferation and viability in multiple myeloma cells, increasing ROS production and inducing apoptosis and autophagy pathways via activating Caspase-3 and PARP [493]. Emodin combined with telomerase inhibitor BIBR1532 synergistically enhances telomere dysfunction and inhibits 4T1 xenograft tumor growth, highlighting a potential strategy for cancer treatment [494]. The combination of emodin with various pharmacological agents holds significant promise in cancer therapy, offering synergistic effects that enhance apoptosis, overcome drug resistance, and improve therapeutic outcomes. By targeting multiple pathways involved in cancer cell proliferation, migration, and resistance mechanisms, emodin enhances the efficacy of conventional and targeted therapies.

### Emodin derivatives inhibit cancer progression

Emodin derivatives, particularly aloe-emodin, demonstrate broad-spectrum anti-cancer activity against multiple tumor types. Emodin derivatives inhibit cancer cell proliferation, promotes apoptosis, and disrupts oncogenic signaling pathways in prostate, breast, lung, colon, melanoma, nasopharyngeal, pancreatic, and glioma cancers [495, 496]. It achieves this through mechanisms such as oxidative stress induction, modulation of apoptotic proteins, inhibition of telomerase activity, and disruption of cell cycle progression. Moreover, *β*-Dihydroartemisinin-emodin and emodin-8-O-glucoside can inhibit anti-cancer potential in liver cancer, neuroblastoma, and glioblastoma [497, 498], underscoring the therapeutic promise of emodin derivatives in cancer treatment.

Emodin and aloe-emodin share an isomeric relationship, with their sole distinction lying in the placement of a single hydroxyl group. Aloe-emodin demonstrates broad-spectrum anti-cancer activity against various tumor types, including prostate, breast, lung, colon, cervical cancer, melanoma, nasopharyngeal, liver, pancreatic cancers and glioblastoma [499–510]. In prostate cancer, aloe-emodin exhibits anti-cancer effects against DU 145 cells by inducing ROS-mediated oxidative stress, apoptosis, mitochondrial dysfunction, and inhibiting Wnt/*β*-catenin signaling [500]. Similarly, in breast cancer (MCF-7 and MDA-MB-231) cells, aloe-emodin selectively inhibits proliferation and promotes apoptosis by modulating Bcl-2 protein levels through *miR-15a*/*miR-16–1* upregulation [501], competitively inhibiting telomerase and stabilizing G-quadruplex structure, suppressing hTERT transcription in breast cancer (MDA-MB-453, MDA-MB-231, and MCF-7) cells through E2F1 upregulation and c-Myc downregulation, as well as promoter demethylation [502]. At the same time, aloe emodin can also inhibit the metastasis of breast cancer (MCF-7) cell [503]. Aloe-emodin reverses adriamycin (ADR) resistance in MCF-7/ADR cells by inhibiting P-gp efflux, altering energy metabolism, and inducing autophagy while enhancing apoptosis via DNA damage, ROS generation, and Caspase-3 activation [504]. Furthermore, aloe-emodin exhibits anti-cancer activity against NSCLC (A549 and NCI-H1299) through Caspase-dependent apoptosis, autophagy induction via MAPK activation and AKT/mTOR inhibition, and ROS-mediated autophagy-triggered apoptosis, demonstrating its potential as a therapeutic candidate and synergize with gemcitabine [505]. In colon cancer (HCT 116) cells, aloe-emodin inhibits cancer cell proliferation and induces apoptosis via the mitochondria-mediated pathway, involving Bax/Bcl-2 modulation, Caspase activation, and cytochrome C release [506]. Additionally, aloe-emodin potently inhibits melanoma (A375 and SK-MEL-28) growth and metastasis by enhancing apoptosis, inducing G2 arrest, and modulating Wnt/beta-catenin signaling [507]. Aloe-emodin inhibits nasopharyngeal carcinoma cell (5-8F and CNE1) proliferation, migration, and promotes apoptosis by regulating Dual specificity phosphatase 1 (DUSP1), blocking ERK-1/2, AKT, and p38-MAPK pathways, and stabilizing DUSP1 via ubiquitin–proteasome inhibition [508]. Aloe emodin exerts dose-dependent cytotoxicity in pancreatic carcinoma (MIA PaCa-2 and PANC-1) cells, inducing apoptosis and autophagy, arresting cell cycle, and disrupting mitochondrial membrane potential, thereby inhibiting cancer cell growth [509]. Lastly, in drug-resistant glioma, aloe-emodin enhances the anti-cancer effects of temozolomide (TMZ) in drug-resistant glioma (NULU and ZAR) cells by restoring drug sensitivity, inhibiting colony formation, and slowing migration, indicating the potential of aloe-emodin as a natural adjuvant in glioblastoma treatment [510].

*β*-Dihydroartemisinin-Emodin exhibits potent anti-cancer activity in liver cancer (Hep G2) cells by inhibiting proliferation, promoting apoptosis via cell cycle arrest and Caspase-mediated pathways, and inhibiting migration [498]. Emodin-8-O-glucoside inhibits viability and proliferation of neuroblastoma and glioblastoma cells (SK-N-AS, T98G, and C6) in a dose-dependent manner [497]. The therapeutic potential of these derivatives in the treatment of cancer is shown by the strong action of *β*-Dihydroartemisinin-Emodin in liver cancer and the inhibition of neuroblastoma and glioblastoma cell growth by Emodin-8-O-glucoside. Therefore, aloe-emodin’s ability to reverse drug resistance, inhibit metastasis, and enhance the effectiveness of other chemotherapeutic agents, such as gemcitabine and temozolomide, underscores its potential as a valuable adjuvant in cancer therapy. Their mechanisms of action, which include the induction of oxidative stress, modulation of apoptosis, inhibition of telomerase, disruption of cell cycle progression, and interference with oncogenic signaling pathways, make them attractive candidates for cancer treatment.

### Application of multi-omics techniques to investigate the anti-cancer properties of emodin

Emodin significantly reduces the viability of lung cancer cells and triggers apoptosis. After treatment with emodin, the analysis of the transcriptome using RNA-seq reveals that 65 genes showed a significant change in expression in A549 and H1650 cells. In vitro qRT-PCR results confirm the up-regulation of 4 genes and the downregulation of 9 genes in A549 and H1650 cells. In vivo qRT-PCR results shows similar gene expression changes in tumor tissues compared to the control group: up-regulation of 3 genes; down-regulation of 6 genes [511].

Aloe emodin and emodin inhibit breast cancer metastasis by suppressing adhesion, invasion, and angiogenesis, and inducing anoikis in a co-culture model of MCF-7 and HUVEC cells. Metabolomics analysis identifies 27 and 13 biomarkers for aloe emodin and emodin, respectively, implicating pathways such as polyamine metabolism, the methionine cycle, the TCA cycle, purine metabolism, GSH metabolism, and aspartate synthesis. Quantitative analysis confirmed that the inhibitory effect of aloe emodin is better than that of emodin, suggesting distinct mechanisms for each compound against breast cancer metastasis [503]. Cell metabolomics is used to investigate emodin's toxicity and mechanism, revealing differences in 33 and 23 metabolites in Hep G2 cells extracts and media, respectively, affecting 8 pathways. The results demonstrate the potential of cell metabolomics in clarifying emodin's toxic effects, mechanisms, and biomarkers, aiding in the discovery of drug toxicity targets and signal network changes [512]. Therefore, omics approaches enhance understanding of emodin's antitumor effects. RNA-seq in lung cancer cells shows altered gene expression post-emodin treatment. The qRT-PCR confirms differential gene regulation both in vitro and in vivo.

### Emodin exerts its anti-cancer effects through the drug delivery system

Emodin, when encapsulated in various drug delivery systems, demonstrating enhanced cancer cell killing efficiency, suppressed tumor cell metastasis, and improved antitumor efficacy. effectively improve bioavailability, cellular uptake, and anti-cancer mechanisms of emodin. These drug delivery systems include magnetic liposomal nanocomposites, targeted liposomes, polymer lipid hybrid nanoparticles, pH/ion-responsive hydrogels, EGF-modified micelles, hybrid membrane-coated nanoparticles, and Fe_3_O_4_-PEG-Cy7-coated formulations. These systems demonstrate enhanced antitumor efficacy against diverse types of cancer, such as breast cancer, lung cancer, ovarian cancer, melanoma, pancreatic cancer, and gastric cancer [441–444, 513–517].

At low concentrations, the magnetic liposomal emodin nanocomposite containing ferromagnetic iron oxide nanocubes dramatically increases the effectiveness of killing MCF-7 cancer cells [441]. A targeted liposome is developed through the modification of arginine-8-glycine-aspartic acid (R_8_GD) to encapsulate emodin and daunorubicin separately. The combination of these R_8_GD modified liposomes demonstrated potent toxicity against highly invasive breast cancer (MDA-MB-435S) cells, effectively suppressing the formation of vasculogenic mimicry (VM) channels, and inhibiting tumor cell metastasis by down-regulating HIF-1*α*, MMP-2, TGF-*β*1 and VE-cad [442]. Emodin-loaded polymer lipid hybrid nanoparticles (E-PLNs) exhibited superior anti-cancer efficacy against breast cancer (MCF-7) cells by enhancing uptake, promoting early apoptosis via Bax/Bcl-2 pathway, and inhibiting tumor growth by over 60% in mice. The therapeutic effect of E-PLNs surpasses that of free emodin, highlighting their potential as an effective anti-breast cancer agent [514]. The *γ*-irradiated bovine albumin nanoparticle, functionalized with folic acid and carrying emodin, demonstrates enhanced activity against MCF-7 cancer cells and induced an immune response in macrophages [443].

Emodin encapsulated in micelles, surface-modified with EGF as a targeting ligand, and formulated in a compound preparation. EGF-modified emodin micelles are characterized for their physical and chemical properties, showing enhanced uptake and antiproliferative effects in ovarian cancer (SK-OV-3) cells. The compound formulation inhibits ovarian cancer cell invasion and metastasis by down-regulating the protein expression of HIF-*α*, MMP-2, MMP-9, and E-cadherin, demonstrating significantly in vivo pharmacodynamic effects. These findings suggest a novel strategy for ovarian cancer treatment using EGF-targeted paclitaxel and emodin micelles [516]. Emodin-encapsulated stearic acid-g-chitosan oligosaccharide (CSO-SA/EMO) enhances antitumor potential of emodin through micelle delivery. CSO-SA/EMO exhibits distinct physicochemical characteristics and efficient cellular uptake in gastric cancer (MGC803 and BGC823) cells, enabling passive enhanced permeability and retention (EPR) targeting in vivo. Compared to free emodin, CSO-SA/EMO significantly inhibits tumor growth, arrests cell cycle, and exhibits substantial antitumor activity in vivo [513]. Emodin-loaded, Cy7-functionalized, polyethylene glycol (PEG)-coated Fe_3_O_4_ (Fe_3_O_4_-PEG-Cy7-Emodin) exhibits enhanced magnetic susceptibility, excellent biocompatibility, passive targeting to pancreatic cancer (BxPC-3 and hTERT-HPNE), capability for dual-modal FI/MR imaging, controlling drug loading and release, minimal side effects, and potent antitumor efficacy. These characteristic positions it as a promising drug delivery system for pancreatic cancer [444].

Hydrogels with different reactivity can enhance the targeting properties of emodin [515, 518]. Emodin encapsulated in pH/ion responsive self-assembling peptide (RADA16-I) hydrogels demonstrates enhanced immunotherapeutic potential by significantly inhibiting lung cancer (Lewis) cell survival, increasing the uptake, promoting cell apoptosis and inducing cell cycle arrest at G2/M phase, and reducing migration, invasion, and clonogenicity, while mitigating toxicity to normal cells [515]. Emodin encapsulated with glycyrrhizin in hybrid membrane (erythrocyte and macrophage to form a hybrid membrane)-coated nanoparticles enhanced solubility and antitumor efficacy, achieving an IC_50_ half that of free emodin. Photodynamic therapy using the hybrid membrane-derived nano-formulation enhanced ROS levels and promoted early death of B16 melanoma cells via the Bax/Bcl-2 pathway [517]. Therefore, emodin encapsulated in various nanocomposites, liposomes, and micelles significantly enhances cancer cell killing efficiency and exhibits potent anti-cancer activity. Targeted modifications improve uptake and efficacy, outperforming free emodin in inhibiting tumor growth and metastasis. Hydrogel encapsulation and hybrid membrane-coated nanoparticles further enhance targeting and solubility, with photodynamic therapy increasing ROS levels and inducing apoptosis.

In sum, emodin emerges as a multi-dimensional agent that aligns a range of cell death pathways (necroptosis, autophagy, and ferroptosis) with immune reprogramming to thwart tumor progression and metastasis. It tackles difficult challenges such as cancer recurrence and cachexia, reshapes the tumor microenvironment by modulating macrophages and neutrophils, and overcomes drug resistance via synergistic combinations with both conventional chemotherapeutics and molecularly targeted interventions. Through advanced delivery systems and omics-guided mechanistic insights, emodin exemplifies a sophisticated approach to cancer therapy that bridges robust biological activity with the promise of clinical translation.

### Epigallocatechin gallate (EGCG)

Epigallocatechin gallate (EGCG) is a polyphenolic compound that is extracted from *Camellia sinensis*, widely consumed in China and also across the globe [519]. EGCG has been demonstrated to possess multiple therapeutic benefits. For instance, it exhibits cardioprotective, hepatoprotective, and neuroprotective effects, and is characterized by antioxidant, anti-inflammatory, antibacterial, antiviral, antidepressant properties [520]. Furthermore, it exhibits a broad spectrum of anti-cancer activities against various types of cancers, such as lung, breast, and colon cancers. It exerts its anti-tumor effects through multiple mechanisms, which include the modulation of tumor immunity, reversal of drug resistance, and the induction of apoptosis [521]. Several studies have investigated the combination of EGCG with conventional anti-cancer treatments. These investigations aim to optimize the safety and effectiveness of chemoradiotherapy regimens while simultaneously reducing the associated side effects [522].Additionally, in silico and in vitro studies have provided evidence that EGCG targets NF-*κ*B pathway, highlighting its potential as a therapeutic alternative for pancreatic cancer treatment by reducing cell growth, inducing apoptosis, and repressing NF-*κ*B target genes [523]. Besides, an ex vivo study has been conducted to develop high-performance microgel assembly bioinks using EGCG-modified hyaluronic acid and phenylboronic acid grafted hyaluronic acid. The developed bioinks facilitate the high-fidelity 3D bioprinting of complex structures, owing to their excellent rheological properties. They also hold potential applications in drug testing, particularly when using breast cancer organoids [524]. In clinical study, EGCG is utilized in the context of colon cancer (NCT02891538), esophageal cancer (NCT05039983), breast cancer (NCT02580279), and lung cancer (NCT02577393) to evaluate its supportive and chemo preventive effects in cancer patients. The following section provides a concise summary of the intricate mechanisms and specific targets through which EGCG acts against various types of cancer.

### EGCG improves tumor immunity

EGCG has been shown to exert an impact on various immune cells, including neutrophils, macrophages, cytotoxic T-cell, and tumor-infiltrating lymphocytes (TILs), within the context of multiple cancer types such as colon, breast, bladder cancers, as well as melanoma, leukemia, and renal cell carcinoma [525, 526]. For instance, EGCG can stimulate T cell and NK cell activity, enhancing the function of Tregs to maintain immune tolerance and prevent autoimmune reactions in melanoma and colon cancer [527, 528]. Furthermore, when combined with the inhibitor fragment 1 (FRA), EGCG can directly target PD-L1 dimers exerting more effective anti-cancer immunity [529, 530]. Notably, EGCG reduces the accumulation of MDSCs by inhibiting the secretion of cytokines such as inducible nitric oxide synthase (iNOS), Nox2, NF-*κ*B, and STAT3. It also blocks ECM-receptor interactions and upregulates critical genes including *Cxcl3*, *Met*, *Col8a1*, *Oasl2*, and *Mmp12*. As a result, this leads to an increase in the proportion of CD4^+^ and CD8^+^ T cells in tissue and tumor sites in mice bearing 4T1 mammary tumors [531]. Overall, EGCG exerts a multifaceted influence on tumor immunity. It enhances the activity of immune cells like T cells and NK cells, promote immune tolerance through the regulation of Tregs, and reduces the accumulation of MDSCs while up-regulating critical immune genes. This property underscores its potential as a potent immunomodulatory agent in cancer therapy.

### EGCG reverses tumor drug resistance

EGCG has been shown to delay the onset of multidrug resistance and enhance the efficacy of anti-cancer therapies across multiple malignancies, such as lung, breast, leukemia, ovarian, head and neck, and skin cancers. It achieves this by altering P-gp, MRP, BCRP, and EGFR, which play crucial roles in oncological treatment [532–534]. Specifically, EC31 reverses drug resistance in breast cancer (LCC6/MDR), leukemia (P388/ADR and K-562/P-gp) and human oral epidermal cancer (KB-A1) cells by inhibiting P-gp-mediated drug efflux and restoring intracellular drug accumulation [535, 536]. Furthermore, EGCG augments the effectiveness of doxorubicin and paclitaxel in overcoming MDR in NSCLC (H1975), human oral epidermal cancer (KB-A1), and lung cancer (H460/PT) cells by inhibiting both the protein function and expression of P-gp [537–539]. Moreover, EGCG modulates some signaling pathways (e.g., Hedgehog and AMPK/AKT/MAPK pathways) in human rhabdomyosarcoma cells and NSCLC to overcome drug resistance [534, 540, 541]. Pivotally, the assembled EGCG and daunorubicin nanocomplex-mediated disassembly enhances the treatment effect by synergistically reversing chemoresistance in leukemia (HL-60 and HL-60/MX2) and lung cancer (A549) cells [542, 543]. Overall, EGCG represents a promising therapeutic agent for overcoming drug resistance in cancer treatment. Its ability to inhibit key drug efflux pumps, modulate resistance-related signaling pathways, and restore the effectiveness of standard chemotherapies highlights its potential as an adjuvant in cancer therapy.

### EGCG suppresses cancer progression via classical modalities

EGCG has been shown to regulate the cell cycle in various cancer types, including breast, prostate, lung, and colon cancers [544, 545]. Its primary mechanism of action involves inducing cell cycle arrest at different phases, thereby impeding cancer cell proliferation. Specifically, EGCG can trigger cell cycle arrest at the G2/M phase, preventing the transition to the S phase. Additionally, it is capable of inducing G2/M phase arrest, which disrupts mitotic progression and ultimately leads to apoptosis. For instance, in liver cancer cells (Hep G2 and Huh7), EGCG induces G2/M phase arrest by up-regulating the protein expression of p21 waf1/Cip1 and down-regulating cell cycle-related genes such as *CDC25A*, *CCNB*, *CCNB2*, and *CCND* [546, 547]. Studies have provided evidence that EGCG can block DNA replication in prostate cancer (DU 145) and colon cancer (HCT 116) cells through the targeting of multiple cell cycle regulators [548–552]. Moreover, EGCG induces G0/G1 phase arrest in oral squamous cell carcinoma (HSC-3) cells [553]. In gastric carcinoma (CRL-1739 and TSGH 9201) cells, EGCG induces G2/M arrest by activating the CDK1/cyclin B1 complex [554]. Notably, EGCG modulates WEE1 kinase, which is overexpressed in numerous cancers including lung, colon, breast, liver cancer, bladder, and brain cancers, and plays a crucial role in the G2/M DNA repair checkpoint [555–557]. Furthermore, EGCG suppresses key signaling pathways such as the PI3K/AKT/mTOR and AMPK pathways, and also inhibits factors like ROS, iNOS, Cox-2, NF-*κ*B, Senescence-associated secretory phenotype (SASP), and p53-mediated cell cycle regulation in preadipocytes [558]. In ovarian cancer (SK-OV-3 and OVCAR3) and esophageal squamous cell carcinoma (KYSE150 and KYSE510) cells, EGCG induces apoptosis by increasing the levels of pro-apoptotic proteins (Bax and Cytochrome c), activating Caspase-3 and Caspase-9, and stimulating tumor suppressor genes (*PTEN* and *SERPINB2*), while simultaneously decreasing the expression of anti-apoptotic proteins (Bcl-2 and Bcl-xL) [559, 560]. EGCG also demonstrates enhanced growth inhibition and apoptosis induction in SK-OV-3 cells, accompanied by the upregulation of PTEN protein expression [561, 562]. In human melanoma cell line A375, EGCG induces apoptosis via mitochondrial signaling pathway and downregulates autophagy through the AMPK/mTOR and PI3K/AKT/mTOR signaling pathway [563]. EGCG also disrupts the STAT3/CXCL8 signaling pathway, thereby inhibiting the formation of NETs and reducing motility and invasion in colon cancer (SW480) cells [564]. Moreover, it curtails VEGF-dependent angiogenic pathways and significantly reduces endoglin/pSmad1 levels, which inhibits tumor growth in human liver cancer (PMECs) and gastric cancer (SGC7901) cells and alleviates asthma symptoms [565–567]. Furthermore, EGCG inhibits TGF-*β*-induced EMT in human cervical cancer (Hela and SiHa) and anaplastic thyroid carcinoma (8505C) cells via the ROS/Smad signaling [568–570]. Additionally, in glioblastoma (U87), EGCG can inhibit the induction of EMT [570–572]. Overall, EGCG suppresses cancer progression through a diverse array of classical mechanisms, encompassing cell cycle arrest, apoptosis induction, inhibition of angiogenesis, and disruption of EMT. By precisely targeting key cell cycle regulators, apoptosis-related proteins, and signaling pathways that are integral to tumor progression and metastasis, EGCG exhibits significant potential as a therapeutic agent for various malignancies.

### Investigating EGCG anti-cancer effects through drug delivery system

Due to its inherent chemical instability, EGCG is frequently combined with metals or other substances to form nanoparticles, which enhances its applicability in cancer treatment [573, 574]. For example, MXene@EGCG can inhibit the expression of HSPs in tumor cells [575]. Moreover, EGCG-encapsulated micelles induce cancer cell apoptosis, trigger cell cycle arrest, and enhance the cellular uptake of EGCG in multiple cancer cell lines, including gastric cancer (BGC-823 and SGC-7901), colon cancer (HT-29 and HCT 116), breast cancer (MCF-7), and glioblastoma multiforme (GL261) cells [407, 412, 415, 416]. Additionally, EGCG-loaded liposomes have been demonstrated to enhance cytotoxicity compared to free EGCG in breast cancer (SKBR3 and MCF-7) and liver cancer (H22 and Hep G2) cells [420, 421]. These innovative carrier materials not only effectively overcome the limitations associated with the natural properties of EGCG but also significantly enhance its therapeutic efficacy against diverse forms of cancer, thereby representing a promising approach for improving cancer treatment outcomes.

### EGCG regulates cytotoxicity for its anti-cancer activity

The term "cytotoxic" refers to a cell that is eliminated by chemicals or other cells rather than necrosis or apoptosis. Cytotoxicity has been demonstrated to be correlated with EGCG's anti-cancer activity [576, 577]. In various cancer cell lines such as human pancreatic cancer (PANC-1, MIA PaCa-2, and BxPC-3), human colon cancer (SW480, HCT-15, and HT-29), and human lung cancer (HT1975, H358, and A549) cells, EGCG has been demonstrated to increase the cytotoxicity of chemotherapy drugs [578, 579]. Through mechanisms such as promoting the elevation of oxidative stress within diseased cells [580] and the enhancing cytotoxic T-cell responses, EGCG palmitate exhibits specific cytotoxicity towards cancer cells. At higher dosages (≥ 125 μg/mL), EGCG exhibits a cytotoxic effect, leading to the death of approximately 30% of J774A.1 cells while simultaneously inducing the production of substantial amounts of lysozyme and IL-1*β*, which can contribute to the reduction of inflammation [568, 581]. By exploiting the vulnerability of cancer cells to the accumulation of toxic metabolites, cytotoxicity mediated by EGCG can effectively prevent cancer cells from surviving under the burden of harmful byproducts. This approach holds the potential to precisely and effectively eradicate tumors while minimizing damage to healthy and non-cancerous cells. Future research on EGCG may focus on identifying and inhibiting enzymes that are responsible for eliminating harmful byproducts of cancer cell metabolism, thereby further enhancing its anti-cancer potential.

### EGCG exerts its anti-cancer effects through combining with other medications

Recent investigations into the synergistic effect of EGCG in combination with various agents have yielded significant findings. Specifically, when combined with monoclonal antibodies, first-line chemotherapy (such as doxorubicin, oxaliplatin, paclitaxel, 5-fluorouracil, and cisplatin), targeted drugs (including cetuximab, gefitinib, and erlotinib), immunotherapy drugs (like pembrolizumab and atezolizumab) and other natural products (e.g., curcumin), EGCG has demonstrated its potential in inhibiting multiple cancer types. For instance, studies have shown its inhibitory effects on pancreatic cancer (PANC-1), cervical cancer (HeLa and C-33A), and colon cancer (HCT 116) cells, primarily by reducing drug resistance and enhancing treatment sensitivity [582–585]. In addition, the combination of high-dose celecoxib and EGCG has been found to decrease the number of tumor cells induced by cisplatin [586]. Of note, it has been observed that EGCG in combination with other natural products exhibits enhanced efficacy in tumor treatment compared to its use alone [587–590]. For example, when cilantro is combined with EGCG, it can reduce the proliferating cell nuclear antigen at both mRNA and protein levels, thereby inhibiting the activity of human uterine fibroid cells [591]. Moreover, a conjugate composed of curcumin, EGCG and resveratrol has demonstrated a potent killing effect on head and neck cancer (TU212) cells [592]. In recent years, the combination of vitamins and EGCG has also played an important role in tumor treatment, especially in female patients [593, 594]. The ability of EGCG to synergize with a diverse range of therapeutic agents—spanning from conventional chemotherapies to targeted therapies, immunotherapies, and natural products, underlines its potential as an adjunct in cancer treatment. By mitigating drug resistance and augmenting treatment sensitivity, EGCG could potentially play a pivotal role in improving the outcomes of existing therapies. Its combinations with other natural products, such as curcumin, resveratrol, and cilantro, suggest that integrating EGCG into multi-modal treatment regimens may offer greater therapeutic benefits than using it as a standalone agent.

### Application of multi-omics techniques to investigate the anti-cancer properties of EGCG

Through the integration of transcriptomics and metabolomics investigations, it has been revealed that EGCG can interfere with glycerophospholipid metabolism and thereby inhibit the growth of colon cancer (HT-29) cells [595]. Metabolomics studies have further demonstrated that EGCG exerts anti-lung cancer benefits by regulating 33 differential metabolites in A549 cells, which are involved in various metabolic processes including glucometabolic, amino acid metabolism, nucleotide metabolism, and vitamin metabolism [596, 597]. Moreover, mRNA and miRNA library sequencing studies have indicated that EGCG specifically modifies mRNA and miRNA expression, leading to cell death in lung cancer and breast cancer (MCF-7) cells [598, 599]. However, the precise mechanism through which EGCG alters miRNA expression remains elusive, which presents an interesting area for further research [600]. By influencing key metabolic pathways, such as glycerophospholipid metabolism and glucometabolism, EGCG alters the metabolic profile of cancer cells, contributing to its anti-cancer properties. Additionally, EGCG’s capacity to modulate mRNA and miRNA expression highlights its potential in regulating gene expression networks that are implicated in cancer progression. While much has been uncovered, the exact mechanism by which EGCG influences miRNA expression still requires further in-depth investigation.

Overall, EGCG is notable for its capacity to modulate tumor immunity, overcome drug resistance, and orchestrate classical anti-cancer pathways, ranging from cell cycle arrest to apoptosis. By bridging multi-omics insights with advanced formulation techniques and synergistic regimens, EGCG exemplifies a promising model of how a single polyphenol can exert multifaceted control over tumor while minimizing the associated toxicities.

### Ginsenosides

Ginsenosides, characterized by their triterpenoid nature, constitute one of the active constituents within Ginseng radix et rhizoma or *Panax ginseng* (Renshen in Chinese) [601, 602]. Among these ginsenosides, ginsenoside Rg3, ginsenoside Rh2 and ginsenoside compound K have demonstrated potent and diverse pharmacological activities, particularly in the context of breast cancer treatment [603, 604]. Rg3 is a saponin compound with two distinct enantiomers: 20 (S)-ginsenoside Rg3 and 20 (R)-ginsenoside [605]. This compound exhibits a broad spectrum of pharmacological characteristics, among which its antioxidant and anti-inflammatory effects are especially prominent. Ginsenoside Rg3 is capable of diminishing lung inflammation, averting damage to liver and kidney functions, lessening neuroinflammation, averting cerebral and myocardial ischemia–reperfusion injury, and ameliorating symptoms of hypertension and diabetes [606]. Ginsenoside Rg3 has a wide range of anti-cancer properties, which are related to its functions in immune regulation, anti-angiogenesis, induction of mitochondrial autophagy, induction of tumor cell apoptosis, and inhibition of metastasis and invasion [605, 607]. The anti-inflammatory, antioxidant and anti-cancer properties of ginsenoside Rg3 have been validated through numerous in vivo and in vitro studies. It has been shown to be effective in the treatment of various diseases, including colon, breast, gastric, and liver cancers, as well as atherosclerosis, liver fibrosis, Huntington's disease, cardiac hypertrophy, heart failure and various skin diseases [608–612]. In silico analysis shows that ginsenoside Rg3 and ginsenoside Rh1 exert anticancer effects through different mechanisms: Ginsenoside Rg3 activates miR-424-5p by down-regulating ATXN8OS, while Rh1 inhibits metastasis by targeting RhoA and ROCK1, with both showing cytotoxic and pro-apoptotic effects in breast and lung cancers [613, 614]. And in vivo study shows that ginsenoside Rg1 exhibits strong cytotoxicity against lung cancer cells and suppresses oncogene expression in a zebrafish model [615]. In clinical trials, ginsenoside Rg3 is used to treat liver cancer (NCT01717066 and NCT04523467) and gastric cancer (NCT01757366). Ginsenoside Rh2, which is the predominant bio-active ginsenoside derived from the root of Panax ginseng, has been demonstrated to possess a diverse range of pharmacological activities [616]. These activities encompass antitumor effects, enhancement of cardiac function, mitigation of fibrosis, anti-inflammatory actions, antidepressant effects, and thus exhibit remarkable medicinal potential [617–621]. In cancer treatment, ginsenoside Rh2 shows multiple activities against tumor cells, such as influencing tumor immunity and autophagy [621, 622]. Compound K, which is one of the metabolites of ginsenoside Rb1, exhibits antioxidant, anti-inflammatory, anti-cancer, and hypolipidemic properties [623–625]. Compound K exerts an inhibitory effect on tumor growth through ferroptosis as well as the delay of cell cycle [626, 627]. The subsequent part presents an overview of the mechanisms and molecular targets through which ginsenoside exerts its actions against diverse forms of cancer.

### Ginsenosides improve tumor immunity

The anti-cancer efficacy of ginsenoside Rg3 is closely related to its immunomodulatory function. In clinical studies, ginsenoside Rg3 has been demonstrated to effectively increase the counts of CD3^+^ T, CD4^+^ T, and CD8^+^ T lymphocytes. It also enhances the activity of NK cells, thereby augmenting the treatment effectiveness for NSCLC patients [628]. In vivo experiments regarding gliomas (C6), Rg3 induces the transformation of TAMs from the protumor M2 phenotype to the antitumor M1 phenotype. This alteration activates the immune microenvironment, promotes the T cell immune response, elevates the M1/M2 ratio, and simultaneously reduces the proportion of Tregs and myeloid suppressive cells [629]. In tumor microenvironment, ginsenoside Rg3 exerts regulatory effects on immune inflammatory cells, cytokines, as well as relevant signaling pathways. Specifically, it has been demonstrated that ginsenoside Rg3 regulates STAT3 and suppresses the secretion of CCL2 by tumor cells. As a result, the recruitment of MDSCs and TAMs is decreased. This facilitates the immune system in recognizing circulating tumor cells, thereby impeding their colonization and proliferation [630]. Moreover, ginsenoside Rg3 also regulates TGF-*β*/Smad signaling pathway and inhibits the secretion of TGF-*β* by tumor cells. Consequently, cancer-related fibroblasts are inactivated and revert to a dormant state, which weakens the robustness of the matrix barrier [631]. Ginsenoside Rh2 also exhibits remarkable effects in the tumor microenvironment. In colon cancer (MC38) cells, it initiates the infiltration, multiplication and activation of T cells. For breast cancer (MDA-MB-231 and 4T1) cells, it enhances the immune surveillance of NK cells. In pancreatic cancer (Panc02 and PANC-1) cells, it promotes the infiltration of DCs [621, 632, 633]. Meanwhile, compound K can regulate the balance between Tregs and T helper cell in lung cancer (A549 and Lewis) cells, modulate the tumor microenvironment by means of targets, such as STAT3 and PD-L1 in prostate cancer (DU 145, PC-3, and LNCaP) cells [634, 635]. Thus, ginsenosides, including ginsenoside Rg3, ginsenoside Rh2, and compound K, significantly enhance tumor immunity through a variety of mechanisms, including the activation of T cells, modulation of macrophage phenotypes, regulation of immune pathways and cytokines, and reprogramming of the tumor microenvironment. By shifting immune cells towards more potent anti-cancer phenotypes, inhibiting immunosuppressive factors, and strengthening the immune system’s ability to detect and eliminate tumor cells, these compounds effectively promote anti-tumor immune responses.

### Ginsenosides reverse tumor drug resistance

Ginsenoside Rg3 has been the subject of extensive investigations regarding its potential to counteract the multidrug resistance of cancer cells. This encompasses the resistance of first-line chemotherapeutic agents such as paclitaxel, temozolomide, tamoxifen, osimertinib, gemcitabine, and icotinib in various malignancies including breast, lung, pancreatic cancers, as well as glioblastoma multiforme [636–638]. The resistance to paclitaxel, whether inherent or acquired during the course of treatment, represents a significant hurdle in breast cancer chemotherapy. Ginsenoside Rg3 exerts its effects in two aspects: it directly targets tumor cells and simultaneously participates in the remodeling of the tumor microenvironment. When combined with paclitaxel, this treatment suppresses the activation of the IL-6/STAT3 pathway. Moreover, it facilitates the transformation of the initial tumor M2 macrophages into an anti-tumor M1 type, suppresses MDSCs, reduces tumor related fibroblasts and collagen fibers in tumor microenvironment, promotes tumor cell apoptosis, and effectively reverses the MDR of breast cancer (MCF-7/PTX) xenograft model [639]. Tamoxifen is a commonly used first-line drug for clinical treatment of breast cancer. Enhanced glycolysis has been identified as a crucial factor contributing to tamoxifen resistance, and ginsenoside Rg3 can effectively address this issue. The presence of ginsenoside Rg3 leads to an elevation in the levels of 6-phosphofructose-2-kinase/fructose-2,6-bisphosphatase 3 (PFKFB3) in breast cancer (MCF-7/TamR and T-47D/TamR) cells [640]. Additionally, ginsenoside Rg3 also attenuate the resistance of NSCLC cells to osimertinib by suppression of the stemness that relies on the Hippo pathway [637]. In lung cancer cells, ginsenoside Rg3 inhibits ROS production, leading to NF-*κ*B and HIF-1*α*-mediated downregulation of PTX3, and inhibits gemcitabine induced MDR of lung cancer cells [638]. In glioma cells, 20 (S)-Rg3 effectively downregulates O6-methylguanine DNA-methyltransferase expression through the Wnt/*β*-catenin pathway, significantly reversing MDR [636]. In pancreatic cancer cells, ginsenoside Rg3 upregulates the expression levels of long noncoding RNA cancer susceptibility candidate 2 (*CASC2*) and *PTEN*, leading to the suppression and programmed cell death of pancreatic cancer cells that are resistant to gemcitabine [641]. Ginsenoside Rh2 also demonstrated significant effects in overcoming drug resistance. In pancreatic cancer (MIA PaCa-2 GR) bearing mice, it overcomes the resistance to gemcitabine by restraining LAMC2-Modulated ABC transporters. In paclitaxel-resistant breast cancer (MCF-7/PTX) cells, it suppresses cell proliferation by inhibiting the protein expression of P-gp [642]. Overall, ginsenosides, particularly ginsenoside Rg3 and ginsenoside Rh2, play a critical role in reversing tumor drug resistance through various mechanisms. By targeting key signaling pathways such as IL-6/STAT3, autophagy, glycolysis, and Wnt/*β*-catenin, as well as by modulating the tumor microenvironment, ginsenosides enhance the efficacy of chemotherapy drugs in cancer types that exhibit resistance, including breast, lung, pancreatic cancers, and glioblastoma. Their capacity to regulate drug resistance proteins, metabolic pathways, and immune cell phenotypes renders them promising candidates for combination therapies aimed at overcoming MDR and improving the outcomes of cancer treatment.

### Ginsenosides suppress cancer growth by regulating the processes of autophagy and ferroptosis

In the existing body of research, ginsenoside Rg3 has been shown to affect cellular autophagy, which constitutes one of the underlying mechanisms for its tumor suppressive effects. Specifically, studies have shown that 20 (S)-Rg3 blocks the late stage of autophagy (lysosomal fusion and autophagic lysosomal degradation) in cervical cancer cells, preventing them from maintaining a stable state through autophagy and inducing apoptosis in cervical cancer (HeLa) cells [643]. Moreover, ginsenoside Rg3 has been found to inhibit the protective autophagy of breast cancer (MCF-7) cells under hypoxic conditions [644]. In addition, in certain cancers, the tumor inhibitory effect of ginsenoside Rg3 is related to its ability to promote autophagy. For instance, ginsenoside Rg3 suppresses the growth, infiltration, and motility of osteosarcoma (MG-63 and U-2 OS) cells, while boosting their apoptosis and autophagy processes [645]. In human colon cancer cells, ginsenoside Rg3 induces mitochondrial autophagy. The action of ginsenoside Rg3 triggers the PINK1-Parkin signaling pathway, which leads to the recruitment of Parkin and ubiquitin proteins to the mitochondria, thereby initiating mitochondrial autophagy. During this process, GAPDH is involved and plays a role in regulating Parkin translocation to mitochondria [607]. Apart from autophagy, ginsenoside Rg3 influences ferroptosis, which is another form of programmed cell death. The dynamic nano-catalyst protected by ginsenoside Rg3 can activate apoptosis pathways, resulting in the elimination of pancreatic cancer (L3.6pl) cells [646]. However, ginsenoside Rh2 inhibits autophagy and promotes apoptosis of cervical cancer (HEK 293 T, HeLa, and C-33 A) cells under starvation conditions [647]. Meanwhile, ginsenoside compound K triggers the apoptosis of human cervical cancer (HeLa) cells by modulating autophagy, and induces ferroptosis through the FOXO pathway in liver cancer (Hep G2 and SK-HEP-1) cells [648]. Overall, ginsenosides, particularly ginsenoside Rg3, ginsenoside Rh2, and compound K, exhibit versatile and potent anti-cancer effects through the regulation of autophagy and ferroptosis. These compounds can either promote or inhibit autophagy depending on the specific cancer type and cellular conditions, such as the presence of hypoxia or nutrient starvation.

### Ginsenosides suppress cancer progression via classical modalities

In terms of classic pathways for anti-cancer, ginsenoside Rg3 triggers a halt in the cell cycle across different cancer types. A common manifestation is that ginsenoside Rg3 increases the ratio of cancer cells at the G0/G1 phase. In detail, research has revealed that ginsenoside Rg3 disrupts the cell cycle at G0/G1 phase via the EGFR/Ras/Raf/MEK/ERK pathway in lung cancer (A549), liver cancer (Hep G2) cells, and the formation of Cyclin D1, CDK2, and CDK4 in colon cancer (SW620 and LoVo) cells [649, 650]. Furthermore, ginsenoside Rg3 interferes with the G1/S transition in prostate cancer (PC-3) cells by modulating ROS [651], triggers a halt in the melanoma (B16) cell cycle during the S phase, and reduces the levels of the proliferating cell nuclear antigen [652]. In lung cancer (A549 and NCI-H1299) cells, ginsenoside Rg3 increases the ratio of cells at G2/M phase [653]. Similarly, ginsenoside Rh2 induces cell cycle arrest at G0/G1 phase in oral cancer (YD10B and Ca9-22), colon cancer (HCT-15, HCT 116, and DLD-1) and melanoma (A375 and B16-F10) cells [654–656]. Compound K blocks the G0/G1 phase in liver cancer (Hep G2, SMMC-7721, BEl-7404, and Huh7) and cervical cancer (HeLa) cells [648, 657].

Ginsenoside Rg3 triggers cell death through apoptosis in a range of cancer cells, which involves the regulation of various proteins by ginsenoside Rg3. For example, ginsenoside Rg3 triggers cell death and suppresses cell growth by reducing TIGAR protein levels in rat gastric precancerous areas [658]. In NSCLC cells, ginsenoside Rg3 may increase Bax level and activate Caspase-3 in cells and decrease the expression of the anti-apoptotic protein (Bcl-2) [659]. Caspase-3 is a commonly studied pro-apoptotic protein in the context of the pro-apoptotic mechanism of ginsenoside Rg3. When administered in combination with 5-fluorouracil, ginsenoside Rg3 can significantly increase the apoptosis rate in colon cancer (SW620 and LoVo) cells through the activation of the Apaf1/Caspase-9/Caspase-3 pathway [650]. In addition, the pro-apoptotic effect of ginsenoside Rg3 on cancer cells has also been studied in models of liver, ovarian, gastric cancers, as well as renal cell carcinoma and melanoma [660–664]. Through *miR-429*, Rg3 regulates genes associated with apoptosis in gastric cancer (AGSR-CDPP) cells resistant to cisplatin [662], regulates cancer cell apoptosis via the MEK signaling pathway in melanoma (A375.S2) cells [663], and promotes p53 demethylation and histone acetylation of p21 and p16 to enhance apoptosis of renal cell carcinoma cells [664]. Ginsenoside Rh2 can affect the expression of apoptotic proteins such as Bcl-2 in osteosarcoma (U-2 OS) and esophageal cancer (ECA109 and TE-13) cells, thereby inhibiting tumor progression [665, 666]. In addition, compound K can promote the activities of Caspase-3 and Caspase-9 in prostate cancer (PC-3) cells [667].

Ginsenoside Rg3 primarily inhibits cell migration by reducing the protein levels of MMPs such as MMP-2, MMP-7, and MMP-9, and by obstructing the EMT process in cancer cells [652, 668–670]. Specifically, ginsenoside Rg3 inhibits Notch-Hes1-EMT signaling to suppress the cell migration and metastasis in colon cancer [668]. In osteosarcoma cells, the mechanism underlying ginsenoside Rg3's inhibition of migration is also related to the Wnt/*β*-catenin pathway [669]. Furthermore, ginsenoside Rg3 plays a key role in preventing liver cancer cells from migrating and invading by increasing the protein levels of ARHGAP9 [671]. Ginsenoside Rh2 inhibits tumor growth via ER*β*-TNF*α* pathway in breast cancer (MCF-7) cells [672]. Compound K inhibits the proliferation and invasion of esophageal cancer (ECA109) cells through the VEGF-A/PI3K/AKT pathway [625]. Therefore, these compounds modulate key signaling pathways such as EGFR/Ras/Raf/MEK/ERK, ROS, MEK, and PI3K/AKT, thereby exerting control over cancer cell proliferation, survival, and metastasis.

### Ginsenosides combined with other medications for cancer treatment

The combined application of PD-L1 has demonstrated notable anti-cancer outcomes, which can be attributed to the activation of memory T cells and the reduction in adaptive PD-L1 enrichment within breast cancer (4T1) cells [673]. At present, ginsenoside Rg3, recognized for its multifunctional anti-tumor properties, has also received attention for its combined application with first-line anti-cancer drugs. When ginsenoside Rg3 is combined with 5-fluorouracil, a significant enhancement the suppression of multiple aspects related to cancer progression, including proliferation, invasion, migration, and angiogenesis, is observed in both lung and colon malignancies. In lung cancer (A549 and SPC-A-1) cells, this combination achieves its effects through the suppression of the NF-*κ*B signaling pathway's functionality and the downregulation of VEGFA expression [674]. In colon cancer (SW620 and LoVo) cells, the dual treatment of ginsenoside Rg3 and 5-fluorouracil triggers the Apaf1/Caspase-9/Caspase-3 pathway, leading to a marked increase in apoptosis, elevating the levels of Cyclin D1, CDK2, and CDK4 levels to halt G0/G1 cell cycle, and suppresses the PI3K/AKT signaling pathway [650]. Within liver cancer (SMMC-7721) cells, ginsenoside Rg3 potentiates oxaliplatin's anti-cancer properties, curbing liver cancer growth and facilitating cell death through the control of PCNA and Cyclin D1 expression [675]. Ginsenoside Rg3 combined with sorafenib exhibits a synergistic effect. Administering ginsenoside Rg3 and sorafenib together markedly reduces the survival, glucose uptake, and lactate concentrations in liver cancer (Hep G2 and BEL7404) cells, a process linked to the glycolysis mediated by HK2 and the PI3K/AKT signaling pathway [676]. Ginsenoside Rg3 is also capable of augmenting the cancer-fighting properties of gefitinib and doxorubicin. In NSCLC (A549 and H1299) cells, ginsenoside Rg3 enhances gefitinib's cytotoxic effect, with variations depending on the dosage and treatment duration. Moreover, it significantly inhibits the migration of these cells [659]. When combined with doxorubicin, ginsenoside Rg3 suppresses tumor expansion and lung cancer metastasis in an osteosarcoma (143B and U-2 OS) xenograft model. This is achieved through their combined ability to regulate mTOR/HIF-1*α*/VEGF and EMT signaling pathways [677]. In addition, ginsenoside Rg3 can also be used in combination with certain active natural products to achieve a more potent anti-cancer effect. For instance, 20 (S)-Rg3 and curcumin suppress the proliferation of breast cancer (MDA-MB-231) cells in a dose- and time-dependent manner and enhance the radiosensitivity of cancer cells [678]. Ginsenoside Rh2 enhances anti-PD-L1 immunotherapy by promoting the infiltration, proliferation, and activation of CD8^+^ T cells in colon cancer (MC38) cells [621]. Ginsenoside Rh2 combined with EGFR-targeted liposomes can inhibit tumor growth and metastasis in breast cancer [679].Thus, the combination of ginsenosides with other therapeutic agents, including chemotherapy drugs, targeted therapies, natural products, and immunotherapies, significantly enhances their anti-cancer effects. These combinations work through various mechanisms, such as modulation of signaling pathways (e.g., NF-*κ*B, PI3K/AKT, and mTOR/HIF-1*α*/VEGF), induction of apoptosis, inhibition of metastasis, and enhancement of immune responses.

### Ginsenosides exert its anti-cancer effects through regulating gut microbiota

The occurrence and progression of cancer are associated with an imbalanced gut microbiota. For instance, a nanoparticle coupling of ginsenoside Rg3 reshapes the imbalanced gut microbiota in vivo and interferes with metabolism processes, thereby inhibiting the occurrence and metastasis of liver cancer [680]. Although there are reports in the literature regarding the regulatory effects of ginsenoside Rh2 and compound K on gut microbiota, the research focusing on their specific roles in cancer remains relatively limited [681–683].

### Investigating ginsenosides anti-cancer effects through drug delivery system

Ginsenosides inherently possess limitations in terms of bioavailability and typically require special delivery systems to be developed with other carriers to function effectively. The process of encapsulating Rg3 within PLGA nanoparticles (Ginsenoside Rg3-PLGA) and enveloping them in a membrane made of microcapsules from tumor cells results in Ginsenoside Rg3-PLGA@TMVs, which is notably capable of stimulating CD86 and CD80 DCs in a laboratory setting [684]. In comparison to cholesterol liposomes (C-LPs), ginsenoside Rg3-LPs not only markedly enhance cellular uptake and the capacity to penetrate glioma spheroids (C6) cells, but also significantly strengthen the active targeting of glioma and the intertumoral diffusion ability in vivo [629]. The self-micro emulsifying drug delivery system (RGO-SMEDDS) incorporating ginsenoside Rg3, ganoderma lucidum polysaccharides, and oridonin A restores immune functions by suppressing immunosuppressive cytokines and M2 polarized macrophages. Moreover, it can reduce angiogenesis through the downregulation of vascular endothelial growth factor and its receptors, and delay cell growth by obstructing the EGFR/AKT/protein kinase B/GSK3 signaling pathway in liver cancer (Huh7 and Hep G2) cells [685]. A ginsenoside Rg3-based nanoparticle formulated with folate, polyethylene glycol, and cyclodextrin has been validated to induce the transformation of immunosuppressive tumor microenvironment [608]. Polyethylene glycol-ginsenoside ginsenoside Rh1 and ginsenoside Rh2 conjugates as well as self-assembled micelles loading with ginsenoside Rh2 enhance water solubility and enable passive targeted delivery in lung cancer (A549) cells [686, 687]. Ginsenoside Rh2-irrigated nano-in-thermogel can also reduce the multidrug resistance of breast cancer (MCF-7/PTX) cells [688]. Micelles modified with homing peptides derived from chitosan, gold nanoparticles and multifunctional liposomes can effectively deliver compound K to cancer cells, thereby enhancing the anti-cancer effects [689–691]. Overall, the development of advanced drug delivery systems (DDS) is crucial for optimizing the therapeutic potential of ginsenosides in cancer treatment. Encapsulation within nanoparticles, liposomes, self-emulsifying systems, and conjugation with targeting ligands such as folate and PEG significantly enhance the bioavailability, solubility, and targeted delivery of ginsenosides to tumor sites. These systems not only improve the anti-cancer efficacy of ginsenosides but also assist in overcoming challenges like poor solubility and multidrug resistance. The combination of DDS with ginsenosides represents a promising strategy for enhancing the outcomes of cancer treatment.

In summary, ginsenosides exert multifaceted anti-cancer effects through various mechanisms. They modulate immune cell polarization, reverse drug resistance by targeting key signaling and metabolic pathways, and regulate cell death processes such as apoptosis, autophagy, and ferroptosis. Ginsenosides can also reshape the tumor microenvironment through remodeling of TAMs and TGF-β–related fibroblast activation. By integrating ginsenosides into advanced DDS, researchers have successfully overcome challenges like low bioavailability and multidrug resistance, thereby amplifying therapeutic outcomes. These attributes position ginsenosides as versatile candidates for combination therapies, leveraging immunomodulatory, metabolic, and signaling crosstalk to elicit robust anti-tumor responses across multiple malignancies.

### Icariin/icaritin

Icariin is one of flavonoids derived from Epimedii folium or *Epimedium brevicornu* (Yinyanghuo in Chinese) [692, 693]. Icaritin, a bioactive metabolite of icariin hydroxylated at the 3,7-positions, is the main metabolite of icariin in the body. Both icariin and icaritin are used for the treatment of sexual dysfunction, osteoporosis, Alzheimer's disease, and exhibit anti-inflammatory and anti-cancer effects [694, 695]. They demonstrate powerful inhibitory effects on many cancers such as liver, prostate, breast, cervical, breast, pancreatic cancers, as well as leukemia and melanoma [696–699]. Icariin and icaritin achieve their anti-cancer effects by inducing autophagy and apoptosis, disrupting the cell cycle, modulating tumor immunity, and inhibiting cell invasion and metastasis. To improve extraction rates, whole-cell catalysis demonstrates superior production performance, with Aspergillus sp.y 848 being used to isolate purer icaritin [700, 701]. Additionally, the combination of icaritin with chemotherapy drugs can inhibit NK cell or T cell lymphoma growth in vivo [702]. In China, combination therapies involving icaritin are the subject of numerous ongoing clinical trials for the treatment of hepatocellular cancer (NCT03236636, NCT03236649, ChiCTR2300077991, and ChiCTR2300077987) and pancreatic ductal adenocarcinoma (ChiCTR2300077907). It is noteworthy that the China National Medical Products Administration has granted approval for the use of icaritin (trade name acoradine) as a treatment option for patients with advanced liver cancer, emphasizing its safety, efficacy, and broad therapeutic potential in 2022 [703]. In summary, icariin and icaritin possess a wide range of pharmacological effects and show great promise in future anti-cancer treatments.

### Icariin and icaritin improve tumor immunity

Icariin and icaritin both enhance the anti-cancer response by modulating the function of CD4^+^ and CD8^+^ T cells, macrophages, and MDSCs in melanoma, breast, prostate, pancreatic and liver cancers [698, 704, 705]. Icariin increases the proportion of tumor-infiltrating CD4^+^ and CD8^+^ T cells, reduces the number of MDSCs, and induces their differentiation into macrophages and DCs, restores CD8^+^ T cell function in 4T1 breast cancer xenograft model in vivo [697, 706]. Furthermore, icariin exhibits dual anti-cancer mechanisms by decreasing polymorphonuclear-MDSCs and inhibiting macrophage differentiation from the M1 to M2 phenotype in pancreatic cancer (Panc02) cells [698, 704]. However, icaritin suppresses liver cancer by reducing hematopoietic stem cells and PMN-MDSCs in liver cancer (Hepa1-6) cells and exerts anti-cancer effects by reducing the proportion of MDSCs and restoring CD8^+^ T cell function in breast cancer (4T1) cells as well [706, 707].

Icariin enhances macrophage phagocytosis, reduces spleen and thymus indices, downregulates pro-inflammatory cytokines (IL-6, IL-8, IL-1*β*) in cervical cancer (SiHa) cells, and inhibits TGF-*β*1 protein expression in breast cancer (MDA-MB-231) cells, suggesting its immunomodulatory effects [708, 709]. In contrast, icaritin inhibits the release of TNF-*α* and IL-6 in lung cancer (A549) cells, preventing bone metastasis [710]. Icaritin downregulates the mRNA and protein expression of PD-L1 in liver cancer (SMMC-7721) and melanoma (B16-F10) cells, enhancing anti-cancer immune responses and prolonging patient survival [699, 711, 712]. In addition, icaritin upregulates CXC chemokines (e.g., CXCL9 and CXCL10) and T cell marker factors (e.g., CD8 and IFN-*γ*), facilitating the recruitment of immune cells to tumors, and formed a "hot" tumor microenvironment in lung cancer (Lewis) cells [713]. Therefore, icariin and icaritin promote the infiltration of immune cells, inhibit various immune checkpoints, reduce the levels of inflammatory cytokines and enhance anti-cancer immunity.

### Icariin and icaritin reverse tumor drug resistance

Icariin and icaritin enhance the sensitivity of resistant liver, breast, ovarian, and osteosarcoma to first-line chemotherapy drugs (doxorubicin, tamoxifen and cisplatin) by inhibiting P-gp, MDR1 or MRP1 [714, 715]. Icariin specifically improves breast cancer (MCF-7/ADR and MCF-7/TAM) cells response to adriamycin and tamoxifen by blocking P-gp and autophagy [714, 716]. In contrast, icaritin targets MDR1 and downregulates the protein expression of P-gp in liver cancer (Hep G2/ADR) cells, meanwhile, it decreases the mRNA and protein levels of MDR1 and MRP1 in osteosarcoma (MG-63/DOX) cells to sensitize them to adriamycin and doxorubicin, respectively [717, 718]. Therefore, icariin and icaritin hold significant promise in overcoming tumor drug resistance by targeting and inhibiting key drug efflux pumps, including P-gp, MDR1, and MRP1. These compounds enhance the sensitivity of cancer cells to first-line chemotherapy drugs, such as adriamycin, tamoxifen, and cisplatin, by blocking the mechanisms that facilitate drug resistance.

### Icariin and icaritin suppress cancer growth by regulating the processes of autophagy

Both icariin and icaritin can prevent breast, liver, cervical, colon and oral squamous cancer through autophagy [696, 719]. Icariin induces the emergence of autophagosomes and modulates autophagy-related proteins (e.g., p62, Beclin-1, LC3-I, and LC3-II) in breast cancer (MDA-MB-468 and 4T1), cervical cancer (Hela) and ovarian cancer (SKVCR) cells [720–722]. Aside from autophagic vesicles, icaritin degrades mitochondrial proteins (e.g., MNF1, MNF2, HSP60, TOM20, and TiM23) in liver cancer (SK-Hep1) cells [696]. It also causes the accumulation of endogenous LC3 dots, modulates autophagy-related proteins, and subsequently increases autophagy flux in breast cancer (MDA-MB-468 and MCF-7), oral squamous cell carcinoma (CAL 27 and SCC-9) and liver cancer (Hepa1-6 and Huh7) cells [723–725]. Thus, icariin and icaritin exert anti-cancer effects by regulating the process of autophagy in various tumor types. Icariin enhances autophagy by inducing autophagosome formation and modulating key autophagy-related proteins, leading to the inhibition of cancer cell proliferation and promoting cell death. On the other hand, icaritin promotes mitochondrial autophagy and increases autophagy flux, inducing cell death in cancer cells.

### Icariin and icaritin suppress cancer progression via classical modalities

Icariin and icaritin have been identified to induce cell cycle arrest at G0/G1, S, and G2/M phases in breast, liver, prostate, ovarian, lung, colon, retinal pigment epithelial, nasopharyngeal, gallbladder, nasopharynx, multiple myeloma and NK/T-cell lymphoma [715]. Icariin triggers cell cycle arrest at the G0/G1 phase in multiple cancer types, including ovarian cancer (SK-OV-3), lung cancer (A549), cervical cancer (SiHa), breast cancer (MDA-MB-468 and 4T1), retinal pigment epithelial and gallbladder cancer (GBC-SD) cells [708, 721, 726–729]. Meanwhile, icariin downregulates the protein expression of Cyclin D1, further inducing G1/S phase arrest in ovarian cancer (SK-OV-3) cells [730]. In contrast, icaritin upregulates the protein expression of p21, causing in G0/G1 arrest in liver cancer (Hep G2 and Huh7) cells [731], induces G0/G1 or S phase arrest via regulating cell cycle markers (p27^Kip1^, p16^Ink4a^, and pRb) in prostate cancer (PC-3), endometrial carcinoma (RL95-2 and Ishikawa), nasopharyngeal carcinoma (HONE1 and HNE1) and multiple myeloma (U266), liver cancer (PLC/PRF/5) and colon cancer (HT-29) cells [732–734]. Additionally, icaritin demonstrates inhibitory effects on cell cycle arrest at G2/M phase in endonodal NK/T-cell lymphoma (SNT-8 and SNK-10) cells and cervical cancer (Hela) cells [735, 736]. Icariin and icaritin induce apoptosis in multiple cancers, such as breast, liver, ovarian, lung, colon, cervical, neuroblastoma and NK/T-cell lymphoma by enhancing pro-apoptotic proteins and reducing anti-apoptotic proteins [697, 708, 735–745]. Icariin activates Caspase-3, Caspase -9, PARP, DR4 and DR5, inducing apoptosis through the death receptor pathway in colon adenocarcinoma (HCT 116) cells [745]. It also modulates mitochondrial proteins to trigger apoptosis in lung adenocarcinoma (A549 and H1975) cells [741]. Additionally, icaritin demonstrates pro-apoptotic effects in extranodal NK/T-cell lymphoma (SNT-8 and SNT-10), cervical cancer (Hela), liver cancer (Hep G2) cells and neuroblastoma (TDP-43-transfected SH-SY5Y) cells [735, 736, 742–744]. In order to trigger apoptosis through the mitochondrial pathway, icariin and icaritin can both release Cytochrome C from mitochondria, triggering the Caspase cascade. Moreover, they activate death receptors (e.g., TNFR-1, DR4, and DR5), and Caspase-8, thereby initiating apoptosis via the death receptor pathway. Icariin suppresses migration and invasion in breast cancer (4T1, Hs 578 T, and MDA-MB-468), lung cancer (A549), and ovarian cancer (SK-OV-3) cells [721, 726, 739, 746]. It upregulates E-cadherin and downregulates Vimentin, inhibiting cell migration and invasion of cervical cancer (SiHa) cells [708]. Besides, icariin inhibits osteoclast generation and significantly suppresses the bone metastasis of prostate cancer (RM-1) cells [704]. Similarly, icaritin modulates metastasis-related proteins (e.g., MMP-9, E‐cadherin, N‐cadherin, and Vimentin) and exhibits inhibitory effects in multiple myeloma (RPMI 8226 and OPM-2), ovarian cancer (A2780), esophageal cancer stem cells (ECA109) and endometrial cancer (RL95-2 and Ishikawa) cells [732, 747–749]. Icariin and icaritin exert potent anti-cancer effects through classical mechanisms, including regulation of the cell cycle, induction of apoptosis, and inhibition of migration and invasion. By modulating key proteins involved in these processes, such as cyclins, Caspases, death receptors, and metastasis-related proteins, both compounds significantly suppress tumor growth and metastasis across a range of cancer types.

### Icariin and icaritin combined with other medications for cancer treatment

Studies have shown that combinations of icariin and icaritin can enhance the treatment of prostate, liver, breast, and nasopharyngeal cancers by boosting efficacy and reducing side effects [750–752]. The icariin and P815AB peptide combination notably enhances the activity index of CTLs in mastocytoma (P815)-bearing mouse model, boosts CD8^+^ T cell cytotoxicity against prostate cancer (PC-3, DU 145, and RM-1) cells, upregulates immune-related proteins for lymphoma protection, and inhibits autophagy in ovarian cancer (SKVCR) cells, sensitizing it to cisplatin [720, 753]. Moreover, icaritin, combined with anti-PD-1 antibodies notably amplifies the immune response and suppresses melanoma (B16-F10 and B16) and liver cancer (Hepa1-6) bearing models in vivo [699, 707]. Icaritin also synergizes with doxorubicin to induce immunogenic cell death in liver cancer (Hepa1-6) cells, enhances the anti-cancer immune response with unmethylated cytosine-guanine oligodeoxynucleotide in melanoma (B16-F10), overcomes drug resistance in breast cancer (4T1) cells with epigenetic drugs (JQ1), and induces ferroptosis in nasopharynx cancer (HONE1 and HNE1) cells with gamma irradiation [724, 752, 754, 755]. Thus, the combination of icariin and icaritin with other medications or treatment modalities significantly enhances the immune system, sensitize tumors to chemotherapy, overcome drug resistance, and promote cell death through mechanisms such as autophagy inhibition, immunogenic cell death, and ferroptosis.

### Icariin and icaritin derivatives inhibit cancer progression

Icariin can be rapidly metabolized in the body into a variety of active ingredients like icariside I and icariside II, making impressive progress in treating a wide range of bladder, lung, prostate and breast cancers [756–759]. Icariside I enhances beneficial gut bacteria, upregulates immune cells, and inhibits melanoma (B16-F10) cell growth [760, 761]. In contrast, icariside II targets various cancer types, including bladder cancer (BIU-87 and T24), cervical cancer (Hela), lung cancer (A549 and Lewis), prostate cancer (DU 145), colon cancer (SW620), and melanoma (A2058 and A375R) cells resistant to BRAF inhibitors, by blocking cell cycles, inducing autophagy and apoptosis, and modulating the tumor microenvironment when combined with anti-PD-1 [756–758, 762–766]. Notably, icariside II also induces ferroptosis in renal cell carcinoma (ACHN and Caki-1) and promotes differentiation in acute myeloid leukemia (HL-60 and THP-1) cells [767, 768]. Distinct from icariin, icaritin and its derivatives demonstrate unique anti-cancer mechanisms. Icaritin derivative IC2 triggers autophagy and apoptosis by targeting the SCD1 enzyme in breast cancer (MCF-7) cells in vitro and in vivo [759, 769]. Additionally, the modified icaritin isopentenyl derivative C3 induces cell apoptosis and cell cycle arrest at S phase in multiple myeloma (RPMI 8226), while another semi-synthetic derivative 11c inhibits the proliferation of liver cancer (Hep G2 and SMMC-7721) cells by inducing cell apoptosis and cell cycle arrest at G0/G1 phase [770, 771]. Therefore, icariin, icariside I, icariside II, and icaritin derivatives demonstrate broad-spectrum anti-cancer activity particularly effective in overcoming drug resistance and enhancing the therapeutic outcomes of existing treatments. Icariside II, in particular, shows promise in combination with immunotherapies, such as anti-PD-1, while other derivatives, such as IC2, C3, and 11c, offer new strategies for targeting specific cancer pathways, such as lipid metabolism, the cell cycle, and apoptosis.

### Icariin and icaritin exert anti-cancer effects through regulating gut microbiota

Although its entire potential in cancer treatment has not yet been investigated, it has been discovered that icariin regulates the human intestinal microbiota. Specifically, icariin treatment group leads to a significant increase in beneficial bacteria (e.g., bifidobacterium and lactobacillus) counts, while decreasing harmful bacteria (e.g., enterococcus and enterobacteriaceae) counts, thereby improving the intestinal flora imbalance of cervical cancer (U14) bearing mice [708].

### Application of multi-omics techniques to investigate the anti-cancer properties of icariin and icaritin

Transcriptomic analysis reveals distinct roles for icariin and icaritin in cancer regulation. Icariin downregulates NF-*κ*B-related genes in breast cancer (MDA-MB-231) cells [697]. Icariin emerges as a pivotal anti-cancer agent in liver cancer (Hep G2 and SMMC-7721) cells, with metabolomics suggesting arachidonic acid and other metabolites may contribute to its mechanisms [772]. Meanwhile, icaritin regulates PLK1 and induces apoptosis in NK or T-cell lymphoma (SNT-8, SNK-10, and NK-92 MI) cells, while activating the PI3K/AKT pathway to inhibit endometrial cancer (HEC-1-A) cell proliferation [702, 773]. Proteomic studies identify 1024 differential proteins enriched in autophagy and mitochondrial apoptosis pathways in liver cancer (SK-Hep1) cells [696]. Transcriptomic and metabolomic results show that icaritin increases white blood cells, alleviating cancer-induced leukopenia [774]. Pharmaco-omics and proteomics indicate that icaritin exerts its anti-lung cancer (Hep G2) cells by up-regulating FYN protein [775]. Therefore, the integration of multi-omics techniques has significantly advanced the understanding of the anti-cancer mechanisms of icariin and icaritin.

### Investigating icariin and icaritin anti-cancer effects through drug delivery system

The utilization of various delivery platforms, including phospholipid complexes, cyclodextrin complexes, micellar formations, nano-carriers, and solid materials, significantly enhance the bioavailability of icariin and icaritin, exhibiting remarkable potential in the treatment of breast, lung and gastric cancers [776–778]. Nanomaterials coated with icariin facilitated drug release, induce apoptosis, and show strong cytotoxicity against ovarian cancer (SK-OV-3 and Caov-3) cells [776]. A novel gelatin/polylactic acid (PLA) coaxial fiber membrane containing icariin enhances osteogenic effect and effectively suppresses the activity of human osteosarcoma (MG-63) cells [779]. The IRGD-functionalized RBCM-mimicking nanoparticles carrying icariin exhibits potent inhibitory effects on lung cancer (A549) cell proliferation [777]. Furthermore, nano-micelles formulated with icariin, curcumin and carrier material notably suppress the proliferation of breast cancer (MCF-7) cells in vitro and in vivo [780]. The optimized icariin-loaded bilosome-melittin (ICA-BM) formulation markedly enhances the anti-pancreatic cancer efficacy of icariin in PANC-1 cells [781]. In contrast, icaritin-based nanoparticle DDS demonstrate significant substantial promise in cancer treatment. For instance, the system FLA@GNPs induces apoptosis in lung cancer (A549) cells [782]. Co-loaded with icaritin and JQ1, within ARNP nanoparticles, the system inhibits breast cancer (4T1) cells and alleviates bone and lung metastasis [755]. Icaritin-laden micelles efficiently deliver the drug to oral squamous cell carcinoma (CAL 27) cells, inducing apoptosis and inhibiting proliferation in liver cancer (HCCLM3) in vitro and in vivo [783, 784]. Micelles containing icaritin and doxorubicin reshape the immune microenvironment and hinder the growth of liver cancer (Huh7 and Hepa1-6) cells [724]. Notably, the heat-sensitive IC-ML complex, supported by icaritin and Coix seed oil, triggers cell apoptosis and cycle arrest, enhancing the therapeutic efficacy against liver cancer (Hep G2) in vitro and in vivo [785]. Furthermore, PLGA@icaritin activates anti-cancer immune response, elicits immunogenic cell death in gastric cancer (MFC) cells [778]. The development of advanced DDS for icariin and icaritin has significantly improved the bioavailability, targeting, and therapeutic efficacy of these compounds in cancer treatment. By utilizing various platforms such as nanomaterials, micelles, and nanoparticles, these delivery systems enhance the controlled release of icariin and icaritin, promote apoptosis, and increase cytotoxicity in a range of cancers.

In sum, Icariin and its metabolite icaritin offer multiple anti-tumor mechanisms, including immunomodulation, drug resistance reversal, induction of autophagy and apoptosis, and cell cycle arrest. Icariin can also synergize with chemotherapy agents or immunotherapies to enhance efficacy and reduce toxicity. Across various malignancies—breast, liver, cervical cancers, and more—icariin and icaritin demonstrate a notable ability to inhibit tumor proliferation and metastasis. Advanced drug delivery platforms (e.g., nanoparticles, microcapsules, and microspheres) further improve their solubility and bioavailability. Additionally, derived compounds such as icariside I/II excel at triggering ferroptosis and boosting immune responses, while multiple ongoing clinical trials (particularly for liver cancer) continue to confirm their safety and therapeutic promise.

### Resveratrol

Resveratrol, a naturally produced polyphenolic molecule, has garnered considerable amounts of interest in recent decades due to its possible medicinal uses [786]. Resveratrol can be extracted from more than 70 plant species and is particularly abundant in grape, blueberry and raspberry, as well as Polygoni cuspidati rhizoma et radix (Huzhang in Chinese) [787], and several synthetic routes are also available to provide high quality and yield of resveratrol [788, 789]. Resveratrol is known for its antioxidant, anti-inflammatory and cardioprotective properties [790–792] and has a wide range of anti-cancer properties in oral, gastric, liver, pancreatic, breast, prostate, colon, lung, and skin cancers [793]. Resveratrol enhances tumor immunity, reverses tumor drug resistance, and regulates autophagy and cell cycle [794–796]. It can be used as a chemo preventive agent as well as a therapeutic agent involved in inhibiting the developmental stages of tumors [797, 798]. Thus, resveratrol can function as a complementary drug to conventional chemotherapy [799]. Besides, it can also be used as a combination of other therapies, acting synergistically with other anti-cancer drugs [800, 801]. In silico analysis investigates the binding of resveratrol to tumorigenic proteins, providing the first in silico characterization of its interactions with HIF-1*α*, and offering detailed insights into their structural configurations and potential therapeutic roles in NSCLC and pancreatic cancers [802, 803]. Additionally, the organoid-based ex vivo model is emerging as an innovative technology for assessing the efficacy and toxicity in bladder cancer treatment [804]. Moreover, clinical studies have evaluated the safety, pharmacokinetics, and pharmacodynamics of resveratrol in subjects with colorectal cancer and multiple myeloma, as well as its potential for cancer prevention in healthy participants (NCT00920803, NCT00920556, and NCT00098969). This section summarizes the complex mechanisms and specific targets of resveratrol against various types of cancer in the following sections.

### Resveratrol enhances tumor immunity

Resveratrol has a critical role in modulating the immune system and enhancing its ability to recognize and attack cancer cells. It affects various immune cells, including B cells, TAMs, MDSC, T cells and NK cells, and regulates the tumor immune microenvironment and exerts anti-tumor effects in lung, breast, and oral cancers by increasing the number and activity of immune cells [805, 806]. Resveratrol inhibits cancer proliferation by reversing the 2,3,7,8-Tetrachlorodibenzo-p-dioxin (TCDD)-induced increase in the number and the immunosuppressive function of MDSCs in mice [807]. Also, resveratrol acts on TAMs, promoting the M1/M2 macrophage polarization ratio, inhibiting the conversion of TAMs from M1 to M2 subtype, and exerting an inhibitory effect in the microenvironments of tumor cells in lung, breast, and oral cancers [808–810]. Resveratrol also affects T cell function by inhibiting tumor growth through activation of effector T cells [811], as well as influencing T cell function by increasing the proliferation of cytotoxic CD8^+^ T cells and enhancing their ability to produce cytokines, such as IFN-*γ*, to kill tumor cells [812–814]. NK cells are also crucial for targeting and destroying tumor cells and are suitable targets for cancer immunotherapy. Resveratrol enhances the cytotoxic activity of NK cells, upregulates the expression of NK cell activation receptors to improve their ability to recognize and kill cancer cells [815, 816]. Resveratrol can modulate the tumor immune microenvironment by altering cytokine production. It reduces the levels of pro-inflammatory cytokines (e.g., IL-6 and TNF-*α*) [817] and pro-carcinogenic cytokines (TGF-*β*1, TNF-*β*, and CXCL12) [818] which exerts an inhibitory effect on pancreatic cancer associated fibroblasts (CAF) and colon cancer (HCT 116) cells [819, 820]. Resveratrol exerts immunomodulatory effects by acting on multiple immune checkpoints (PD-L1, PD-1, CTLA-4, NKG2A, TIM-3, and BTLA). It includes altering the tumor immune microenvironment to inhibit the value-added of ovarian and oral cancer cells [821, 822], as well as weakening the inhibitory function of Tregs on effector T-cells [823], to promote the killing of cancer cells. Therefore, resveratrol plays a multifaceted role in enhancing anti-cancer immunity by modulating various immune cells, cytokine production, and immune checkpoints. By promoting the activity of cytotoxic T cells, NK cells, and macrophages, while simultaneously inhibiting the immunosuppressive functions of MDSCs and Tregs, resveratrol creates a more favorable immune microenvironment for anti-tumor immunity. Additionally, its ability to regulate cytokines and immune checkpoints further strengthens its potential as an immune-modulatory agent in cancer treatment.

### Resveratrol reverses tumor drug resistance

Resveratrol has been shown to inhibit drug transporters, thereby increasing the intracellular concentration of chemotherapeutic drugs. Also, resveratrol can inhibit prostate cancer and of oral squamous cell carcinoma drug resistance by down-regulating EGFR. This property allows it to participate in the therapeutic process of various anti-cancer drugs (paclitaxel, celastrol, adriamycin, rapamycin, and cetuximab) [824–827]. Specifically, resveratrol reduces the protein expression levels of P-gp, MRP1, BCRP, CYP3A4, GST and mRNA expression of hPXR to restore the sensitivity of NSCLC and colon cancer cells to polyphenols. The combination of quercetin-resveratrol can reduce the levels of EGFR, EGR3 and IL6, and increase the levels of IGFBP7 and NKX3.1, which can play an anti-prostate cancer role [825]. Resveratrol administration also increases the sensitivity of oral squamous cell carcinoma to cetuximab by decreasing the protein expression of uPAR, which inhibits the signaling molecule ERK1/2 downstream of EGFR [828]. Thus, resveratrol plays a crucial role in reversing tumor drug resistance by targeting drug transporters, down-regulating EGFR expression, and enhancing the efficacy of chemotherapeutic agents. By inhibiting drug efflux pumps and modulating key signaling pathways, resveratrol restores the sensitivity of cancer cells to chemotherapy and targeted therapies, improving treatment outcomes.

### Resveratrol inhibits cancer progression by regulating the process of autophagy and ferroptosis

Resveratrol has been shown to induce autophagy and ferroptosis in lung, breast, pancreatic, ovarian cancers, as well as myeloma, leading to cell death and tumor growth inhibition [829, 830].It activates key autophagy-associated proteins, such as Beclin-1 and LC3, which promote the degradation of damaged organelles and proteins [831–833], as well as increasing the phosphorylation of AMPK and decreasing the phosphorylation of mTOR and its downstream substrates in a dose-dependent manner, leading to autophagy [832]. Resveratrol targets neuroblastoma by inducing intrinsic apoptosis, which is mediated by the intracellular ROS axis and ER stress [834]. In the context of autophagy as a survival mechanism, resveratrol can sensitize cancer cells to chemotherapy by inducing lysosomal membrane permeabilization (LMP) which attenuates the fusion of autophagosomes and lysosomes [835]. Resveratrol induces iron death in carcinoma cells by regulating the expression of iron death-related genes [836, 837]. In the lung squamous cell carcinoma (LUSC) tumor immune microenvironment, resveratrol regulates SLC7A11-HMMR interaction, activates iron death and potentiates the cytotoxicity of CD8^+^ T cells to exert antitumor effects. In addition, RSV can induce iron death in colon cancer cells by promoting lipid peroxidation and inhibiting the protein expression of SLC7A11 and GPX4 [838]. Thus, resveratrol’s ability to regulate both autophagy and ferroptosis plays a critical role in its anti-cancer effects. By inducing autophagy, resveratrol promotes the degradation of damaged cellular components, leading to cell death and inhibition of tumor growth. Moreover, its ability to induce ferroptosis through lipid peroxidation and the regulation of ferroptosis-related genes makes resveratrol a potent therapeutic agent in cancers that are susceptible to this form of cell death.

### Resveratrol inhibit cancer in classical modalities

Resveratrol exerts its anti-cancer effects in colon, breast, and liver cancers by disrupting cell cycle and preventing proliferation of cancer cells [839–844]. For instance in colon cancer and lymphoma, it induces cell cycle arrest at G0/G1 phase [813, 845], and in cervical cancer and melanocytoma, it stops in G1 phase [846, 847]. This effect is mediated by the regulation of cyclin and CDK, which are important regulators of cell cycle progression. Resveratrol increases the protein expression of CDK inhibitors (p15), which block cell cycle progression and induce growth arrest [848]. Resveratrol's ability to regulate the cell cycle involves several molecular targets: In cells with functional p53, resveratrol enhances p53 activity, leading to cell cycle arrest and apoptosis [849]. Resveratrol also affects the checkpoint kinase Chk2, which is involved in the DNA damage response and cell cycle regulation and plays a crucial role in maintaining the stability of the tumor microenvironment [850]. Resveratrol activates Caspase-3 and Caspase-8, and up-regulates the protein expression of Bax [851–853], while inhibiting anti-apoptotic proteins like Bcl-2 [854], in order to induce apoptosis in luminal squamous cell carcinoma, cervical carcinoma, colon carcinoma, ovarian carcinoma, and uterine leiomyoma cells. Specifically, it activates Caspase-3 to exert anti-tumor effects in luminal squamous cell carcinoma and ovarian cancer [855, 856]. In colon cancer and uterine leiomyoma, it promotes apoptosis through Bcl-2 protein expression and increases Bax protein expression [853, 857]. Resveratrol can activate Caspase-3 in breast cancer (MCF-7) cells, leading to the breakdown of cellular substrates and successful initiation of apoptosis [858]. Moreover, resveratrol has been shown to inhibit the protein expression of fibronectin, N-calmodulin, waveform protein and increase the protein expression level of E-calmodulin in breast and colon cancer cells to inhibit tumor cell invasion or migration [798, 834, 859]. Therefore, resveratrol exerts its anti-cancer effects through the regulation of the cell cycle, induction of apoptosis, and inhibition of tumor cell migration and invasion. By arresting the cell cycle at various stages and enhancing the expression of pro-apoptotic proteins while inhibiting anti-apoptotic ones, resveratrol effectively suppresses cancer cell proliferation and induces cell death.

### Resveratrol exhibits synergistic effects in cancer treatment

Resveratrol can significantly boost the anti-cancer impact when combined with a range of chemotherapeutic drugs. Studies have shown that resveratrol and curcumin have emerged as effective chemopreventive and chemoprotective compounds [860]. Their combination can effectively reduce bladder cancer (T24-GCB), colon cancer cell survival induced stronger ER stress and upregulation of the pro-death UPR molecule CHOP and exerted stronger cytotoxic effects [795, 800, 861]. Besides, resveratrol in combination with 5-fluorouracil induced S-phase cell cycle arrest and apoptosis of CD133^+^ colon cancer stem cells by regulating *Bax* gene [862]. Resveratrol also sensitizes colon cancer (Caco-2) and lung cancer (NSCLC) cells to paclitaxel by inducing apoptosis. This combination leads to increased cytotoxicity by increasing ROS levels, inducing oxidative DNA damage and decreasing cellular antioxidant defenses, resulting in effective apoptosis induction in cancer cell lines [863]. In various cancer cell lines and in vivo tumor models, resveratrol enhances the antitumor efficacy of rapamycin (An inhibitor of the autophagy inducer of mTORC1) by reducing the autophagy induced by AKT phosphorylation in thyroid cancer (KTC-1 and TPC-1) cells [801]. Thus, resveratrol’s ability to enhance the effects of various chemotherapeutic drugs provides a compelling rationale for its use in combination therapies. By inducing oxidative stress, modulating apoptosis pathways, and regulating the cell cycle and autophagy, resveratrol synergistically enhances the efficacy of drugs like curcumin, 5-fluorouracil, paclitaxel, and rapamycin.

### Resveratrol derivatives in cancer treatment

Derivatives of resveratrol exhibit strong anti-cancer potential, and some of them have been used in colon and breast cancer therapy. Astragalus is a dimethylated derivative of resveratrol, which inhibits Top1 enzyme activity and treats colon cancer through the Top1/Tdp1-mediated DNA repair pathway with low toxicity [864]. Moreover, astragalus achieves its inhibitory effect on the tumor ball formation and migratory capacity of HeLa cancer stem-like cells by inhibiting the protein expression levels of stemness markers (e.g., CD133, Oct4, Sox2, and Nanog), as well as STAT3 signaling [865]. Besides, resveratrol derivatives inhibits human topoisomerase II and induces apoptotic cell death, and shows antitumor activity in different types of breast cancer (ER-positive MCF-7 and ER-negative SKBR3) cells [866]. Overall, these classes of derivatives have been designed to be more readily absorbed and retained in the body, thereby increasing their effectiveness in reaching and acting on target tissues, as well as the need for smaller doses sufficient to achieve the desired therapeutic effect [867, 868]. Thus, resveratrol derivatives, including astragalus, demonstrate strong anti-cancer potential by targeting critical molecular pathways involved in DNA repair, cancer stem cell maintenance, and apoptosis. These derivatives exhibit enhanced bioavailability and tissue targeting, making them more effective in treating cancers such as colon and breast cancer. By improving pharmacokinetics and reducing toxicity, resveratrol derivatives offer a promising avenue for developing more efficient and less toxic cancer therapies.

### Application of multi-omics techniques to investigate the anti-cancer properties of resveratrol

Thirty-eight target proteins are identified by proteomics analysis, which shows that the targets are mainly involved in cytoskeletal remodeling and EMT. It shows that resveratrol can inhibit A549 cell migration by binding to multiple targets to regulate cytoskeletal remodeling and inhibit EMT in order to exert anti-cancer effects [869].

### Investigating resveratrol anti-cancer effects through drug delivery system

Pharmacokinetic studies have demonstrated that despite being absorbed after oral administration, resveratrol undergoes rapid and extensive Phase I and Phase II metabolism in rodents and humans. Therefore, researchers have designed novel DDS (nanoparticles, micelles, gels, and liposomes) to improve the bioavailability and therapeutic efficacy of resveratrol in cancer. Nanoparticle-based resveratrol delivery systems include resveratrol-loaded nanocrystals (NANO-RSV), silica mesoporous nanoparticle (MSN)-based DDS, and biocompatible resveratrol-conjugated gold nanoparticles (Res-AuNPs) [870–872]. Their advantages are commonly demonstrated by the prolonged release of resveratrol at the tumor site and significant in vitro therapeutic effects [871, 873]. Besides, hydrogels co-doped with dopamine-reduced graphene oxide (DOPA-rGO; a photothermal nano-agent) and resveratrol exhibit injectability and in situ gelation, along with suitable physicochemical properties and good cytocompatibility. Chemo-photothermal therapy (RES + DOPA-rGO@Gel + NIR light) of the hydrogel was able to reduce the viability of breast cancer cells to only 31% [874]. In addition, resveratrol-loaded cationic liposomes (RLs) achieved specific delivery in liver cancer cells, as evidenced by a significant reduction in liver marker enzymes [875]. Therefore, resveratrol-loaded nanocrystals, silica mesoporous nanoparticles, gold nanoparticles, hydrogels, and cationic liposomes represent promising DDS platforms that can be further optimized for clinical use. These systems not only overcome the pharmacokinetic limitations of resveratrol but also enable targeted and controlled drug release at tumor sites, enhancing the drug's anti-cancer effects.

Overall, resveratrol exerts multi-faceted anti-cancer effects by modulating tumor immunity (e.g., boosting cytotoxic T cells and NK cells), reversing drug resistance (via down-regulating EGFR and P-gp), inducing autophagy and ferroptosis, and arresting cell cycle progression while promoting apoptosis. It also enhances the efficacy of standard chemotherapies (e.g., 5-fluorouracil and paclitaxel) through synergistic mechanisms involving oxidative stress and apoptosis pathways. Furthermore, resveratrol derivatives (e.g., astragalus) show improved bioavailability and strengthen anti-cancer outcomes by inhibiting DNA repair or stemness markers in tumor cells. Leveraging multi-omics findings and advanced DDS (nanoparticles, hydrogels, and liposomes) further increases its therapeutic impact and addresses pharmacokinetic limitations.

### Silibinin

Silibinin, a flavonolignan derived from the seeds and fruits of Silybi fructus or *Silybum marianum* (Shuifeiji in Chinese), exhibits a diverse array of pharmacological effects, including hepatoprotective, anti-inflammatory, antioxidative, anti-fibrotic, and anti-tumorigenic properties [876–878]. Research indicates that silibinin effectively inhibits tumor growth across a broad spectrum of cancers such as breast, ovarian, lung, colon, pancreatic, liver, renal cell, endometrial, and bladder cancers [879]. Silibinin has multi-anticancer effects in vitro and in vivo, it inhibits cancer progression through regulating tumor immunity, overcoming drug resistance, boosting autophagy and ferroptosis, triggering apoptosis, inducing cell cycle arrest, and so on. It achieves these effects through targeting various cellular molecules—including TNF-*α*, IL-6, and VEGF—and impacting several signaling pathways such as the Wnt/*β*-catenin and PI3K/AKT/mTOR pathways [880–882]. Moreover, silibinin shows potential as an adjuvant in mitigating numerous side effects of anti-cancer therapies, such as hepatotoxicity and neurotoxicity [883, 884]. In silico analysis reveals ENO1, GLUT4 and ID3 as potential targets for disrupting the metabolic network in breast cancer cells, with silibinin promoting cytotoxicity, apoptosis, and lipid metabolism reprogramming [885, 886]. In clinical studies, silibinin shows promising anti-cancer activity with minimal toxicity in conditions including colon and head and neck cancers [887, 888]. Recent clinical research focus on its efficacy in treating brain metastases from cancers like leptomeningeal carcinomatosis, lung cancer, and breast cancer (NCT05793489, NCT05689619). The following section is a summary of the relevant pharmacological mechanisms of the anti-cancer effect of silibinin.

### Silibinin improves tumor immunity

Silibinin inhibits tumor growth through immunomodulatory activities, such as regulating immune cells (Tregs, macrophages, DCs, NK cell), inhibiting inflammatory cytokines (IFN-*γ*, IL-2, IL-6, IL-10, IL-12, TNF-*α*, and TGF-*β*), and modulating immune check points (PD-L1 and PD-1) [889–893]. Silibinin can regulate the proliferation, differentiation, and function of immune cells. Silibinin improves cancer immunity by promoting T cell proliferation, increasing the quantity of T cells, and enhancing T cell responses and function [894, 895]. Silibinin effectively remodels the tumor microenvironment by enhancing the activation of NK cells, which boosts their ability to recognize and eradicate breast cancer (4T1, MCF-7, and MDA-MB-231) cells [890]. Additionally, it facilitates the polarization of macrophages towards the M1 phenotype, augmenting their phagocytic and antigen-presentation capabilities, thereby strengthening the body's antitumor immune response [891, 896]. Silibinin also regulates cytokine secretion, increasing IL-12 and IFN-*γ* levels while reducing TGF-*β*, SDF-1, IL-6, and TNF-*α*, all of which play crucial roles in immune regulation and anti-cancer immunity in 4T1 breast cancer bearing mice [890]. Moreover, silibinin mitigates immunosuppression and inhibits tumor progression by limiting PD-L1 protein expression in lung cancer (A549, H292, and H460), papillary thyroid cancer (TPC-1), and nasopharyngeal carcinoma (C666-1) cells [889, 892, 897]. It also induces immunogenic cell death (ICD) in colon cancer (CT26) and melanoma (B16-F10) cells by elevating the protein expression of CRT, HSP70, and HMGB1, which are important in anti-tumor responses [895]. Furthermore, silibinin may modulate immunity by suppressing HIF-1*α* in colon cancer (CT26) and ovarian clear-cell carcinoma (HAC-2, OVISE, and RMG-1) cells, influencing the tumor immune landscape [878, 898]. Thus, silibinin demonstrates significant potential as an immune-modulatory agent in cancer therapy. By enhancing T cell proliferation, NK cell activation, and macrophage polarization, silibinin strengthens the body’s immune response against tumors. Additionally, its ability to regulate cytokine secretion, inhibit immune checkpoints, and induce immunogenic cell death further supports its potential in cancer treatment. Silibinin’s effects on HIF-1*α* also suggest that it can modulate the tumor microenvironment, improving immune cell function and tumor recognition.

### Silibinin reverses tumor drug resistance

Emerging evidence suggests that silibinin can reverse tumor drug resistance through blocking P-gp and MRP1 and inhibiting transcriptional activation in prostate, ovarian, lung, and liver cancers [899]. Silibinin augments the efficacy of cisplatin and paclitaxel by diminishing cell adhesion to the extracellular matrix in ovarian cancer (A2780) and colon cancer (CT26) cells, thereby increasing drug sensitivity [900, 901]. Furthermore, silibinin modulates lung cancer (H3122 and H2228) cells by enhancing the therapeutic effects of nintedanib, brigatinib, and lorlatinib through the inhibition of STAT3 activity [902, 903]. Additionally, silibinin improves radiosensitivity in prostate cancer (DU 145) cells by mitigating DNA damage, particularly following EGFR knockdown [904]. Therefore, silibinin offers significant promise as a therapeutic agent in reversing tumor drug resistance through various mechanisms, including inhibition of drug efflux pumps, modulation of cell adhesion, and regulation of key signaling pathways like STAT3. Its ability to enhance the efficacy of both conventional chemotherapy and targeted therapies, as well as improve radiosensitivity.

### Silibinin inhibits cancer progression by regulating the process of autophagy and ferroptosis

Silibinin has been shown to induce autophagy in several cancer types by modulating various molecular pathways. Specifically, silibinin decreases phosphorylated mTOR protein levels, which in turn induces autophagy and inhibits the progression of liver cancer, breast cancer, and glioma [905–907]. Notably, silibinin also influences the autophagic degradation of YAP in castration-resistant prostate cancer (PC-3 and C4-2) and breast cancer (MCF-7 and MDA-MB-231) cells, highlighting its potential in autophagy modulation [882, 908]. Additionally, it down-regulates the Wnt/*β*-catenin signaling pathway, a key regulator of autophagy, and prevents EMT in renal cell carcinoma (786-O and ACHN) and liver cancer (Hep G2) cells [909, 910]. In breast cancer (MCF-7) cells, silibinin triggers nuclear translocation of apoptosis-inducing factor (AIF), mediated by the inhibition of the ER*α* pathway and enhanced autophagy [911]. Moreover, silibinin's interaction with PINK1 and Parkin promotes mitophagy, reduces ROS levels, and enhances cellular resilience in liver cancer (Hep G2) and breast cancer (MCF-7 and MDA-MB-231) cells [912, 913]. Silibinin also impacts the process of ferroptosis by modulating various signaling pathways and molecular mechanisms, including NF-*κ*B and STING pathways [914, 915]. It regulates intracellular oxidative stress and iron metabolism, influencing the occurrence of ferroptosis by decreasing the protein expression of ACSL4 [915]. Thus, by modulating the mTOR pathway, inducing autophagic degradation of YAP, and inhibiting the Wnt/*β*-catenin signaling pathway, silibinin suppresses tumor growth and metastasis in various cancers. Furthermore, silibinin's ability to regulate mitophagy and ferroptosis enhances cellular resilience and modulates oxidative stress, further contributing to its anti-cancer effects.

### Silibinin inhibits cancer progression via classical modalities

Silibinin exerts cell cycle arrest across various phases including G0/G1, S, or G2/M, across multiple cancer types such as endometrial carcinoma, cervical, and lung cancer [916]. Substantial evidence confirms silibinin's predominant action in inducing G2/M phase arrest in a range of cancers including endometrial, cervical, bladder, prostate, renal, and colon cancers [917]. Specifically, in human epithelial colon adenocarcinoma (Caco-2) cells, silibinin decreases S phase populations and inhibits mitosis (G2/M) [918]. Furthermore, it suppresses cell proliferation and enforces G0/G1 phase arrest by targeting Cyclin D, cyclin E, and CDK4 in cholangiocarcinoma (HuCCT-1 and CCLP-1) and NSCLC (A549, H292, and H460) cells [889, 919]. Numerous researches have reported that silibinin can promote the apoptosis by regulating apoptotic-related proteins (e.g., Bax, Bcl-2, and Bcl-xL) and activating Caspases in lung, colon, renal, oral, pancreatic cancers, as well as endometrial carcinoma [920, 921]. It effectuates tumor cell apoptosis through the activation of the JNK/c-Jun and JNK/SAPK pathways, leading to reduced Bcl-2 levels and elevated Bax, as well as activated Caspase-3 and PARP in oral cancer (YD10B and Ca9-22) and pancreatic cancer (SW1990) cells [922, 923]. Additionally, silibinin induces apoptosis in oral cancer (YD10B, Ca9-22, and SCC-25) cells and renal tissues from carcinogenesis models by releasing Cytochrome c [922, 924, 925], and triggers mitochondrial damage in human epidermal cancer (A-431) cells [926]. Silibinin also modulates mitochondrial dynamics by up-regulating DRP1, and down-regulating OPA1, MFN1, ATP content, and mitochondrial biogenesis regulatory factors (TFAM, PGC1*α*, and NRF2), which promotes both extrinsic and intrinsic pathways of apoptosis in breast cancer (MCF-7 and MDA-MB-231) cells [927].

Silibinin's capacity to inhibit tumor progression is further demonstrated through its anti-angiogenic and anti-metastatic effects in colon, rectal, lung, prostate, bladder, breast, oral, and papillary thyroid cancers [928]. It obstructs the nuclear translocation of YAP/TAZ, leading to apoptosis in breast cancer (MCF-7 and MDA-MB-231) cells [908], and mitigates migration and invasion in breast cancer (MDA-MB-231) cells by reducing oxidative stress, suppressing NLRP3 inflammasome activation, enhancing mitochondrial fusion, and down-regulating *RAC1* mRNA [927, 929]. Moreover, silibinin curtails migration and invasion in oral and prostate cancer cells by inhibiting EMT and modulating the protein level of N-cadherin, vimentin, and E-cadherin [882, 922]. It also reduces proliferation and migration in lung cancer (A547) cells by targeting RHBDD1 [930]. Therefore, silibinin exerts its anti-cancer effects through multiple classical mechanisms, including cell cycle arrest, apoptosis induction, and the inhibition of angiogenesis and metastasis. By targeting key molecular pathways such as cyclin/CDK complexes, JNK/c-Jun signaling, and mitochondrial dynamics, silibinin effectively suppresses tumor growth and progression. Furthermore, its ability to modulate tumor cell migration and invasion, particularly through the regulation of EMT and mitochondrial fusion.

### Silibinin combined with other medications for cancer treatment

The combination of silibinin with first-line chemotherapy drugs has shown potential to enhance the efficacy of cancer treatments while mitigating drug-related side effects [931, 932]. Silibinin combined with neratinib or etoposide significantly promotes apoptosis in breast cancer (4T1, MCF-7, and MDA-MB-231) cells by up-regulating the protein expression of p53 and p21 [932, 933]. Furthermore, the combination of silibinin with paclitaxel or metformin enhances anti-cancer effects in lung cancer (A549) in vitro and in vivo by increasing chemotherapy sensitivity, modulating the tumor microenvironment, and effectively inducing apoptosis [931, 934, 935]. Moreover, co-treatment with silibinin and IPI-549 improves anti-cancer efficacy in breast cancer by inhibiting cell migration in vitro and in vivo, while significantly reducing Tregs and MDSCs [936]. Additionally, combining silibinin with other natural compounds further inhibits tumor cell migration. For example, silibinin combined with curcumin significantly downregulates Ob-R protein expression in breast cancer (MCF-7) cells [937]. Likewise, silibinin and epigallocatechin-3-gallate synergistically suppress lung cancer (A549) cell migration by down-regulating pro-angiogenic factors associated with the *miR-17–92* cluster and up-regulating anti-angiogenic factors linked to *miR-19b* [938].

Silibinin also demonstrates protective effects against chemotherapy-induced toxicity. It reduces hepatotoxicity and neurotoxicity caused by drugs such as cisplatin and paclitaxel by mitigating DNA damage and restoring cellular proliferative potential in ovarian cancer (A2780/DDP) cells and cisplatin-treated mice [883, 900]. In addition, silibinin-loaded PLGA nanoparticles alleviate dacarbazine (DTIC)-induced hepatotoxicity in a melanoma mouse model by lowering liver enzyme and bilirubin levels and upregulating detoxification enzymes like NQO1 and GSTP1 [884]. Clinical studies further underscore the potential of silibinin in reducing side effects associated with cancer therapies, improving chemotherapy and radiotherapy responses, and enhancing overall clinical outcomes [902, 939, 940].

### Silibinin derivatives inhibit cancer progression

Based on the promising anti-cancer properties of silibinin, various silibinin derivatives have been extensively studied in recent years, including oxidized derivatives such as 2,3-dehydrosilybin, carbamate-modified silibinin, 2,3-dehydrosilybin derivatives with carbamate groups, and glycosylated silibinin derivatives. These derivatives improved solubility and enhanced biological activity compared to silibinin, demonstrating stronger anti-cancer and sensitization effects. Mechanistically, they enhance anti-proliferative and anti-inflammatory activities, induce cell cycle arrest (G2/M phase), promote cell apoptosis, and modulate the activity of proteins such as P-gp and acetylcholinesterase [941–943]. Notably, several 7-O-tyrosyl silibinin derivatives have shown significant anti-prostate cancer efficacy by inducing pronounced G0/G1 phase arrest and apoptosis [944]. Compared to silibinin, these derivatives provide superior solubility, enhanced biological activity, and exert their anti-cancer effects through diverse mechanisms, including targeted cell cycle arrest and induction of apoptosis. This highlights the potential of silibinin derivatives as improved therapeutic agents in cancer treatment.

### Investigating silibinin anti-cancer effects through drug delivery system

The low water solubility and poor bioavailability of silibinin limit its therapeutic efficacy at tumor sites. To address these challenges, various nano-based delivery systems, including nanoparticles, cationic liposomes, smart nano-caged carriers, and other nano-formulations, have been developed to replace traditional formulations and effectively inhibit the progression of cancers such as breast, ovarian, and prostate cancers [945].

Silibinin encapsulated in nano-carriers can enhance the effects of drugs on apoptosis and immunogenic cell death in melanoma (B16-F10) cells [946]. For instance, PEG 400-OA as a nano-carrier significantly enhances the bioavailability and stability of silibinin [947]. Silibinin-gold nanoparticles (Sb-GNPs) prepared through physical adsorption and inhalable silymarin-loaded solid lipid nanoparticles exhibit narrow size distributions, increased bioavailability, and enhanced cytotoxic effects, increasing the cell-killing efficacy by 4–5 times in human lung adenocarcinoma (A549) cells [948, 949]. Chitosan nanoparticles loaded with silibinin significantly upregulate the gene expression of *Bax* and *Caspase-3* in glioma (C6) cells, effectively inducing apoptosis [950]. Similarly, silibinin-loaded magnetic niosome nanoparticles minimize systemic drug distribution side effects while maximizing therapeutic effects by improving targeting and cytotoxicity in colon cancer (HT-29) cells [951]. Therefore, nano-based formulations of silibinin demonstrate significant improvements in drug bioavailability, stability, and apoptotic efficacy across various cancer models, highlighting their potential as cutting-edge therapeutic alternatives.

### Silibinin intervenes in cancer through epigenetic modifications

Silibinin exerts its anti-cancer effects via epigenetic mechanisms, notably through the modulation of miRNA expression and the consequent dysregulation of their target genes, alongside alterations in DNA methylation patterns [947, 952, 953]. MicroRNAs (miRNAs) are a highly conserved class of RNAs, whose improper regulation has been associated with human diseases, including cancer. Research indicates that silibinin can reduce the levels of miRNAs (*miR-125b*, *miR-182*, and *miR-20b*), and up-regulate the levels of pro-apoptotic genes (*p53*, *Bax*, *Caspase-9*, *PTEN*, and *Bcl2L11*), decrease the level of anti-apoptotic gene (*Bcl-2*), thereby inducing apoptosis in breast cancer cells (T-47D, MCF-7, and MDA-MB-231) [947, 954]. In pancreatic cancer cells (MIA PaCa-2 and PANC-1), slibinin induces apoptosis and suppresses migration by downregulating certain onco-miRNAs (*miR-155*, *miR-222*, and *miR-21*) and their associated targets (*Bcl-2*, *CD34*, *AKT3*, *MASPINE*, *EGF*, and *BMP7*). Additionally, it promotes the expression of tumor-suppressor miRNAs (*miR-34a*, *miR-126*, and *miR-let7b*) and enhances the gene levels of *Caspase-9*, *p53*, *APAF1*, and *Bax* [952, 955]. Moreover, silibinin induces programmed cell death in human hepatocarcinoma (Hep G2) cells by promoting the secretion of specific miRNAs (*miR223-3p* and *miR16-5p*) that potentially target the PTEN/AKT signaling pathway, and enhances the cytotoxic effect of sorafenib through the downregulation of *miR-92a* [956]. Thus, silibinin’s ability to modulate epigenetic mechanisms, particularly through the regulation of miRNA expression and DNA methylation, enhances its anti-cancer potential. By down-regulating oncogenic miRNAs and up-regulating tumor suppressor miRNAs, silibinin induces apoptosis and inhibits cell migration in various cancers, including breast, pancreatic, and liver cancers. Furthermore, its ability to modulate the PTEN/AKT pathway and enhance the efficacy of chemotherapy drugs such as sorafenib underscores its potential in combination therapies.

### Application of multi-omics techniques to investigate the anti-cancer properties of silibinin

Recent advancements in omics technologies, including genomics, transcriptomics, proteomics, and metabolomics, have significantly enhanced the capability to elucidate the anti-cancer mechanisms of silibinin [957, 958]. For instance, transcriptomic analysis have provided a more precise understanding of silibinin's influence on gene expression within various lung cancer (H1650, H1975, A549, H838, and H2030) cells, identifying a connection between silibinin and the modulation of key STAT3 target genes (*BIRC5*, *BRCA1*, and *FOXM1*) that contribute to its anti-cancer effects [958]. Proteomics identified proteins that were highly expressed when silibinin intervened in basal cell carcinoma, and further analyzed their pathways, discovering a new target of RAC2 [959]. Metabolomics analysis based on nuclear magnetic resonance (NMR) and mass spectrometry showed that silibinin restrains glucose metabolism to impede the biosynthetic requirements of breast cancer cells and increases levels of BCAAs isoleucine, leucine, and valine, thereby inducing apoptosis in breast cancer (MDA-MB-468, MDA-MB-231, and BT-549) and prostate cancer (PC-3) cells [960, 961]. Therefore, the application of multi-omics techniques has provided a deeper understanding of silibinin’s anti-cancer properties, revealing its complex interactions with cancer cell molecular, protein, and metabolic networks. By modulating key signaling pathways like STAT3, targeting novel proteins such as RAC2, and interfering with cancer cell metabolism, silibinin demonstrates its potential as a multifaceted therapeutic agent.

In sum, silibinin stands out for its ability to orchestrate multiple anti-cancer pathways, spanning immunity, autophagy, and epigenetic control, while also optimizing standard therapies and alleviating their toxicities. By reshaping key signaling axes (e.g., PI3K/AKT and Wnt/β-catenin) and rewiring metabolic and microRNA networks, it imposes broad restraint on tumor progression. These insights from multi-omics profiling underscore silibinin’s translational promise across various malignancies.

### Triptolide

Triptolide, a diterpenoid triepoxide, is extracted from Tripterygium wilfordii (Leigongteng in Chinese) [962]. Triptolide demonstrates a wide range of activities, such as anti-inflammatory, immunosuppressive, neuroprotective, prevention of bone loss and anti-tumor effects [963, 964]. Triptolide has been shown to possess a broad spectrum of anti-cancer characteristics and to be efficacious against several cancer types, including lung, breast, colon, bladder, liver, gastric, head and neck, thyroid, pancreatic cancers, as well as glioblastoma, melanoma, and leukemia [965]. In silico analysis identifies key targets such as CDKN1A, c-JUN, RELA, and TP53, suggesting that triptolide may treat thyroid cancer by inhibiting cell proliferation, inducing apoptosis, and modulating inflammatory pathways like NF-*κ*B and MAPK [966]. Furthermore, ex vivo study demonstrates that triptolide effectively inhibits the growth of colorectal cancer organoids, indicating its potential to disrupt cancer cell proliferation and tumor formation at the organoid level [964]. Additionally, triptolide impedes muscle development and cell proliferation in zebrafish larvae by interfering with several signaling pathways, including Notch1 and STAT3, which are also involved in cancer cell regulation [967]. Meanwhile, the prodrug of triptolide, has been developed in clinical trials for patients with advanced solid tumors (NCT03129139) and refractory pancreatic malignancy (NCT03117920 and NCT04896073). The subsequent section presents a succinct synthesis elucidating the intricate modalities and precise molecular targets by which triptolide manifests its therapeutic actions across a spectrum of malignancies.

### Triptolide improves tumor immunity

Triptolide exerts notable immunomodulatory effects by engaging with various immune cell populations, such as macrophages, NK cells and T cells, across different cancer types including glioma, oral, breast, and colon cancers [963, 968]. Recent study demonstrated that triptolide directly affects the tumor microenvironment by selectively depleting TAMs, inhibiting the function of M2 macrophage differentiation and viability, and suppressing M2 marker expression and anti-inflammatory cytokine secretion, leading to reduced tumor burden in experimental models of cancer [969, 970]. Specifically, triptolide reduces TAM infiltration in colon cancer (HT-29 and CT26) cells by down-regulating CXCL12 gene and protein expression via the NF-*κ*B-ERK1/2 signaling axis, while delaying M2 polarization. In combination with aspirin, triptolide further blocks NF-*κ*B activation in cancer cells through distinct mechanisms involving p53 and ERK1/2, contributing to a reduction in cancer incidence [971, 972]. Furthermore, triptolide suppresses M2 polarization of TAMs in vitro functioning as an integrin *β*3 inhibitor [973]. For T cells, triptolide is reported to overturn T cell suppression, particularly CD4^+^ T cells subset in glioma (T98G) cells under IFN-*γ* treatment, while significantly decreases the proportion of Tregs in the spleen and axillary lymph nodes of melanoma (B16-F10) bearing mice, thereby impairing their suppressive function through the down-regulation of Foxp3, IL-10, and transforming growth factor in both protein and mRNA levels [974, 975]. While triptolide presents challenges due to its water-insolubility and high cytotoxicity, innovative delivery systems and derivatives have enabled diverse anti-cancer mechanisms, suggesting promising avenues for future research and therapeutic development.

Dextran-conjugated triptolide and triptolide nano-MOFs enhance TNF-*α* levels, promoting an immune response that ultimately inhibits tumor progression in pancreatic cancer with KRAS mutation, while also reducing toxicity compared to free triptolide [976, 977]. Triptolide attenuates IL-6-induced activation of STAT3 target genes, such as *Mcl-1* and *Bcl-2*, by decreasing STAT3 phosphorylation and inhibiting its nuclear translocation in NSCLC (PC9 and A549) cells, while also reversing *IL-4* or LPS-induced up-regulation of *IL-6* at the gene level, thereby reducing cell survival, apoptosis, and the polarization of myeloid cells into immunosuppressive macrophages and MDSCs within the tumor microenvironment [978, 979]. In a colitis-induced colon cancer model, triptolide further suppresses IL-6 secretion and reduces the expression of IL-6 receptor (IL-6R), JAK1, and phosphorylated STAT3 proteins. By inhibiting the IL-6R-JAK/STAT signaling pathway, triptolide impedes tumor cell proliferation, survival, and the inflammatory response, which are critical for cancer progression [980]. Moreover, triptolide inhibits *CXCL12* gene expression within the tumor microenvironment, leading to the suppression of the *CXCL12*/*CXCR4* axis and a reduction in TAM infiltration. This is achieved through downregulation of tumor-derived *CXCL12 *via the NF-*κ*B/ERK1/2 signaling pathway, which delays macrophage polarization towards the M2 phenotype. As a result, there is a decrease in the gene expression of immunosuppressive markers such as *Arg-1*, *CD206*, and *IL-10* in both human and mouse colon tumors [971, 981]. Therefore, triptolide demonstrates multifaceted anti-tumor effects across various cancer models by inhibiting cytokine production, tumor progression, and angiogenesis, while also modulating the tumor microenvironment to enhance anti-cancer immunity and minimize toxicity.

Triptolide treatment significantly suppresses PD-L1 expression in oral squamous cell carcinoma and NSCLC, modulating the immune checkpoint response and effectively inhibiting tumor growth in PDTX models and an IFN-*γ*-modulated environment, while its combination with IFN-*γ* in locoregional treatment enhances antitumor immunity in triple negative breast cancer by reversing IFN-*γ*-induced PD-L1 expression and activating cytotoxic CD8^+^ T lymphocytes, resulting in synergistic tumor growth inhibition [982–984]. Interestingly, in glioma (U251-MG, T98G, U-87 MG, A172, LN-229, and LN-18) cells, triptolide downregulates PD-L1 protein expression induced by IFN-*γ* on the cell surface, helping to reverse T cell inhibition, particularly affecting CD4^+^ T cells, and counteracting immunosuppression within the tumor microenvironment [974]. Moreover, treated with triptolide resulted in a significant reduction in the protein expression of CD47 and CD126 in the THP-1 macrophage cell line and the KG-1 erythroleukemia cell line, with the fold changes for CD47 were 0.3 (THP-1) and 0.55 (KG-1) in vitro [985]. Thus, triptolide exhibits multifaceted anti-cancer effects by modulating the tumor immune microenvironment, depleting immunosuppressive macrophages, promoting T cell activity, and inhibiting immune checkpoint proteins like PD-L1. It also impacts critical signaling pathways, such as NF-*κ*B, ERK1/2, and IL-6/STAT3, to reduce tumor progression and promote anti-cancer immunity. Although challenges exist due to its solubility and cytotoxicity, advancements in DDS have shown promise in improving its therapeutic application.

### Triptolide reverses tumor drug resistance

Triptolide holds promise for reversing drug resistance across cancers, including NSCLC, prostate, and liver cancers, by enhancing chemosensitivity through mechanisms such as down-regulation of P-gp at both mRNA and protein levels, inhibition of the HNF1A/SHH and Keap1/NRF2 pathways [986]. Triptolide influences the EGFR primarily through modulation of protein expression and signaling pathways; it down-regulates ADAM12 in pituitary adenoma (TtT/GF and AtT-20) cells, thereby inhibiting ADAM12/EGFR signaling, and directly targets EGFR in bladder cancer (5637) cells, disrupting the PI3K/AKT pathway, while also enhancing the efficacy of EGFR tyrosine kinase inhibitors in NSCLC (H1975) cells by up-regulating E-cadherin and down-regulating proteins associated with EMT, highlighting its potential as a therapeutic agent in overcoming drug resistance and improving treatment outcomes [983, 987–990]. Therefore, triptolide’s ability to down-regulate drug efflux pumps, inhibit survival pathways (e.g., HNF1A/SHH and Keap1/NRF2), and target the EGFR/PI3K/AKT axis establishes its potential for overcoming chemo-resistance and targeted therapies resistance. Notably, its synergy with EGFR tyrosine kinase inhibitors in NSCLC highlights its value in combination therapies.

### Triptolide attenuates cancer cells by regulating the process of autophagy and ferroptosis

Triptolide has been shown to exhibit antitumor effects in ovarian cancer (SK-OV-3 and SK-OV-3/DDP) cells by inducing autophagy through ROS generation and JAK2/STAT3 pathway inhibition, enhancing chemotherapy sensitivity and inhibiting tumor growth, as well as by inducing apoptosis and reducing the protein expression of MMP-2, Sorcin, and VEGF, thereby disrupting the Mcl-1/Beclin1 interaction and highlighting its multifaceted therapeutic potential [991, 992]. It also alters the autophagic protein expression, notably reducing mTOR and AKT levels [993]. Furthermore, triptolide induces glioma cell growth via autophagy through ROS/JNK activation and inhibition of the AKT/mTOR signaling pathway. Besides, it can also induce autophagy in glioma (U87 and U251) cells, as evidenced by increased LC3-II/LC3-I ratio, upregulating protein expression of Beclin-1 and ATG7, and a notable increase of autophagosomes number observed via immunofluorescence [994]. Interestingly, triptolide is found to enhance the sensitivity of tumor necrosis factor-related apoptosis-inducing ligand (TRAIL) in pancreatic cancer cells by autophagy activation through Pumilio RNA-binding family member 1 (PUM1) downregulation, suggesting a potential strategy to increase tumor sensitivity to TRAIL [995]. Therefore, triptolide demonstrates versatile anti-cancer effects across various cancer types, highlighting its potential as a promising therapeutic agent with broad-spectrum activity against tumors.

Triptolide induces ferroptosis in various cancers by modulating key pathways at both gene and protein levels; it downregulates NRF2 in leukemia (K-562 and HL-60) cells, increasing ROS and lipid oxidation to sensitize cells to doxorubicin (DOX), inhibits GPX4 in glioblastoma (U-87 MG and U251-MG) cells through the NRF2/SLC7A11/GPX4 axis to promote lipid peroxide accumulation, and enhances ferroptosis in NSCLC (A549) cells by increasing ROS via the NF-*κ*B pathway while simultaneously reducing drug resistance proteins such as P-gp and GPX4 [977, 996–998]. Triptolide nano-MOFs induce cancer cells ferroptosis, which releases a lot of DAMPs, allowing DCs to present antigens and to multiply into CTLs, thereby preventing lung metastasis of melanoma [977]. An acid and GSH dual-controlled nanoplatform enhances cancer treatment by using triptolide to regulate NRF2 and inhibit GSH protein expression, reducing GPX4 activity and promoting ferroptosis, while minimizing toxicity [999]. However, one of its adverse effects is triptolide-induced cardiotoxicity, which involves ferroptosis [1000]. Although triptolide holds promise as a potential therapeutic agent, its cardiotoxicity associated with ferroptosis underscores the importance of targeted tumor approaches to mitigate adverse effects.

### Triptolide suppresses cancer progression via classical modalities

Triptolide can regulate the cell cycle in various cancer types, including breast, lung, prostate, and liver cancers. Triptolide exerts its effects by inducing cell cycle arrest at the G0/G1 phase, preventing the transition to the S phase, which inhibits cancer cell proliferation. Additionally, it can induce G2/M phase arrest, disrupting mitosis and promoting apoptosis. Triptolide exerts significant effects on the cell cycle across various cancer types, predominantly inducing G0/G1 phase arrest by enhancing the expression of cell cycle inhibitors in multiple myeloma (RPMI 8266) cells and suppressing proliferating cell nuclear antigen and Cyclin D1 in airway smooth muscle (ASMCs) cells in response to PDGF stimulation, thereby decreasing cell proliferation [1001, 1002]. In bladder cancer cells, the combination of triptolide and hydroxycamptothecin significantly enhances G0/G1 phase arrest by inhibiting CDK4, CDK6, and Cyclin D1. It also suppresses laryngocarcinoma HEp-2 cell proliferation by inducing G0/G1 phase arrest and apoptosis via Caspase activation and increased p53 stability. In NSCLC, triptolide further strengthens this arrest by reducing ribosomal protein L4, which disrupts the MDM2-p53 signaling pathway, thereby boosting its effects on inhibiting proliferation and promoting apoptosis [1003, 1004]. Additionally, triptolide induces G1 phase arrest in esophageal squamous cell cancer (KYSE150 and KYSE180) cells by regulating Cyclin D1 and CDKs [1005]. In triple negative breast cancer (BT-549 and MDA-MB-231) cells, human nasopharyngeal carcinoma (NPC-TW 039 and NPC-TW 076) cells and paclitaxel-resistant A549 cells, triptolide induces S phase arrest by causing DNA strand breaks, suppressing cyclin A-CDK2 complex formation and enhancing chemosensitivity to cisplatin and paclitaxel [1006–1008]. Furthermore, in glioma (U251) cells, triptolide triggers G2/M phase arrest while promoting apoptosis and autophagy, associated with the activation of ROS/c-JNK signaling pathways and inhibition of AKT/mTOR signaling [1009]. Collectively, these findings illustrate that triptolide modulates cell cycle progression primarily by inducing G0/G1 phase arrest through mechanisms such as enhancing cell cycle inhibitors and suppressing cyclins and CDKs, while also affecting S and G2/M phases, highlighting its potential as a multifaceted anti-cancer agent targeting diverse cell cycle regulatory pathways.

Triptolide's potent cytotoxicity indicates the influence in cancer therapy, and its principal mechanism is the induction of cell apoptosis. Triptolide demonstrates its effectiveness against gastric cancer (AGS and IM95) cells by increasing ROS levels, which trigger endoplasmic reticulum stress and induce cytoprotective autophagy, leading to the promotion of apoptosis. Additionally, in TNF-*α*-stimulated gastric cancer (AGS and MKN45) cells, triptolide enhances apoptosis via modulation of the H19/*miR-204-5p*/NF-*κ*B/FLIP axis, suggesting that its ability to induce apoptosis is multifaceted and includes both the promotion of ROS generation and the regulation of specific signaling pathways [1010, 1011]. The AS1411-triptolide conjugate shows promising potential as a targeted therapy for triple negative breast cancer, demonstrating high specificity and cytotoxicity against MDA-MB-231 cells, leading to significant tumor reduction and enhanced apoptosis in mouse models, with minimal toxicity to major organs [1012]. Additionally, triptolide promotes apoptosis and inhibits metastasis in glioblastoma cells in a dose-dependent manner by upregulating p21 and Bax protein expression while suppressing MMP-2 and MMP-9 protein levels, with PROX1 transcriptional inhibition elucidated as a mechanistic pathway, while in NSCLC, triptolide induces apoptosis and mitigates metastasis by targeting the IL-6/STAT3 signaling axis, characterized by reduced STAT3 phosphorylation and downregulation of STAT3 target genes, including c-Myc, Bcl-2, Mcl-1, and MMP-9, which are implicated in cell survival and migratory processes [978, 1013]. Thus, triptolide exhibits multifaceted anti-cancer effects by effectively inducing apoptosis and modulating critical signaling pathways across various cancer types.

### Triptolide combined with other medications for cancer treatment

Triptolide has demonstrated as a synergistic agent in combination therapies across various cancers, including lung and breast cancers, as well as renal cell carcinoma and nasopharyngeal carcinoma. Triptolide combined with first-line treatment for recurrent nasopharyngeal carcinoma significantly enhances therapeutic efficacy and improves patients’ life quality compared to single chemotherapy [1014]. In lung cancer, triptolide enhances the efficacy of EGFR tyrosine kinase inhibitors such as gefitinib, erlotinib, and icotinib, which are standard targeted therapies for NSCLC with EGFR mutation cell carcinoma [988]. These combinations highlight the versatility of triptolide in cancer therapy, effectively targeting key pathways and improving therapeutic outcomes. Given the disappointing outcomes of Bcl-2 inhibitor ABT-199 monotherapy in chronic lymphocytic leukemia, the combination of triptolide with ABT-199 has emerged as a promising strategy for acute myeloid leukemia treatment, as it enhances cytotoxicity by effectively targeting antiapoptotic proteins (Bcl-2 and Mcl-1), which may help improve therapeutic efficacy [1015]. Therefore, the combination of triptolide with chemotherapy, targeted therapies, and apoptosis-inducing agents has demonstrated significant potential in enhancing treatment outcomes, overcoming drug resistance, and improving patients' quality of life across various cancer types. The synergistic effects of triptolide when used alongside other medications, such as EGFR inhibitors in lung cancer, TRAIL receptor agonists in renal cell carcinoma, and Bcl-2 inhibitors in leukemia, further highlight its therapeutic promise.

### Triptolide derivatives inhibit cancer progression

Due to its water-insolubility and high cytotoxicity, triptolide derivatives have been developed to enhance the therapeutic efficacy and safety profile of the parent compound by addressing issues such as high toxicity and poor solubility. These modifications include mitochondrial-targeting strategies, such as conjugation with lipophilic cations (e.g., TPP^+^ or F16) for selective accumulation in tumor mitochondria, and biomimetic delivery systems like adipocyte-encapsulated triptolide derivatives combined with photosensitizers for melanoma treatment [1016]. Derivatives like LA67, triptolide-succinic acid ester, and compound 33 demonstrate increased bioavailability, targeted cytotoxicity, and innovative therapeutic options, including photodynamic therapy and NO-releasing capabilities, thus broadening triptolide's clinical application potential in cancer treatment [1017, 1018]. Additionally, biomimetic delivery systems like pTP-Ce6-Apo, where adipocytes encapsulate a palmitic acid-conjugated triptolide derivative (pTP) and a photosensitizer (Ce6), enable cytotoxic and photodynamic effects specifically in melanoma cells [1019]. Thus, triptolide derivatives can overcome the solubility and cytotoxicity limitations of the parent compound, enhancing bioavailability, targeting specificity, and reducing toxicity, while also providing innovative therapeutic modalities such as photodynamic therapy and optical imaging capabilities for improved cancer treatment outcomes.

### Application of multi-omics techniques to investigate the anti-cancer properties of triptolide

Triptolide exhibits significant anti-cancer effects as revealed through various omics studies. RNA sequencing and RNAPII-ChIP-Seq analyses demonstrate triptolide's biphasic impact on gene expression in pancreatic cancer (MIA PaCa-2) cells and cancer-associated fibroblasts, highlighting transcriptional down-regulation and specific alterations in histone acetylation [1020, 1021]. In an oncogenesis model (MCF10A-ErSrc), triptolide induces both down-regulation and up-regulation of tumorigenesis-associated genes, illustrating its complex regulatory influence [1022]. Metabolomics in CT26 tumor-bearing mice identifies alterations in amino acid and ketone body metabolism, with potential biomarkers indicating triptolide's multifaceted impact on tumor metabolism. Additionally, proteomics reveals 54 triptolide-interacting proteins, including Annexin A1 and Peroxiredoxin I/II, suggesting novel therapeutic strategies for colon cancer [1023]. Collectively, by employing a multi-dimensional omics strategy, the intricate effects of triptolide on cancer cells have been meticulously characterized. The synthesis of this comprehensive analysis positions triptolide as a potent candidate for oncotherapy, harnessing its mechanisms that span across transcriptional modulation, metabolic reengineering, and the precise engagement of oncogenic proteins. By taking these three steps, precision oncology can advance in novel ways.

### Investigating triptolide anti-cancer effects through drug delivery system

Recent developments in nanocarrier systems for triptolide have significantly advanced the treatment of tumor, such as liver cancer and malignant mesothelioma and leukemia. One approach involves a biomimetic nano-system that combines cancer cell-platelet hybrid membranes to co-deliver sorafenib and triptolide, achieving targeted delivery and prolonged circulation, which enhances tumor cell apoptosis and synergistic effects [1024]. Another innovation is the platinum/gold bimetallic nanoshell-coated triptolide liposomes (Pt@Au-TP-Lips), which utilize near-infrared light for chemophotothermal therapy, demonstrating higher photothermal conversion efficiency and substantial tumor growth inhibition [1025]. Additionally, a pH/ROS dual-responsive nanoparticle system for co-delivery of TP and the photosensitizer Chlorin e6 (Ce6) amplifies the effects of chemo-photodynamic therapy by enhancing oxidative stress and facilitating drug release in the tumor microenvironment [1026]. Moreover, an antibody–drug conjugate combining the anti-CD26 monoclonal antibody YS110 with triptolide (Y-TR1) selectively targets CD26-positive cancer cells (malignant mesothelioma and leukemia), effectively inhibiting RNA polymerase II activity [1027]. Together, these innovative nanocarrier strategies highlight the potential for improving liver cancer treatment through enhanced therapeutic efficacy and reduced toxicity. To address the limitations of triptolide's pharmacokinetics and toxicity, a novel antibody–drug conjugate, Cet-triptolide, is developed for targeted delivery to EGFR-overexpressing cancers, showing enhanced tumor-specific cytotoxicity and reduced toxicity compared to triptolide or cetuximab alone [987]. The triptolide nanoparticle with a CRPPR peptide-modified micelle to target the tumor microenvironment, following the priming action of a-mangostin-loaded nanoparticles that target CAFs and disrupt TGF-*β* signaling, leading to robust tumor growth suppression through a sequential targeting approach [1028]. The development of galactosylated-chitosan-triptolide-nanoparticles (GC-TP-NPs) for liver cancer enhanced antitumor efficacy by inducing apoptosis through the TNF/NF-*κ*B/Bcl-2 pathway, while minimizing systemic side effects [1029]. Overall, nanocarrier systems for triptolide, including antibody–drug conjugates and targeted nanoparticles, show great promise in enhancing therapeutic efficacy and minimizing toxicity. Future research should focus on optimizing these delivery methods to maximize triptolide's potential in clinical oncology.

Overall, triptolide orchestrates multi-front tumor suppression by reshaping the immune microenvironment, subverting drug resistance, and provoking autophagy-or ferroptosis-related cell death. It tampers with key signals, including IL-6/STAT3, NF-κB, and ERK1/2, to limit immunosuppressive macrophages and disable cancer-cell survival pathways. Although its inherent toxicity constrains clinical use, novel derivatives and smart delivery vehicles (e.g., nano-formulations and antibody–drug conjugates) have expanded triptolide’s therapeutic window. Multi-omics analyses further reveal extensive transcriptional, metabolic, and protein-network remodeling, suggesting deeper translational opportunities across diverse malignancies.

### Ursolic acid (UA)

UA, a hydroxy pentacyclic triterpene acid, is prevalent in various plants such as Crataegi fructus or *Crataegus pinnatifida* (Shanzha in Chinese) [1030, 1031]. This compound showcases a wide array of pharmacological effects, including anti-cancer, anti-bacterial, antioxidant, and anti-diabetic properties [1032]. It is particularly noted for its extensive anti-cancer capabilities, proving effective against a diverse range of cancers, including lung, breast, colon, prostate, stomach, and liver cancers, as well as melanoma [899, 1033]. The robust anti-cancer properties of UA are ascribed to its capacity to suppress tumor cell proliferation, facilitate apoptosis, induce cellular autophagy, and specifically target drug-resistant cancer cells [1034, 1035]. In silico analysis of UA binding to key EMT markers, including Snail, Slug, and Fibronectin, using quantum-polarized-ligand (QM/MM) docking, shows strong binding affinities and interactions, supporting its potential as an anticancer agent by regulating these markers in breast cancer [1036]. Moreover, in zebrafish models, UA significantly inhibits the growth and metastasis of breast cancer without causing detectable toxicity, supporting its potential as a therapeutic agent for breast cancer [1037]. Currently, UA nanoliposomes are undergoing clinical trials for various applications, including treatments for liver cancer (CTR20230593), gastric cancer (CTR20140180), and colon cancer (CTR20140395). The following discussion will concentrate on UA's anti-cancer properties and mechanisms.

### UA improves tumor immunity

UA has shown promising immunomodulatory effects in breast and colon, liver cancers by interacting with a variety of immune cell types and in modulating the tumor microenvironment [1038, 1039]. In breast cancer, UA inhibits cancer progression by augmenting the population of immune cells, promoting apoptosis, and restraining tumor-promoting immune cells, thereby regulating the tumor microenvironment [1040–1042]. It also inhibits breast cancer (4T1) tumor growth by reducing the number of Treg and MDSCs in tumor tissue through inhibiting STAT5 phosphorylation and IL-10 secretion [1043]. In addition, UA improves the tumor microenvironment by regulating tumor-associated fibroblasts (CAFs) in the tumor microenvironment, and it inhibits proliferation, migration and invasion of cancer cells by down-regulating the secretion of CXCL12 and decreasing the protein expression of CXCR4 and CXCR7 on the surface of colon cancer (HCT 116 and SW480) cells [1044]. Therefore, by targeting immune cell populations such as Tregs and MDSCs and modulating signaling pathways that regulate tumor cell migration and immune suppression, UA offers a promising strategy for enhancing anti-tumor immunity.

### UA reverses tumor drug resistance

UA exhibits the ability to reverse multidrug resistance in cancer cells through various mechanisms. It can enhance the anti-tumor effects of drugs like first-line molecular drugs (adriamycin, cisplatin, and paclitaxel) and targeted drugs (sorafenib) in breast cancer (MCF-7 and MDA-MB-231), ovarian cancer (SK-OV-3 and A2780), osteosarcoma (HOS and 143B), lung cancer (A549), and liver cancer (Hep G2) cells [1045].

Adriamycin remains a prevalent therapeutic agent for breast cancer (MCF-7/ADR); however, MDR poses significant challenges in its efficacy, with UA emerging as a potential modulator of this resistance [1046]. Furthermore, UA has demonstrated the capacity to potentiate adriamycin’s anti-tumor effects by inhibiting proliferation and migration of breast cancer (MDA-MB-231/DOX and MCF-7/ADR) cells, as well as by inducing mitochondrial dysfunction via the AMPK/mTOR/PGC-1*α* signaling pathway [1047]. Besides, in studies on adriamycin resistance in ovarian cancer, UA has been linked to the HuR/MDR1 axis, where it not only mitigates resistance but also enhances the sensitivity of ovarian cancer (SK-OV-3 and A2780) cells to adriamycin [1048]. UA enhances the anti-tumor effects of cisplatin through multiple mechanisms. In osteosarcoma (HOS and 143B) cells, UA promotes autophagic degradation of ferritin, leading to iron overload, lipid peroxidation, and ferroptosis, thereby increasing cisplatin's effectiveness [1049]. Additionally, in lung cancer (A549) cells, UA inhibits the JAK2/STAT3 pathway, reducing cancer stem cell marker expression and tumor stem cell populations, which sensitizes these cells to cisplatin [1050]. Paclitaxel is also a common drug used in the treatment of breast cancer. UA enhances paclitaxel sensitivity in breast cancer (MDA-MB-231/PTX) cells by up-regulating *miR-149-5p*, which down-regulates MyD88 protein expression and reverses drug resistance [1051]. Additionally, by down-regulating ING5 protein expression and inhibiting the PI3K/AKT signaling pathway, UA reverses liver cancer (Hep G2) cells resistance to sorafenib [1052]. Overall, UA demonstrates a broad capacity to reverse multidrug resistance in various cancer types, thereby enhancing the efficacy of common chemotherapy and targeted drugs. Through mechanisms such as inhibition of drug efflux pumps, modulation of metabolic pathways, regulation of miRNAs, and manipulation of key signaling pathways, UA sensitizes cancer cells to drugs like adriamycin, cisplatin, paclitaxel, and sorafenib.

### UA suppresses cancer growth by regulating the processes of autophagy and ferroptosis

UA exhibits a unique mechanism of action inhibiting cancer growth, which is particularly critical through the regulation of autophagy and ferroptosis processes. Growing evidence shows that UA induces autophagy and ferroptosis in esophageal squamous cell carcinoma (TE-8 and TE-12), colon cancer (HCT-8/5-FU), breast cancer (MDA-MB-231 and BT-549) and osteosarcoma (HOS and 143B) cells [899, 1053]. One of the key mechanisms of UA involves regulating autophagy, a cellular process that can either promote or inhibit cancer progression, depending on the cellular environment and the type of cancer [1054, 1055]. UA regulates live ROS-mediated autophagy through the AKT/mTOR signaling pathway, and acts on esophageal squamous cell carcinoma (TE-8 and TE-12) cells to inhibit proliferation, migration, invasion, and induce apoptosis [1056]. Furthermore, UA regulates autophagy by inhibiting mTOR phosphorylation, and acts on 5-FU-resistant human colon cancer (HCT-8/5-FU) cells to induce apoptosis and autophagy, inhibit proliferation, and reverse multidrug resistance in cancer cells, which provides a rationale for colon cancer treatment [1057]. It also inhibited autophagosome maturation in breast cancer (MCF-7) cells, impaired cellular lipid homeostasis and lysosomal membrane integrity, and combined with cationic amphiphilic drugs could enhance the killing effect on breast cancer [1058].

UA inhibits the proliferation of triple-negative breast cancer (MDA-MB-231 and BT-549) and progression of osteosarcoma (HOS and 143B) cells by inducing ferroptosis [1049, 1059]. UA induces iron death by inhibiting the NRF2 pathway and acts on MDA-B-231 and BT-549 cell-derived triple-negative breast cancer stem-like cells (BCSCs), effectively reducing their stemness characteristics and proliferative capacity, and inhibiting the cell proliferation of BCSCs in vitro and in vivo [1059]. In osteosarcoma (HOS and 143B), UA acts synergistically with cisplatin to activate autophagic degradation of ferritin, leading to intracellular accumulation of iron ions, generating lipid peroxidation and triggering iron death [1049]. Thus, UA promotes autophagic cell death and triggers ferroptosis, establishing it as a promising therapeutic agent in cancer treatment. By modulating critical pathways like AKT/mTOR in autophagy and NRF2 in ferroptosis, UA exhibits substantial anti-cancer effects across various types of cancer.

### UA suppresses cancer progression via classical modalities

UA regulates cancer cell proliferation through inducing cell cycle at G0/G1, S, and G2/M phases in various cancer types, including breast, prostate, colon, and liver cancers. UA inhibits cancer progression through the regulation of classical ways like inducing cell cycle arrest and cell apoptosis [1060, 1061]. UA inhibits breast cancer (MCF-7) cell proliferation by up-regulating protein expression of p53 and p21, and down-regulating that of CDK4, Cyclin D, CDK2, and cyclin E in the G0/G1 phase [1062]. In NSCLC (A549 and H460) cells, UA inhibits the EGFR/JAK2/STAT3 pathway to reduce tumor angiogenesis, migration and invasion, promote apoptosis and induce cell cycle arrest at G0/G1 phase [1063]. In colon cancer (SW480 and HCT 116), UA induces cell cycle arrest at the G0/G1 phase and promotes cell apoptosis. It enhances apoptosis by activating ROS and up-regulating the protein expression of Bax and down-regulating Bcl-2 protein level [1064]. In breast cancer (MCF-7), it induces cell cycle arrest at the G2/M cell phase, increases ROS levels and inhibits the PI3K/AKT signaling pathway, thereby promoting apoptosis, and also accelerates apoptotic processes by down-regulating Bcl-2 and up-regulating Bax protein expression [1034]. UA inhibits papillary thyroid carcinoma (IHH-4 and TPC-1) cells by classical mechanisms, which inhibits EMT by down-regulating fibronectin-1 (FN1), blocks invasive behaviors of cancer cells, reduces the protein expression of Bcl-2, and activates Caspase-3, which further promotes apoptosis [1065]. Therefore, UA demonstrates potent anti-cancer effects through classical mechanisms, primarily by inducing cell cycle arrest and promoting apoptosis. By modulating key signaling pathways such as p53, PI3K/AKT, EGFR/JAK2/STAT3, and Bax/Bcl-2, UA effectively inhibits cancer cell proliferation, migration, and invasion, while enhancing apoptosis.

### UA combined with other medications for cancer treatment

UA can play a role in colon, stomach, prostate, liver, and lung cancer cells by combining first-line chemotherapeutic agents and natural products [1066]. UA is combined with other drugs in the treatment of cancer, it mainly works by inhibiting cancer cell migration, inducing apoptosis and mitochondrial autophagy, inhibiting the expression of tumor stem cell markers, increasing the anti-tumor efficacy of the drugs, and decreasing drug resistance in cancer cells [1067–1069]. UA is combined with the first-line chemotherapeutic drug Adriamycin, it can target and inhibit the AKT signaling pathway and activate the Hippo signaling pathway, reducing drug resistance, increasing apoptosis, inhibiting colon cancer (HCT 116) cell migration, and decreasing tumorigenesis [1069, 1070]. UA can act in combination with sorafenib on a variety of human cancer (e.g., AGS, HCT 116, PC-3, Hep G2, and SMMC-7721) cells. It has potential anti-tumor effects in vivo by influencing the expression of the anti-apoptotic protein Mcl-1, reducing the potential of the mitochondrial membrane, triggering autophagy, and other mechanisms [1071, 1072]. Additionally, combining UA with oleanolic acid can down-regulate the MMPs pathway to inhibit colon cancer (HT-29 and SW620) cell migration and enhance cancer cell death by inducing mitochondrial autophagy [1067]. This combination also potentially inhibits cancer cells by down-regulating the AKT/mTOR signaling pathway, activating the antioxidant response of NRF2 and affecting the intracellular autophagy mechanism in lung cancer (A549) cells [1068]. Thus, by enhancing the effects of chemotherapeutic agents such as adriamycin and sorafenib, and natural compounds like oleanolic acid, UA contributes to the inhibition of cancer cell migration, induction of apoptosis, and mitochondrial autophagy. Additionally, UA's ability to target key signaling pathways, including AKT, mTOR, and NRF2, enhances the anti-tumor efficacy of the combination therapies, while also reducing drug resistance.

### UA derivatives inhibit cancer progression

Notable derivatives such as UA232 and UA derivatives substituted at the C-3 position are fascinating advancements that expand UA's potential in fighting breast and cervical cancers. Such structural modifications increase the selectivity and potency of these compounds, presenting new potential directions and applications for cancer therapy [1073–1077]. UA232 is chemically modified and synthesized to inhibit lung cancer (H460 and A549) cell proliferation by activating the PERK-eIF2a-CHOP pathway and inducing endoplasmic reticulum stress [1078]. In addition, UA232 activates the PERK/ATF4/CHOP pathway, triggers endoplasmic reticulum stress-associated apoptosis, and promotes lysosome-dependent cell death by increasing lysosomal membrane permeability, thereby effectively inhibiting the proliferation of breast cancer (MCF-7) and cervical cancer (HeLa) cells [1075]. Besides, a series of UA derivatives with different amino acid and dipeptide substitutions at the C-3 position, MDA-MB-231, are synthesized, and some of these derivatives (e.g., 4c, 7a, 10a) shows high cytotoxicity against triple-negative breast cancer (MDA-MB-231) cells, and inhibits cancer cell proliferation and migration, induces apoptosis and autophagy through various pathways [1079]. Therefore, UA derivatives, including UA232 and C-3 substituted derivatives, significantly expand the therapeutic potential of UA in cancer treatment. These derivatives exhibit enhanced potency and selectivity, enabling them to effectively inhibit cancer cell proliferation, migration, and metastasis through mechanisms such as the activation of the PERK/eIF2*α*/CHOP pathway, induction of ER stress, and lysosome-dependent cell death.

### Application of multi-omics techniques to investigate the anti-cancer properties of UA

The application of multi-omics techniques has brought new research opportunities for UA research. UA inhibits breast cancer (MCF-7) cell proliferation through multilevel regulation. Metabolomic and epigenetic analyses showed that UA modulates metabolite levels, alters gene methylation patterns, and affects key signaling pathways such as RAF/ERK and IKK/NF-*κ*B in cancer cells [1080]. In prostate cancer (VCaP) cells, UA regulates critical metabolites and signaling pathways, such as S-adenosylmethionine (SAM), glycolysis, and phospholipid metabolism, while RNA sequencing indicates UA’s impact on pathways related to cancer metastasis and oxidative stress [1081]. As a result, by regulating key metabolites, gene methylation patterns, and signaling pathways involved in cancer cell growth, metabolism, and metastasis, UA shows promise as a multifaceted therapeutic agent.

### Investigating UA anti-cancer effects through drug delivery system

UA has potential for clinical application as an antitumor agent; however, effective targeting of tumors remains challenging due to limitations such as poor water solubility, rapid plasma clearance and low targeting efficiency. To overcome these challenges, recent studies have focused on improving the therapeutic efficacy of UA in colon, breast and lung cancers using DDS via nanoparticle encapsulation, polymeric micelles and hydrogels [1082]. Nanoparticle-based UA drug delivery system UA/(AS-IV)@PDA-HA synergistically inhibits the proliferation and metastasis of NSCLC (CTC-TJH-01 and A549) cells by chemotherapy, PTT and immunotherapy, demonstrating good biocompatibility and tumor targeting [1083]. UA can be synthesized into polyursolic acid (PUA) by polycondensation reaction, and the self-assembly of this PUA with paclitaxel can form PUA-NPs@PTX nanoparticles, and this PUA-NPs@PTX can be retained for a longer period of time in CT26 hormone mice, which has strong anti-tumor effects on colon cancer (CT26) cells [1084]. Furthermore, UA and the pharmaceutical agent 5-fluorouracil (5-FU) can be utilized in a nano-delivery system, which is prepared through a pore space allocation strategy to regulate the release of both substances. This approach consequently results in a more pronounced inhibitory effect on breast cancer (4T1) cells [1085]. Besides, in breast cancer cell (MCF-7/ADR), the inhibitory effect of adriamycin hydrochloride on drug-resistant tumors is enhanced by constructing micelles with mitochondria-targeting and ROS-sensitive functionality (TDTD@ UA/HA) and inhibits the proliferation of drug-resistant breast cancer cells [1086]. In the hydrogel delivery system, UA can be constructed with cisplatin (DDP) as a co-loaded pectin/cellulose hydrogel, which acts on mouse lung cancer (LA795) cells to inhibit the proliferation, migration and promote apoptosis of lung cancer cells by inhibiting the TMEM16A, regulating the MAPK and EMT signaling pathways and other mechanisms [1087]. Therefore, nanoparticle encapsulation, polymeric micelles, hydrogels, and other formulations have addressed the challenges of poor solubility, rapid clearance, and low targeting efficiency, making UA a more viable option for clinical use. These innovative delivery systems improve the bioavailability and targeted delivery of UA to tumor sites, enhancing its anti-cancer effects in various cancer types.

In sum, UA orchestrates diverse anti-cancer mechanisms from reconfiguring the tumor immune landscape and reversing multidrug resistance to provoking autophagy, and ferroptosis-driven cell death. By modulating critical pathways (e.g., AKT/mTOR, JAK/STAT, and NRF2) in tandem with Tregs, MDSCs, and fibroblasts, it dampens tumor progression while enhancing chemotherapy responsiveness. Recent innovations in nano-formulations and hydrogels circumvent its solubility barriers, enabling potent synergy with conventional therapies. Multi-omics evidence underscores UA’s broad regulatory impact, emphasizing its potential for robust tumor suppression across multiple cancers.

## Conclusion

This review highlights the potential of natural anti-cancer products derived from herbal medicine, underscoring their diverse pharmacological properties and mechanisms of action. Compounds such as apigenin, artemisinin, berberine, and curcumin, among others, have demonstrated significant immunomodulatory, anti-proliferative, pro-apoptotic, and anti-metastatic effects, both in vitro and in vivo. These natural products hold promise for the development of novel anti-cancer therapies, potentially as standalone treatments or in synergy with existing therapies to enhance efficacy and reduce side effects. They exhibit multi-target and multi-mechanism anti-cancer actions, including direct cytotoxicity against tumor cells, suppression of tumor growth and metastasis, immunomodulation, and enhancement of chemotherapy effectiveness, along with reduced treatment-related side effects.

However, several challenges remain, including variability in the quality of herbal extracts, limited clinical data supporting their effectiveness, and the need for more rigorous pharmacokinetic and pharmacodynamic studies. Addressing the bioavailability and pharmacokinetic limitations of these natural compounds is also crucial for improving their targeted therapeutic effects on tumor tissues. Emerging DDS, such as nanoparticles, micelles, and affinity hydrogels, have successfully enhanced the targeting and efficacy of certain natural products like UA.

In addition, integrating multi-omics technologies has allowed deeper insights into the molecular networks underlying the actions of natural compounds. For instance, metabolomics and methylation analyses have revealed the metabolic reprogramming and epigenetic regulatory effects of UA on cancer cells. The research methodologies typically employed in oncology pharmacology—including efficacy assays, omics predictions, target verification, and drug modification—culminate in integrating the unique characteristics of these compounds with advanced DDS to optimize clinical application. Figure 3 presented in this paper provides a standardized research strategy to guide future investigators.

Until now, small molecules in herbal medicines have been relatively well-studied, but they are very low proportion of the components in herbal medicines. Even though polysaccharides have captured significant research interests, numerous components within herbal medicines, including proteins, lipids, and nucleic acids, await comprehensive exploration. Emerging evidence indicates that the plant-derived exosomes have promising avenues and offer unique opportunities for the innovation and development of herbal medicines. Future studies should prioritize standardized extraction protocols, rigorous clinical trials, mechanistic studies, and the application of machine learning to fully harness the therapeutic potential of natural products from herbal medicines. Ultimately, these efforts aim to integrate natural substances into standard cancer treatment regimens, providing a holistic approach that complements and enhances contemporary oncological practices. The fusion of traditional herbal wisdom with advanced scientific technologies promises a new chapter in cancer therapy, potentially improving patient survival rates and quality of life.

## Data Availability

Not applicable.

## Abbreviations

ABCB1: ATP-binding cassette subfamily B member 1
ACSL4: Acyl-CoA synthetase long chain family member 4
AFAP1L2: Actin filament associated protein 1-like 2
AMPK: AMP-activated protein kinase
ATGs: Autophagy-related genes
Bad: Bcl-2-associated death promoter
Bak: Bcl-2 homologous antagonist/killer
Bax: Bcl-2-associated x protein
Bcl-2: B-cell lymphoma 2
Bcl-xL: B-cell lymphoma-extra large
BCRP: Breast cancer resistance protein
BCS: Biopharmaceutical classification system
BDMC: Bisdemethoxycurcumin
Bim: Bcl-2-interacting mediator of cell death
CACC: Colitis-associated colon cancer
CCL2: C–C motif chemokine ligand 2
CDF: Curcumin difluorinated
CDK: Cyclin-dependent kinase
CML: Chronic myelogenous leukaemia
COX-2: Cyclooxygenase-2
CTL: Cytotoxic T lymphocyte
CTLA-4: Cytotoxic T-lymphocyte-associated protein 4
CXCL: C-X-C motif chemokine ligand
CXCR: C-X-C chemokine receptor type
DCs: Dendritic cells
DDS: Drug delivery systems
DMC: Demethoxycurcumin
DUSP1: Dual specificity phosphatase 1
EGF: Epidermal growth factor
EGFR: Epidermal growth factor receptor
EMT: Epithelial-mesenchymal transformation
Fas: Fatty acid synthase
GLUT1: Glucose transporter 1
GPAT1: Glycerol-3-phosphate acyltransferase 1
GPX4: Glutathione peroxidase 4
GSH: Glutathione, HAS2: Hyaluronan synthase 2
HER: Human epidermal growth factor receptor
HIF-1*α*: Hypoxia-inducible factor 1-alpha
HNSCC: Head and neck squamous cell carcinoma
HSP90: Heat shock protein 90
IL: Interleukin
iNOS: Inducible nitric oxide synthase
JNK: C-Jun n-terminal kinase
KRAS: Kirsten rat sarcoma viral oncogene homolog
LAMB3: Laminin subunit beta 3
LTC4: Leukotriene C4
MAPK: Mitogen-activated protein kinase
Mcl-1: Myeloid cell leukemia-1
MDSCs: Myeloid-derived suppressor cells
MMPs: Matrix metalloproteinases
MRP1: Multidrug resistance protein 1
mTOR: Mammalian target of rapamycin
mTORC1: Mammalian target of rapamycin complex 1
NCoR2: Nuclear receptor corepressor 2
NETs: Neutrophil extracellular traps
NF-*κ*B: Nuclear factor kappa-B
NK: Natural killer
NMR: Nuclear magnetic resonance
NRF2: Nuclear factor erythroid 2-related factor 2
PARP: Poly (ADP-ribose) polymerase
PBMCs: Peripheral blood mononuclear cells
PD-1: Programmed death-1
PDGF: Platelet-derived growth factor
PD-L1: Programmed death-ligand 1
PfMDR1: Plasmodium falciparum multidrug resistance protein 1
P-gp: P-glycoprotein
PI3K: Phosphoinositide 3-kinase
PTEN: Phosphatase and tensin homolog
PTHLH: Parathyroid hormone-like hormone
QSAR: Quantitative structure–activity relationship
ROS: Reactive oxygen species
RTKs: Receptor tyrosine kinases
SASP: Senescence-associated secretory phenotype
SCD: Stearoyl-coA desaturase
SDF-1: Stromal cell-derived factor 1
SHIP1: Src homology 2 domain-containing inositol phosphatase 1
SIK3: Salt-inducible kinase 3
SLC7A11: Solute carrier family 7 member 11
SMEDDS: Self-microemulsifying drug delivery system
SOX11: SRY-related HMG-box 11
STAT3: Signal transducer and activator of transcription 3
TAMs: Tumor-associated macrophages
TCF4: Transcription factor 4
TGF-*β*: Transforming growth factor-beta
THC: Tetrahydrocurcumin
TLR: Toll-like receptor
TNF-*α*: Tumor necrosis factor-alpha
TPGS: Tocopheryl polyethylene glycol 1000 succinate
Tregs: Regulatory T cells
TWIST1: Twist family BHLH transcription factor 1
ULK1: Unc-51 like autophagy activating kinase 1
VEGFR: Vascular endothelial growth factor receptor
WEE1: WEE1 G2 checkpoint kinases
